# Supplementary material for: Identification of the major rabbit and guinea pig semen coagulum proteins and description of the diversity of the REST gene locus in the mammalian clade Glires
Source: PLoS One. 2020 Oct 14;15(10):e0240607. doi: 10.1371/journal.pone.0240607 (PMC7556508; doi:10.1371/journal.pone.0240607)
Supplement: S1 Table — (DOCX) [file pone.0240607.s001.docx]

| **Guinea pig primers** | | |
| --- | --- | --- |
| **Primer name** | **Primer sequence** | **Use** |
| SVP51f | GCTCCTCATTTGGGTGAAGCAAGCATCT | PCR and sequencing primer |
| SVP52r | GTCCATTAACCCAAATGGAACCGATCAT | 5’ RACE and sequencing primer |
| SVP53r | AACCTCGACCTTTCACCATTCCAAGACT | 5’ RACE and sequencing primer |
| SVP54f | GTGCTAGGAAAGGTCACTCGCAGGTTAA | 3’ RACE and sequencing primer |
| SVP55f | AAGATCGTATGAAACGTCACCGGCAGTT | 3’ RACE and sequencing primer |
| SVP56f | GCATGAAGGTCAAAGGACAAACTCTTAT | Sequencing primer |
| SVP57r | CACTCAACATCACTCCCACACTTGGAAT | PCR and sequencing primer |
| SVP58r | CACAAAATCTTGTCCACCAACCCCAAT | Sequencing primer |
| SVP59f | GGTTCCTTGAAAGGTCATACCCAGGTGA | PCR and sequencing primer |
| SVP510r | GAACCCCGACCTTTCAGAGAACCATGAT | Sequencing primer |
| SVP511f | TCCTGTGAAAGGTCGCACACAAGGCCAA | PCR and sequencing primer |
| SVP512r | CTGCATTTGATCTTTTACAGAACTGAGA | PCR and sequencing primer |
| SVP513f | AAAGGCTCTAGTACTATGAAAAGCCTTA | Sequencing primer |
| SVP514r | GACCTTTGAGAGAACTTTGACTTCTCGA | Sequencing primer |
| SVP515r | CTGGGAAAGGGTAGTGCTACTAAGACTA | PCR and sequencing primer |
| SVP516f | GGGTTCTCTAAAAGGTCAGAGTTCT | Sequencing primer |
| SVP517r | TTAACTGAAACTGCTTGTCCCACGT | Sequencing primer |
| SVP518f | GGCTTTGGTCCTATTAAAGGTCATA | Sequencing primer |
| SVP519f | GGTCCAGATTCCTTGAAAGGTCGAA | PCR and sequencing primer |
|  |  |  |
| **PCR and RACE products used as sequencing templates** | | |
| **Product size** | **Primer pairs** | **Sequencing primers** |
| 3.3 kb | SVP51f + SVP512r | SVP51f, SVP52r, SVP53r, SVP56f, SVP58r,SVP510r,SVP512r, SVP516f,SVP517r,SVP518f,SVP519f |
| 2.5 + 3.1 kb | SVP511f + SVP57r | SVP54f, SVP55f, SVP57r, SVP512r, SVP513f, SVP514r |
| 2.0 kb | SVP59f + SVP512r | SVP59f, SVP510r, SVP512r |
| 1.4 kb | SVP519f + SVP512r | SVP519f,SVP511f, SVP512r |
| 0.6 kb | SVP55f + SVP515r | SVP55f, SVP515r |
| 0.3 kb | UPM^1^ + SVP52r | SVP52r |
| 1.1 kb | UPM^1^ + SVP54f | SVP54f, SVP55f |
| 0.7 kb | UPM^1^ + SVP55f | SVP55f |

^1^ UPM is the universal primer mix provided with the SMARTer RACE kit.
