# Supplementary material for: Identification of the major rabbit and guinea pig semen coagulum proteins and description of the diversity of the REST gene locus in the mammalian clade Glires
Source: PLoS One. 2020 Oct 14;15(10):e0240607. doi: 10.1371/journal.pone.0240607 (PMC7556508; doi:10.1371/journal.pone.0240607)
Supplement: S2 Table — (DOCX) [file pone.0240607.s002.docx]

| **Rabbit primers** | | |
| --- | --- | --- |
| **Primer name** | **Primer sequence** | **Use** |
| SVP200-f1 | GGTGAAAGTCAAGATTCAGTGGAAGAT^1^ | PCR and sequencing primer |
| SVP200-r2 | CTCTCTCACTGGACGATGATCCGTGTCA^1^ | 5’ RACE and sequencing primer |
| SVP200-f3 | GATCAGAAAGCCAGCGTAGATCACATT | PCR and sequencing primer |
| SVP200-r4 | GCTTCCAGACCAGAGACTGCAGCTTGT | PCR and sequencing primer |
| SVP200-f5 | CATTCCAGGTAAAGATCAACCAACTGT | 3’ RACE and sequencing primer |
| SVP200-r6 | CAGTATCTGGACCTTTCGCACTACCTT | PCR and sequencing primer |
| SVP200-f7 | GAGCAAGACTCTGCTAAAGGGCAGATT | Sequencing primer |
| SVP200-f9 | CGGCTCTTTCTGGCGAGATGAAGTA | PCR and sequencing primer |
| SVP200-10r | AGTTTACTCTCCCAAATAGCTACAA | PCR and sequencing primer |
| SVP200-11r | CTTCATTTCTTGACTGGTTATAGGAAT | Sequencing primer |
| SVP200-12r | CTGACCAGTCATACCAAGGTGTGT | Sequencing primer |
| SVP200-13r | TTCCTGACCACTTACAGGAGCTTCT | Sequencing primer |
| SVP200-15f | CACCTTGGTATGACTGGTCAGGAAT | Sequencing primer |
|  |  |  |
| **PCR and RACE products used as sequencing templates** | | |
| **Product size** | **Primer pair** | **Sequencing primer** |
| 3.1 kb | SVP200-1f + SVP200-6r | SVP200-1f, SVP200-6r, SVP200-7f, SVP200-12r, SVP20013r, SVP200-15f |
| 1.9 kb | SVP200-9f + SVP200-4r | SVP200-9f, SVP200-4r, SVP200-11r |
| 1.4 + 1.0 kb | SVP200-3f + SVP200-10r | SVP200-10r |
| 1.1 + 0.8 kb | SVP200-3f + SVP200 -6r | SVP200-3f on purified 0.8 kb product |
| 0.5 kb | UPM^2^ + SVP200-2r | SVP200-2r |
| 0.6 kb | UPM^2^ + SVP200-5f | SVP200-5f |
|  |  |  |

^1^ Mismatches between the primer and cDNA sequence are highlighted in red. The primers generated the expected products in spite of the mismatches.

^2^ UPM is the universal primer mix provided with the SMARTer RACE kit.
