# Supplementary material for: Identification of the major rabbit and guinea pig semen coagulum proteins and description of the diversity of the REST gene locus in the mammalian clade Glires
Source: PLoS One. 2020 Oct 14;15(10):e0240607. doi: 10.1371/journal.pone.0240607 (PMC7556508; doi:10.1371/journal.pone.0240607)
Supplement: S1 Fig — The cDNA sequence of Svp5 is shown with the translated protein sequence written above. The predicted signal peptide is highlighted in grey. Peptide sequences highlighted in yellow indicates that they agree with peptides generated by trypsin digestion of the 190 kDa component in the guinea pig seminal vesicle secretion. (DOCX) [file pone.0240607.s003.docx]

M K P T V F L I L S L L L I W V K Q A S G K K L L V A

AGGGCCCTTCCTGGCAAGATGAAGCCTACCGTCTTCCTCATCCTTTCCCTGCTCCTCATTTGGGTGAAGCAAGCATCTGGAAAGAAACTCCTTGTTGCTG

V K G Q E T V Q G Q V W T T G Q D P M E E E F I V Q R K D P M I G S

TTAAAGGACAAGAAACTGTGCAAGGTCAGGTTTGGACCACAGGACAGGATCCCATGGAAGAAGAATTTATAGTTCAACGTAAAGATCCCATGATCGGTTC

I W V N G Q G F V E E A V S V K G L S S M K G R M R V K G Q A L I

CATTTGGGTTAATGGACAAGGTTTCGTGGAAGAAGCAGTTTCAGTCAAAGGTCTCAGCTCTATGAAAGGTCGCATGCGAGTCAAGGGACAAGCTCTGATT

G K T V S V K G L H P V K G R M K V K G Q T L M G K A V S V K G H

GGAAAAACTGTCTCTGTCAAAGGTCTCCACCCTGTGAAAGGTCGCATGAAGGTCAAAGGACAAACTCTTATGGGAAAAGCAGTTTCTGTCAAAGGTCACG

G S L K S R M Q V K G Q D L M G E E F L V Q G N D P V I S H I W V N

GTTCTTTGAAAAGTCGCATGCAGGTCAAAGGACAAGATCTAATGGGAGAAGAATTTTTAGTTCAAGGTAATGATCCAGTGATCAGTCACATTTGGGTCAA

Q E D F V E N P V S V K S L G M V K G R G Y L K G H G Y L K G Q G

CCAAGAAGATTTCGTGGAAAACCCAGTTTCAGTTAAAAGTCTTGGAATGGTGAAAGGTCGAGGTTACCTGAAAGGTCATGGCTATCTGAAAGGTCAAGGT

S L K G Q L Q I K G Q D S M E E E I T V K G L Q P V K G P I Q V K

TCTCTGAAAGGTCAACTACAGATCAAAGGACAAGATTCCATGGAAGAAGAAATTACAGTTAAAGGTCTCCAACCTGTGAAAGGTCCCATACAGGTCAAAG

G Q D L M G E E F L V Q H T D P M I S H I W V D G Q D F M E E E V S

GACAAGATCTCATGGGAGAAGAATTTTTAGTTCAACATACAGATCCAATGATCAGTCACATTTGGGTCGATGGACAAGATTTTATGGAAGAAGAAGTTTC

D K G L S P V K G H M Q I K E Q D L E E A V S V K G V G T V K G P

AGATAAAGGCCTCAGTCCTGTGAAAGGTCACATGCAGATCAAAGAACAAGATCTGGAAGAAGCAGTGTCAGTTAAAGGTGTAGGCACTGTTAAAGGTCCA

G S L K G R G S L K G H L Q I K G Q D L D K A V S A K G L S P V K

GGTTCCCTGAAAGGTCGTGGTTCTTTGAAAGGTCACTTACAGATCAAAGGACAAGATCTGGATAAGGCAGTTTCAGCTAAAGGCCTCAGTCCTGTTAAAG

G R S S L K G Q G S L K G R G S L K G Q G S L K G S M Q I K G Q D L

GCCGCAGTTCTCTAAAAGGTCAGGGTTCTCTGAAAGGTCGGGGTTCCTTGAAAGGTCAGGGTTCCTTGAAAGGTAGTATGCAGATCAAAGGACAAGATCT

T G D K N L F Q G Q D P T N G H T W V G G Q D F V E E P D S V K S

CACAGGAGATAAAAATTTATTTCAAGGTCAAGATCCAACAAATGGTCACACTTGGGTTGGGGGACAAGATTTTGTGGAAGAACCAGATTCAGTTAAAAGC

L G P V K G G T Q I Q G Q D F L G E A V A V K G F G P I K G H S S

CTTGGTCCTGTAAAAGGTGGCACACAAATCCAGGGACAAGACTTCCTTGGAGAAGCAGTTGCAGTTAAAGGCTTTGGTCCTATTAAAGGTCATAGTTCTC

L K G R G S L K G L G S L K G Q S S Q K G R G S L K G Q G S L K G H

TAAAAGGTCGAGGTTCTCTGAAAGGTCTGGGTTCTCTAAAAGGTCAGAGTTCTCAGAAAGGTCGGGGTTCCTTGAAAGGTCAAGGTTCCTTGAAAGGTCA

T Q V K G Q D L M G E E P L V Q G Q D P T G G H I G V G G Q D F V

TACCCAGGTGAAAGGACAAGATCTCATGGGAGAAGAACCTTTAGTTCAAGGTCAAGATCCAACAGGTGGTCACATTGGGGTTGGTGGACAAGATTTTGTG

D E A D S V K G F S P V K G R T Q G Q E Q D V G Q A V S V K G F G

GATGAAGCAGATTCAGTGAAAGGCTTCAGTCCTGTGAAAGGTCGCACACAAGGCCAAGAACAAGACGTGGGACAAGCAGTTTCAGTTAAAGGGTTTGGTC

P V K G R G S L K G R G S L K G R S S L K G Q G S L K G R T Q V K G

CTGTTAAAGGTAGGGGTTCTCTAAAAGGTAGAGGTTCTCTGAAAGGTCGGAGTTCTCTAAAAGGTCAAGGTTCCTTGAAAGGTCGTACACAGGTCAAAGG

Q D L M G E E P L V Q G Q D P T G G H I G V G G Q D F V D E A D S

ACAGGACCTCATGGGAGAAGAACCTTTAGTTCAAGGTCAAGATCCAACAGGTGGTCACATCGGGGTTGGTGGGCAAGACTTTGTGGATGAAGCAGATTCA

V K G F S P V K R R T Q G Q E Q D L L G Q A V S V K G F G P V K G

GTGAAAGGCTTCAGTCCTGTGAAACGTCGCACACAAGGCCAAGAACAGGACCTCCTGGGACAAGCAGTTTCAGTTAAAGGCTTTGGTCCTGTTAAAGGTC

R G S L K G R G S L K S L G S L K G G G S L K G R G S L K G P D S L

GGGGTTCTCTGAAAGGTCGGGGTTCTCTAAAAAGTCTGGGTTCTCTAAAAGGTGGAGGTTCTCTGAAAGGTCGGGGTTCTCTGAAAGGTCCAGATTCCTT

K G R S S L K G R T Q V K G Q D L T G E E P L V Q G Q D P T G G H

GAAAGGTCGAAGTTCCTTGAAAGGTCGTACGCAGGTCAAAGGACAAGATCTCACCGGAGAAGAACCTTTAGTTCAAGGTCAAGATCCAACGGGTGGTCAC

I G V G G Q D F V K E T D S V K G F S P V K G R T Q G Q E Q D L G .

ATTGGGGTTGGAGGACAAGATTTTGTGAAAGAAACAGATTCAGTGAAAGGCTTCAGTCCTGTGAAAGGTCGCACACAAGGCCAAGAACAAGACCTGGGAC

Q A V S V K G F G P V K D H G S L K G R G S L K G L G S L K G G G S

AAGCAGTTTCAGTTAAAGGCTTTGGTCCTGTTAAAGATCATGGTTCTCTGAAAGGTCGGGGTTCTCTAAAAGGTCTGGGTTCTCTAAAAGGTGGAGGTTC

L K G R G S L K G H T Q L T G Q D L I G E E L L V Q G Q D P V V G.

TCTGAAAGGTCGGGGTTCCTTGAAAGGTCATACACAGCTCACAGGACAAGATCTCATAGGAGAAGAACTGTTAGTTCAAGGTCAAGATCCAGTGGTTGGT

.R Q H F V E E A G S A K G L S P I K G R S S L K G H G S L K G H L

AGACAACATTTTGTGGAAGAAGCAGGTTCAGCAAAAGGCCTTAGTCCTATTAAAGGTCGCAGTTCTCTGAAAGGTCACGGCTCCTTGAAAGGTCACCTAC

Q R K E D L D E A V L V K G L H P V K G R T Q V Q E Q D L L G Q A V

AGAGGAAAGAAGATCTGGATGAAGCAGTTTTAGTTAAAGGTCTCCATCCTGTGAAAGGTCGCACACAAGTTCAAGAACAGGACCTCCTGGGACAAGCAGT

S V K G F G P V K G Q G S L K G P I Q V K G Q D L L G E A V S V K

TTCAGTTAAAGGCTTTGGTCCTGTTAAAGGTCAAGGTTCTCTGAAAGGTCCCATACAGGTCAAAGGACAAGACCTCTTGGGAGAAGCAGTTTCAGTTAAA

G L G P V K S H G A L K G Q G S L K G P I Q V K G Q D L L G E A V .

GGGCTCGGTCCTGTGAAAAGTCATGGTGCTCTGAAAGGTCAAGGTTCTCTGAAAGGTCCCATACAGGTCAAAGGACAAGACCTCTTGGGAGAAGCAGTTT

S V K G I S P V K S H G A L K G H D S L K D H L Q M K G Q D L N E A

CAGTTAAAGGCATCAGTCCTGTGAAAAGTCATGGTGCTCTGAAAGGTCACGATTCTTTGAAAGATCACCTACAGATGAAAGGACAAGATCTAAATGAAGC

. V S V K G L S P V K G G I Q V P G Q D I L G E A V L V K G L T P V

AGTGTCCGTTAAAGGGCTCAGCCCTGTGAAAGGTGGCATACAGGTACCGGGGCAAGACATCCTGGGAGAAGCAGTTTTGGTTAAAGGTCTCACGCCTGTG

K D G T Q I Q G Q D L L A E A I S I K G L A P G K C G T Q V E G Q .

AAAGATGGCACACAGATCCAAGGGCAAGATCTCCTGGCAGAAGCAATTTCGATTAAAGGTCTCGCTCCTGGAAAATGTGGCACACAAGTCGAAGGGCAAG

D F L G E A A L V K G L A P E K G G T Q V Q G Q D V L G D A L S V K

ACTTCCTGGGAGAGGCAGCTTTGGTTAAAGGTCTCGCCCCTGAGAAAGGTGGCACACAGGTCCAAGGGCAAGACGTCCTAGGAGATGCACTTTCAGTTAA

G L L P V K G D M G I K G Q A L I P E A V K G S S T M K S L M Q L

AGGTCTCCTTCCTGTGAAAGGTGACATGGGAATCAAAGGACAAGCTCTCATACCAGAAGCAGTTAAAGGCTCTAGTACTATGAAAAGCCTTATGCAGCTC

K G Q D V M R E A I A V K G L S S V K D Q M Q V N G Q N L M E V V

AAAGGACAAGATGTTATGAGAGAAGCAATTGCAGTTAAAGGTCTCAGTTCTGTAAAAGATCAAATGCAGGTCAACGGACAAAATCTCATGGAAGTAGTTG

A V K T V S S T K G H I Q V T E R D S M G E F F S Q H K D P T I G H

CAGTTAAAACAGTCAGTTCTACAAAAGGTCATATACAGGTCACAGAACGAGATTCCATGGGAGAATTTTTCAGTCAACATAAAGATCCCACGATCGGTCA

I W V T G Q D F V Q E S V S V K G L D S V K G E M R V K G Q N F V.

CATTTGGGTCACAGGACAAGATTTCGTGCAAGAATCAGTTTCAGTGAAAGGTCTAGATTCTGTGAAAGGTGAAATGCGAGTCAAAGGACAAAATTTCGTT

.G E A V S V K G L D S V K G Q M R V K G Q H F L G E A V S V K S V

GGAGAAGCTGTTTCAGTGAAAGGTCTAGATTCTGTGAAAGGTCAAATGAGGGTAAAAGGACAACATTTCCTTGGAGAAGCTGTTTCAGTGAAAAGTGTTA

S S V K D C M Q I K G Q D F T G E A V S V E G L D S V K G H M R V K

GTTCTGTGAAAGACTGTATGCAAATCAAAGGACAAGATTTCACTGGAGAGGCAGTTTCAGTTGAAGGTCTCGATTCTGTGAAAGGTCACATGAGGGTCAA

G Q N F I G E A V S V K D L G S V K S H M Q Y K G Q D F L G E A V.

AGGACAAAATTTCATTGGAGAAGCAGTTTCAGTTAAAGATCTTGGTTCTGTTAAAAGTCACATGCAGTACAAAGGACAAGATTTCCTTGGAGAAGCTGTT

.S V K G L H P V K G D T Q V K E Q G L L D K A V S V K G L S S V E .

TCAGTTAAAGGTCTCCATCCTGTGAAAGGTGACACGCAGGTCAAAGAACAAGGTCTCCTGGACAAAGCAGTTTCAGTAAAAGGGCTCAGTTCTGTGGAAG

G G R Q V K G Q A S I G E A V S V K G L S A R K G H S Q V K G Q D I

GAGGCAGGCAAGTCAAGGGACAAGCCTCCATAGGAGAAGCAGTTTCAGTCAAAGGTCTCAGTGCTAGGAAAGGTCACTCGCAGGTTAAAGGTCAAGATAT

. L Q E A V P V K S V G H V K G G M Q I K G Q D V M G K V V A A K G

TCTACAAGAAGCAGTCCCGGTCAAAAGTGTGGGTCATGTGAAAGGTGGCATGCAGATCAAAGGACAAGATGTCATGGGGAAAGTAGTTGCAGCCAAAGGT

L G P V K S H T Q V K G Q N F Q D A A V S V K T L G S I K R Q G S

CTGGGTCCTGTTAAAAGTCACACGCAGGTCAAAGGACAAAATTTCCAAGATGCAGCAGTTTCAGTTAAAACTCTTGGTTCCATCAAACGTCAGGGTTCTT

S R S Q S S L K G P I Q I K G Q D S T K E A V L V Q G L S S P K S H

CGAGAAGTCAAAGTTCTCTCAAAGGTCCCATACAGATCAAAGGACAAGATTCCACAAAAGAAGCAGTTCTGGTACAAGGTCTAAGTTCTCCAAAAAGTCA

. M Q I H E N A M E D T V L I K G Q D R M K R H R Q F K G Q D H M E.

TATGCAGATTCATGAAAATGCCATGGAAGACACAGTTTTAATCAAAGGTCAAGATCGTATGAAACGTCACCGGCAGTTTAAAGGACAAGATCACATGGAA

.E H A A F K G Q G M F K R R S F S K P G S C P D I T G Q C T Q T S

GAACATGCTGCATTTAAAGGTCAAGGAATGTTCAAAAGGCGAAGTTTCAGTAAACCTGGCTCCTGCCCTGACATTACAGGGCAATGCACACAGACAAGTG

D S K C G S D V E C P G T K K C C V G M C G G M E C L I P E *

ATTCCAAGTGTGGGAGTGATGTTGAGTGCCCAGGGACCAAGAAGTGCTGTGTGGGCATGTGCGGTGGGATGGAGTGTTTGATCCCCGAGTGAGGCAAGCA

CTAGCTGGAAGAAAGGGGGACCCCTGCCAATACAGAAGAAGGTTGAGTGGTGGGGAGTGATGTCAGGGTGGTGGAAAGAAGGAAATGGGGGAGTTGCTGG

TCAGACTGAGAGACTGAGGGGTCTCAGAGGCCATGCGCAAGATGCAGAGGGGATGTCCCTATCTGTGTACTAGGTCTGAGTGCTTTGACCTGCTGAGTCA

TATCTCTGATTCTCTTCTCATCCACCAGGTAAAACCTGTCCTTACTGCACCTGTCCTGGGCCTATGTCTGCAGGCCTTTCTGTAGTCCCTAGTAGCACTT

AGTAGTCTTAGTAGCACTACCCTTTCCCAGTGCCCATTGTTCCTCCTGATCTGGATGCCCAGTCTTGGGACTGCCTTTGTCTTCTACTTTCCAATAAAAA

AGAAAGAAAAAAAAAAAAAAAAA
