# Supplementary material for: Identification of the major rabbit and guinea pig semen coagulum proteins and description of the diversity of the REST gene locus in the mammalian clade Glires
Source: PLoS One. 2020 Oct 14;15(10):e0240607. doi: 10.1371/journal.pone.0240607 (PMC7556508; doi:10.1371/journal.pone.0240607)
Supplement: S2 Fig — The nucleotide sequence of the Svp5 transcript was compared to the guinea pig genome sequence and the trappin transcript by BLAST. Nucleotide differences are highlighted in grey and amino acid substitutions are highlighted in purple. The numbers to the right refers to the position in the Svp5 transcript. (DOCX) [file pone.0240607.s004.docx]

V Q E Q D L L G Q

GTTCAAGAACAGGACCTCCTGGGACAA Svp5 SNP 2,582

GTTCAAGAACAGGGCCTCCTGGGACAA Genomic

V Q E Q G L L G Q

Q V T E R D S M G

CAGGTCACAGAACGAGATTCCATGGGA Svp5 SNP 3,449

CAGGTCACAGAACAAGATTCCATGGGA Genomic

Q V T E Q D S M G

F S K P G S C P D

TTCAGTAAACCTGGCTCCTGCCCTGAC Svp5 Frame shift 4,559

TTCAGTAAACCTG-CTCCTGCCCTGAC Genomic

F S K P -A P A L

K G R M K V K G Q

AAAGGTCGCATGAAGGTCAAAGGACAA Svp5 SNP 350

AAAGGTCGCATGAGGGTCAAAGGACAA Trappin

K G R M R V K G Q

L M G K A V S V K

CTTATGGGAAAAGCAGTTTCTGTCAAA Svp5 SNP 381

CTTATGGGAAAAGCGGTTTCTGTCAAA Trappin

L M G K A V S V K

K G P D S L K G R

AAAGGTCCAGATTCCTTGAAAGGTCGA Svp5 SNP 1,998

AAAGGTCCAGATTCTTTGAAAGGTCGA Trappin

K G P D S L K G R

V S S T K G H I Q

GTCAGTTCTACAAAAGGTCATATACAG Svp5 SNP 3,426

GTCAGTTCTACAAATGGTCATATACAG Trappin

V S S T N G H I Q

S H T Q V K G Q N

AGTCACACGCAGGTCAAAGGACAAAAT Svp5 SNP 4,228

AGTCACACGCAGCTCAAAGAACACAAT Trappin

S H T Q L K E H N

T Q V K G Q N F Q

ACGCAGGTCAAAGGACAAAATTTCCAA Svp5 SNP 4,235

ACGCAGCTCAAAGAACACAATTTCCAA Trappin

T Q L K E H N F Q

Q V K G Q N F Q D

CAGGTCAAAGGACAAAATTTCCAAGAT Svp5 SNP 4,239

CAGCTCAAAGAACACAATTTCCAAGAT Trappin

Q L K E H N F Q D
