# Supplementary material for: Identification of the major rabbit and guinea pig semen coagulum proteins and description of the diversity of the REST gene locus in the mammalian clade Glires
Source: PLoS One. 2020 Oct 14;15(10):e0240607. doi: 10.1371/journal.pone.0240607 (PMC7556508; doi:10.1371/journal.pone.0240607)
Supplement: S3 Fig — The cDNA sequence of SVP200 is shown with the translated protein sequence written above. The predicted signal peptide is highlighted in grey. Peptide sequences highlighted in yellow indicates that they agree in molecular mass with peptides generated by trypsin digestion of the 200 kDa component in the rabbit seminal vesicle secretion. A peptide occurring 3 times in SVP200 is underlined. (DOCX) [file pone.0240607.s005.docx]

M K Y T I F F F S L L L I L E K Q A A G S R H V D I H .

ACGGCTCTTTCTGGCGAGATGAAGTACACCATCTTCTTCTTTTCCCTGCTTCTCATCCTTGAGAAGCAAGCAGCCGGGTCAAGACATGTTGATATTCACA

T L W H L F L K E L E W W Y G E E S G S N E I A S L E T L S P V R T

CACTCTGGCACCTTTTCCTGAAGGAATTGGAGTGGTGGTATGGGGAAGAATCAGGTAGCAATGAAATTGCAAGCTTGGAAACCCTTTCCCCCGTGAGAAC

E E H R K N S E L I H P W S N S I I S V P A E G H I P V Q E Q S Q

AGAAGAGCATCGCAAGAATTCAGAACTCATCCACCCCTGGAGTAACAGCATCATCTCAGTTCCTGCTGAAGGTCACATACCTGTCCAAGAGCAGAGTCAA

I P N K D V P L K H H P L G N K G L G K L P K T S K D H V P R K E

ATACCTAATAAAGATGTTCCACTAAAACATCATCCCCTTGGAAACAAAGGTTTAGGTAAACTTCCAAAAACTAGCAAAGATCATGTTCCAAGGAAGGAGC

Q K S G E S Q D S A E D Q E T D T D H H P V R E N E L S A E L P G K

AAAAGTCTGGTGAAAGTCAAGATTCAGCGGAAGATCAGGAAACTGACACGGATCATCATCCAGTGAGAGAGAATGAGCTGTCTGCAGAGCTTCCAGGCAA

D A G D C E G H V H S K E E D S V E E Q T Q V I D P E T S K E H I

AGATGCGGGAGACTGTGAAGGTCATGTTCATTCAAAAGAGGAAGATTCTGTCGAAGAGCAAACTCAAGTCATAGATCCAGAAACTAGCAAAGAACACATT

L L K E Q Q P L A G Q V P D K D Q E T R K D H F P A N V Q D P V E

CTGTTGAAAGAGCAACAACCTTTGGCTGGACAAGTTCCCGACAAAGATCAAGAAACAAGAAAAGATCATTTTCCTGCAAATGTGCAAGACCCTGTTGAAG

E Q I P V K D Q E A S K D L T P L K Q Q E P S L E H V P A V D Q V T

AGCAAATTCCAGTCAAAGATCAGGAAGCTAGCAAAGATCTCACTCCACTGAAACAGCAAGAGCCTTCCTTAGAACATGTTCCAGCTGTAGATCAGGTAAC

G N D Q D H A K E Q H P V E A Q I P I T S Q E M K R E Q V P L N D.

AGGCAACGATCAAGATCATGCCAAAGAGCAGCATCCTGTTGAAGCACAAATTCCTATAACCAGTCAAGAAATGAAGAGAGAGCAGGTGCCACTGAATGAC

.Q S P V K R Q S P T I S Q G T S K D H I P V R E Q E P A A E I V P .

CAAAGTCCTGTTAAAAGACAAAGTCCAACAATCAGTCAGGGAACTAGCAAAGACCACATTCCAGTGAGAGAGCAGGAGCCTGCTGCAGAAATTGTTCCAG
G K V M E S S K Y P V V A P E Q D A V E G Q S P V K G Q E A S K D F

GAAAAGTTATGGAATCCAGCAAATATCCTGTTGTTGCACCAGAGCAGGATGCTGTTGAAGGACAAAGTCCAGTCAAAGGTCAGGAAGCTAGCAAAGATTT

I P L K G Q E N F E E Q D T T K D Q E P S K H Q D P V K Q Q D P V

CATTCCATTGAAAGGGCAAGAGAATTTTGAAGAACAAGATACAACCAAAGATCAAGAACCAAGCAAACATCAGGATCCAGTGAAACAGCAAGATCCTGTT

E A Q L P E T G H G I G I Q Q V P L K D Q L P V E G Q S P V T G L -

GAAGCACAACTTCCTGAGACTGGTCACGGAATTGGCATACAGCAAGTCCCGTTGAAAGACCAACTTCCAGTTGAAGGACAAAGTCCAGTAACAGGTCTGG

E A S K D H I P V K Q P E P E D G Q V P T N D Q E T S K D H V P M N

AAGCTAGCAAAGACCATATTCCTGTGAAACAGCCAGAGCCTGAAGACGGACAAGTTCCAACCAATGATCAGGAAACTAGCAAAGATCATGTTCCAATGAA

E Q Q P V E Q Q D P A N N Q E T G K D H T P V K E Q D S A K G Q I

TGAGCAACAGCCTGTTGAACAACAAGATCCAGCCAACAATCAGGAAACCGGCAAAGACCACACACCAGTCAAAGAGCAAGACTCTGCTAAAGGGCAGATT

P G K G Q K A S I P S K E Q D T V E E Q V P G T G L E S S K D Q V

CCAGGTAAAGGTCAGAAAGCCAGTATTCCTTCAAAGGAGCAAGATACAGTTGAAGAACAAGTTCCAGGGACTGGTCTAGAAAGCAGCAAAGACCAGGTTC

P V K E Q K P A G G Q V P S K Y Q E A R K D R V L A K E E E S S V G

CAGTGAAGGAACAAAAGCCTGCTGGAGGACAGGTTCCAAGCAAATATCAAGAAGCTCGCAAAGATCGTGTCCTGGCAAAGGAGGAAGAGTCTTCTGTGGG

. Q I P V K D K K S G K D V I P L K D Q E A G E G Q V P A A D Q E T.

ACAAATTCCAGTAAAGGATAAGAAAAGTGGCAAAGATGTGATTCCACTGAAAGACCAAGAGGCTGGTGAAGGACAAGTTCCAGCTGCAGATCAGGAAACT

.S K D H V S E K Q Q Q R V A E Q V P G K Y Q K A S V D H I P A K G .

TCCAAGGATCACGTTTCAGAGAAACAGCAACAGCGTGTTGCAGAACAAGTTCCAGGCAAATATCAGAAAGCCAGTGTAGATCACATTCCTGCAAAGGGGC

Q E S P E G Q A A V S G L E A S K D Q V L M K E Q T P G E E Q V P G

AAGAATCACCTGAAGGACAAGCTGCAGTCTCTGGTCTGGAAGCTAGCAAAGATCAGGTTCTGATGAAAGAGCAAACGCCTGGTGAGGAACAGGTTCCAGG

. K Y P E A S K D H A P A K K Q D S V V G Q I P V K D Q E T G K D V

CAAATATCCAGAAGCTAGTAAAGATCATGCCCCGGCAAAGAAGCAAGATTCTGTTGTAGGACAAATTCCAGTAAAGGATCAGGAAACTGGAAAAGATGTG

I P L K D Q E A G E G Q V P A A D Q E T S K D H V P E K V Q Q R V

ATTCCACTGAAAGACCAAGAGGCTGGTGAAGGACAAGTTCCAGCTGCAGATCAGGAAACCAGCAAAGACCATGTACCAGAGAAAGTGCAACAGCGTGTTG

A E Q L S G K Y Q K A R V D H I P A K E Q E S P E G Q A P A A D Q E

CAGAACAACTTTCAGGCAAATATCAGAAAGCCAGAGTAGATCACATTCCTGCAAAGGAGCAAGAATCACCTGAAGGACAAGCTCCAGCCGCAGATCAGGA

. T S K D H V P E K V Q Q R V A E Q L L G K D Q K A S V D H I P A K

AACCAGCAAAGACCATGTACCAGAGAAAGTGCAACAGCGTGTTGCAGAACAACTTCTAGGCAAAGATCAGAAAGCCAGCGTAGATCACATTCCTGCAAAG

E Q E S P E G Q V P A A D Q E T S K D H V P E K Q Q Q R V E T H L .

GAGCAAGAATCACCTGAAGGACAAGTTCCAGCCGCAGATCAGGAAACCAGCAAAGACCATGTACCAGAGAAACAGCAACAGCGTGTTGAAACACACCTTG

G M T G Q E S T T E Q F P P K D K Y P V Y V Q V P V A S Q E T S K D

GTATGACTGGTCAGGAATCTACAACAGAGCAATTTCCACCGAAAGACAAATATCCTGTTTATGTGCAAGTTCCAGTGGCCAGTCAGGAAACTAGCAAGGA

H V P V K D Q E L G K G Q V P V K D Q K T S K D H V P E K E P Q R

TCATGTTCCAGTGAAAGACCAAGAGCTTGGTAAAGGGCAGGTTCCAGTCAAAGATCAGAAAACCAGCAAAGACCATGTACCAGAGAAAGAGCCACAGCGT

V A E Q L S G K D Q K A S V D H I P A K E Q D S V E G K I A A K G ,

GTTGCAGAACAGCTTTCAGGCAAAGATCAGAAAGCCAGCGTAGATCACATTCCTGCGAAAGAGCAAGATTCTGTTGAAGGAAAAATTGCAGCCAAAGGTC

Q E T S T D H L P A K E Q E P S E A Q A P G T D Q G S S R D L A P S

AGGAAACTAGCACAGATCATCTCCCAGCAAAGGAGCAAGAGCCTTCTGAAGCACAAGCTCCAGGCACAGATCAGGGGAGCAGTAGAGATCTAGCTCCCTC

K P Q E P V V A E A P V S G Q E S A K E Q P P L E D Q D P V H G Q

AAAACCACAAGAACCTGTTGTAGCAGAAGCTCCTGTAAGTGGTCAGGAAAGCGCAAAAGAGCAACCTCCACTGGAAGACCAAGATCCTGTTCATGGACAA

D P A T R Q E E G K V H V P V K D Q D L V L G Q V P G K D Q E T S

GATCCAGCAACCAGGCAGGAAGAAGGCAAAGTTCATGTGCCAGTGAAAGATCAAGATCTCGTTCTAGGACAAGTTCCAGGAAAAGATCAGGAAACTAGCA

K D R V P L T E R R P V E R Q A P A N A Q E P I K D P V K A Q V A A

AAGATCGTGTTCCGTTGACAGAGCGACGCCCTGTTGAGAGACAAGCCCCGGCCAATGCTCAAGAACCTATCAAAGATCCTGTCAAAGCCCAAGTTGCTGC

K H E T S K D H V P V N E Q D P V N E K A S T R S L E T M N D H V.

AAAACATGAAACTAGTAAAGATCATGTTCCAGTGAATGAGCAAGATCCTGTGAATGAAAAAGCTTCAACTAGAAGTCTTGAGACCATGAATGATCACGTT

.P V N V E R D I P G K D Q P T V Q G K V P F R K Q Q L S R G K V P

CCAGTCAATGTTGAAAGAGACATTCCAGGTAAAGATCAACCAACTGTCCAAGGCAAAGTTCCATTCAGAAAACAGCAACTTTCAAGAGGAAAAGTTCCAG

V K C K K L N K G R K T V R S R V P V K A K G S A K G P D T A V Q Q

TCAAATGTAAAAAACTCAACAAAGGTCGCAAAACTGTCAGAAGCCGTGTTCCTGTCAAAGCAAAAGGTAGTGCGAAAGGTCCAGATACTGCCGTACAGCA

. V K Q I A G T T P *

GGTGAAACAGATTGCTGGGACGACACCATAACCACCATCCCTAGGAAAGCCACTTAGAGTCCTGGCTGCTCTATACCAGCTCTGCTAACACAGCGAGGAA

GCAGACGATGGCTTGAGTGGCTGGGCGCCTGGCACTCACAAGGGAGACCCAGATGCAGTTCCAGGCTCCTGGCTTCAACCTGGCCCAGCCCTGCTGTTGT

AGCTATTTGGGAGAGTAAACTAGTGGCTGAAAGATCTCTCTCTCCTTTCTCTCTCTCTCTCTCTCTCTCTCTTTACCCCTCCCTCGTTACCTCCCTCTCC

CTCTTCCTCTCTCTCTCTCTGCCCCTCATATATATAAATAAATATATATTTATAAAAAAAAAAAAAAAAAAAAAAAAAAAAAAAA
