# Supplementary material for: Identification of the major rabbit and guinea pig semen coagulum proteins and description of the diversity of the REST gene locus in the mammalian clade Glires
Source: PLoS One. 2020 Oct 14;15(10):e0240607. doi: 10.1371/journal.pone.0240607 (PMC7556508; doi:10.1371/journal.pone.0240607)
Supplement: S4 Fig — The SVP200 transcript from the lop eared rabbit is given with non-translated nucleotides highlighted in grey. A block of 144 nucleotides, not present in the genome database, is highlighted in blue and an adjoining, almost identical, sequence is highlighted in yellow. A CT dinucleotide in the microsatellite, located in the 3’ non-translated sequence, which is not present in the genome sequence, is also highlighted in blue. Nucleotide positions in the SVP200 transcript that differ from those in the rabbit genome database are highlighted purple. (DOCX) [file pone.0240607.s006.docx]

>Lop_rabbit_SVP200

ACGGCTCTTTCTGGCGAGATGAAGTACACCATCTTCTTCTTTTCCCTGCTTCTCATCCTTGAGAAGCAAGCAGCCGGGTCAAGACATGTTGATATTCACACACTCTGGCACCTTTTCCTGAAGGAATTGGAGTGGTGGTATGGGGAAGAATCAGGTAGCAATGAAATTGCAAGCTTGGAAACCCTTTCCCCCGTGAGAACAGAAGAGCATCGCAAGAATTCAGAACTCATCCACCCCTGGAGTAACAGCATCATCTCAGTTCCTGCTGAAGGTCACATACCTGTCCAAGAGCAGAGTCAAATACCTAATAAAGATGTTCCACTAAAACATCATCCCCTTGGAAACAAAGGTTTAGGTAAACTTCCAAAAACTAGCAAAGATCATGTTCCAAGGAAGGAGCAAAAGTCTGGTGAAAGTCAAGATTCAGCGGAAGATCAGGAAACTGACACGGATCATCATCCAGTGAGAGAGAATGAGCTGTCTGCAGAGCTTCCAGGCAAAGATGCGGGAGACTGTGAAGGTCATGTTCATTCAAAAGAGGAAGATTCTGTCGAAGAGCAAACTCAAGTCATAGATCCAGAAACTAGCAAAGAACACATTCTGTTGAAAGAGCAACAACCTTTGGCTGGACAAGTTCCCGACAAAGATCAAGAAACAAGAAAAGATCATTTTCCTGCAAATGTGCAAGACCCTGTTGAAGAGCAAATTCCAGTCAAAGATCAGGAAGCTAGCAAAGATCTCACTCCACTGAAACAGCAAGAGCCTTCCTTAGAACATGTTCCAGCTGTAGATCAGGTAACAGGCAACGATCAAGATCATGCCAAAGAGCAGCATCCTGTTGAAGCACAAATTCCTATAACCAGTCAAGAAATGAAGAGAGAGCAGGTGCCACTGAATGACCAAAGTCCTGTTAAAAGACAAAGTCCAACAATCAGTCAGGGAACTAGCAAAGACCACATTCCAGTGAGAGAGCAGGAGCCTGCTGCAGAAATTGTTCCAGGAAAAGTTATGGAATCCAGCAAATATCCTGTTGTTGCACCAGAGCAGGATGCTGTTGAAGGACAAAGTCCAGTCAAAGGTCAGGAAGCTAGCAAAGATTTCATTCCATTGAAAGGGCAAGAGAATTTTGAAGAACAAGATACAACCAAAGATCAAGAACCAAGCAAACATCAGGATCCAGTGAAACAGCAAGATCCTGTTGAAGCACAACTTCCTGAGACTGGTCACGGAATTGGCATACAGCAAGTCCCGTTGAAAGACCAACTTCCAGTTGAAGGACAAAGTCCAGTAACAGGTCTGGAAGCTAGCAAAGACCATATTCCTGTGAAACAGCCAGAGCCTGAAGACGGACAAGTTCCAACCAATGATCAGGAAACTAGCAAAGATCATGTTCCAATGAATGAGCAACAGCCTGTTGAACAACAAGATCCAGCCAACAATCAGGAAACCGGCAAAGACCACACACCAGTCAAAGAGCAAGACTCTGCTAAAGGGCAGATTCCAGGTAAAGGTCAGAAAGCCAGTATTCCTTCAAAGGAGCAAGATACAGTTGAAGAACAAGTTCCAGGGACTGGTCTAGAAAGCAGCAAAGACCAGGTTCCAGTGAAGGAACAAAAGCCTGCTGGAGGACAGGTTCCAAGCAAATATCAAGAAGCTCGCAAAGATCGTGTCCTGGCAAAGGAGGAAGAGTCTTCTGTGGGACAAATTCCAGTAAAGGATAAGAAAAGTGGCAAAGATGTGATTCCACTGAAAGACCAAGAGGCTGGTGAAGGACAAGTTCCAGCTGCAGATCAGGAAACTTCCAAGGATCACGTTTCAGAGAAACAGCAACAGCGTGTTGCAGAACAAGTTCCAGGCAAATATCAGAAAGCCAGTGTAGATCACATTCCTGCAAAGGGGCAAGAATCACCTGAAGGACAAGCTGCAGTCTCTGGTCTGGAAGCTAGCAAAGATCAGGTTCTGATGAAAGAGCAAACGCCTGGTGAGGAACAGGTTCCAGGCAAATATCCAGAAGCTAGTAAAGATCATGCCCCGGCAAAGAAGCAAGATTCTGTTGTAGGACAAATTCCAGTAAAGGATCAGGAAACTGGAAAAGATGTGATTCCACTGAAAGACCAAGAGGCTGGTGAAGGACAAGTTCCAGCTGCAGATCAGGAAACCAGCAAAGACCATGTACCAGAGAAAGTGCAACAGCGTGTTGCAGAACAACTTTCAGGCAAATATCAGAAAGCCAGAGTAGATCACATTCCTGCAAAGGAGCAAGAATCACCTGAAGGACAAGCTCCAGCCGCAGATCAGGAAACCAGCAAAGACCATGTACCAGAGAAAGTGCAACAGCGTGTTGCAGAACAACTTCTAGGCAAAGATCAGAAAGCCAGCGTAGATCACATTCCTGCAAAGGAGCAAGAATCACCTGAAGGACAAGTTCCAGCCGCAGATCAGGAAACCAGCAAAGACCATGTACCAGAGAAACAGCAACAGCGTGTTGAAACACACCTTGGTATGACTGGTCAGGAATCTACAACAGAGCAATTTCCACCGAAAGACAAATATCCTGTTTATGTGCAAGTTCCAGTGGCCAGTCAGGAAACTAGCAAGGATCATGTTCCAGTGAAAGACCAAGAGCTTGGTAAAGGGCAGGTTCCAGTCAAAGATCAGAAAACCAGCAAAGACCATGTACCAGAGAAAGAGCCACAGCGTGTTGCAGAACAGCTTTCAGGCAAAGATCAGAAAGCCAGCGTAGATCACATTCCTGCGAAAGAGCAAGATTCTGTTGAAGGAAAAATTGCAGCCAAAGGTCAGGAAACTAGCACAGATCATCTCCCAGCAAAGGAGCAAGAGCCTTCTGAAGCACAAGCTCCAGGCACAGATCAGGGGAGCAGTAGAGATCTAGCTCCCTCAAAACCACAAGAACCTGTTGTAGCAGAAGCTCCTGTAAGTGGTCAGGAAAGCGCAAAAGAGCAACCTCCACTGGAAGACCAAGATCCTGTTCATGGACAAGATCCAGCAACCAGGCAGGAAGAAGGCAAAGTTCATGTGCCAGTGAAAGATCAAGATCTCGTTCTAGGACAAGTTCCAGGAAAAGATCAGGAAACTAGCAAAGATCGTGTTCCGTTGACAGAGCGACGCCCTGTTGAGAGACAAGCCCCGGCCAATGCTCAAGAACCTATCAAAGATCCTGTCAAAGCCCAAGTTGCTGCAAAACATGAAACTAGTAAAGATCATGTTCCAGTGAATGAGCAAGATCCTGTGAATGAAAAAGCTTCAACTAGAAGTCTTGAGACCATGAATGATCACGTTCCAGTCAATGTTGAAAGAGACATTCCAGGTAAAGATCAACCAACTGTCCAAGGCAAAGTTCCATTCAGAAAACAGCAACTTTCAAGAGGAAAAGTTCCAGTCAAATGTAAAAAACTCAACAAAGGTCGCAAAACTGTCAGAAGCCGTGTTCCTGTCAAAGCAAAAGGTAGTGCGAAAGGTCCAGATACTGCCGTACAGCAGGTGAAACAGATTGCTGGGACGACACCATAACCACCATCCCTAGGAAAGCCACTTAGAGTCCTGGCTGCTCTATACCAGCTCTCTGCTAACACAGCGAGGAAGCAGACGATGGCTTGAGTGGCTGGGCGCCTGGCACTCACAAGGGAGACCCAGATGCAGTTCCAGGCTCCTGGCTTCAACCTGGCCCAGCCCTGCTGTTGTAGCTATTTGGGAGAGTAAACTAGTGGCTGAAAGATCTCTCTCTCCTTTCTCTCTCTCTCTCTCTCTCTCTCTTTACCCCTCCCTCGTTACCTCCCTCTCCCTCTTCCTCTCTCTCTCTCTGCCCCTCATATATATAAATAAATATATATTTATAAAAAA
