# Supplementary material for: Identification of the major rabbit and guinea pig semen coagulum proteins and description of the diversity of the REST gene locus in the mammalian clade Glires
Source: PLoS One. 2020 Oct 14;15(10):e0240607. doi: 10.1371/journal.pone.0240607 (PMC7556508; doi:10.1371/journal.pone.0240607)
Supplement: S5 Fig — The aligned sequences are shown with vertical bars indicating conserved nucleotides. The sequences highlighted in grey are 3’ non-translated nucleotides and those highlighted in green are translated nucleotides in exon 2 of PI3, with the translation written below. The stop codon is highlighted in red and the polyadenylation signals are underlined. (DOCX) [file pone.0240607.s007.docx]

*SVP200* CTCTCTCTGCCCCTCAAATATATAAATAAATAAATAT------TTTTAAA---------- 6811

| | ||||||| | ||||||

*PI3* TGAAGCAGAGGCTTACTGGGTATAAATGTGGGCTCGTTTCTTCTTTTAACAGTTCCTGTT 968

V P V

*SVP200* AAATGTTCTGAGACTGTCAAAGATCATGTTTTGGTTAAAGGAAAA-ATGCTCCTAAAGAT 6870

||| || || |||||||||| | |||| | || ||| || || | |||||

*PI3* AAAGGTCAAGACACTGTCAAAGGCCGTGTTCCATTCAATGGACAAGATCCCGTTAAAGGA 1028

K G Q D T V K G R V P F N G Q D P V K G

*SVP200* CAATCAACAATTAA-GACCAATATATAGCTCCATGC-CAAGATT---TCAATG------- 6918

||| || |||| | ||| ||| || || | || ||||| |||| |

*PI3* CAAGTTTCAGTTAAAGGTCAAGATAAAG-TCAAAGCGCAAGAGCCAGTCAAAGGTCCAGT 1087

Q V S V K G Q D K V K A Q E P V K G P V

*SVP200* --CCAATAAGTCTAAAGCCTCCCTCAAT-TGTCCATCCTGTGGCAGGCATG--GGCTTGC 6973

||| |||| || ||| || || | | | |||| ||| | |||| | | |

*PI3* CTCCACTAAGCCTGGCTCCTGCCCCATTATCTTGATCCGGTGC--GCCATGTTGAATCCC 1145

S T K P G S C P I I L I R C A M L N P

*SVP200* ATTATAAGC--CATAAGACATGATGATTGCCCATTGGATGA-GTTCTGCAGTGTAAGCAG 7030

|| || | | | | || ||| |||||| | || | | ||| ||| | ||

*PI3* CCTAACCGCTGCTTGAAAGATACTGACTGCCCAG-GAATCAAGAAGTGCTGTGAAGGCTC 1204

P N R C L K D T D C P G I K K C C E G S

*SVP200* TTGTAGGAATCTCTATAGGAACCACCCATTAGGTGAACCCAAGAACACAACCTGGAAGAA 7090

||| ||| || | | || | |||||| | | || ||||| |||

*PI3* TTGCGGGATGGCCTGTTTCGTTCCCCAGTGAGGTGAGCACTAG--------CTGGA-GAA 1255

C G M A C F V P Q *

*SVP200* TAAGG-GATCCCTGAGGACACAGAAGAAGGCTGAGCAG-GAAGATTTAATGCAAGAGGTT 7148

||| || |||||| |||||| ||||||||||||| | | || | || | | |

*PI3* CGAGGAGACCCCTGAAGACACAAAAGAAGGCTGAGCGGTGGGGAAGCATCCCAGGTTGGT 1315

*SVP200* GGTAAGGAGGCTATGGAGAGGTGAGAGACAGA------GGTTGAGGGCTTCCAGAGGCCA 7202

|| | ||||| | ||| ||||||| ||| ||| | |||||| | |||||| |

*PI3* GGGAGGGAGGTTGTGG-GAGGTGACAGAAAGACTGGGAGACTGAGGGGTCTGAGAGGCTA 1374

*SVP200* TAACTACAGTGTAGAGAGGAACGATCCTGCCTCCTCACTGCCTCTGAGTGCTTGGACCTG 7262

|||| | |||| ||| | | |||| | |||||||||||||||||||||| || ||

*PI3* TAACCAGAGTGCCTAGAAGGATGATCTGTCTTCCTCACTGCCTCTGAGTGCTTTGATGTG 1434

*SVP200* CGGAGTTACACCTA--ATTCTCCTTTCTCCACCAGCTGGAACCTGTCCCCACTGCACCTG 7320

| || | ||||| || ||| | ||| | ||| ||| || |||| |||||||||

*PI3* CTGACTCTCACCTCTGATACTCTTCTCTTCCACAGAGGGAGCCGGTCCTTGCTGCACCTG 1494

*SVP200* TGATG--CCAAGAGATATT---CCCAGTTC--CCTATAGT----GCTCCCCT--CTCTTC 7367

|| | || |||| || |||| | |||| ||| ||| |||| | ||||

*PI3* TGCCGTCCCCAGAGCTACAGGCCCCATCTGGTCCTA-AGTCCCTGCTGCCCTTCCCCTTC 1553

*SVP200* TCT-AC---CCATTCTTCCTTGCATTTAGGATGCCCAAGCCTGGGGCTGCTTCTCTCATC 7423

| || ||||||||||| |||| |||||||||| | |||| ||||| |||||||||

*PI3* CCACACTGTCCATTCTTCCTCCCATTCAGGATGCCCACGGCTGGAGCTGCCTCTCTCATC 1613

*SVP200* CACTTTCCAATAAAAAGTCTCCTTTCTGCTC-ATTTATTTCTGGCTCCTGTGATTTCTGG 7482

|||||||||||||| ||| ||||| |||||| | || ||||||| |||| ||| ||||||

*PI3* CACTTTCCAATAAAGAGT-TCCTT-CTGCTCCACTTGTTTCTGGTTCCTATGACTTCTGG 1671

*SVP200* GTTCCACAAAACTCTGGGGAGATGGGTGTGGAAGTGGAA-TCCCTCTCACCACTGGGGAG 7541

| ||| | || |||||| |||| ||| ||| ||| | | | |||| ||| || |||

*PI3* GCTCCTGGATGCTTTGGGGAAATGGATGTAGAATTGGGACTTCTTCTCTCCAGTGAAGAG 1731
