# Supplementary figures and images for: Identification of the major rabbit and guinea pig semen coagulum proteins and description of the diversity of the REST gene locus in the mammalian clade Glires
Source: PLoS One. 2020 Oct 14;15(10):e0240607. doi: 10.1371/journal.pone.0240607 (PMC7556508; doi:10.1371/journal.pone.0240607)

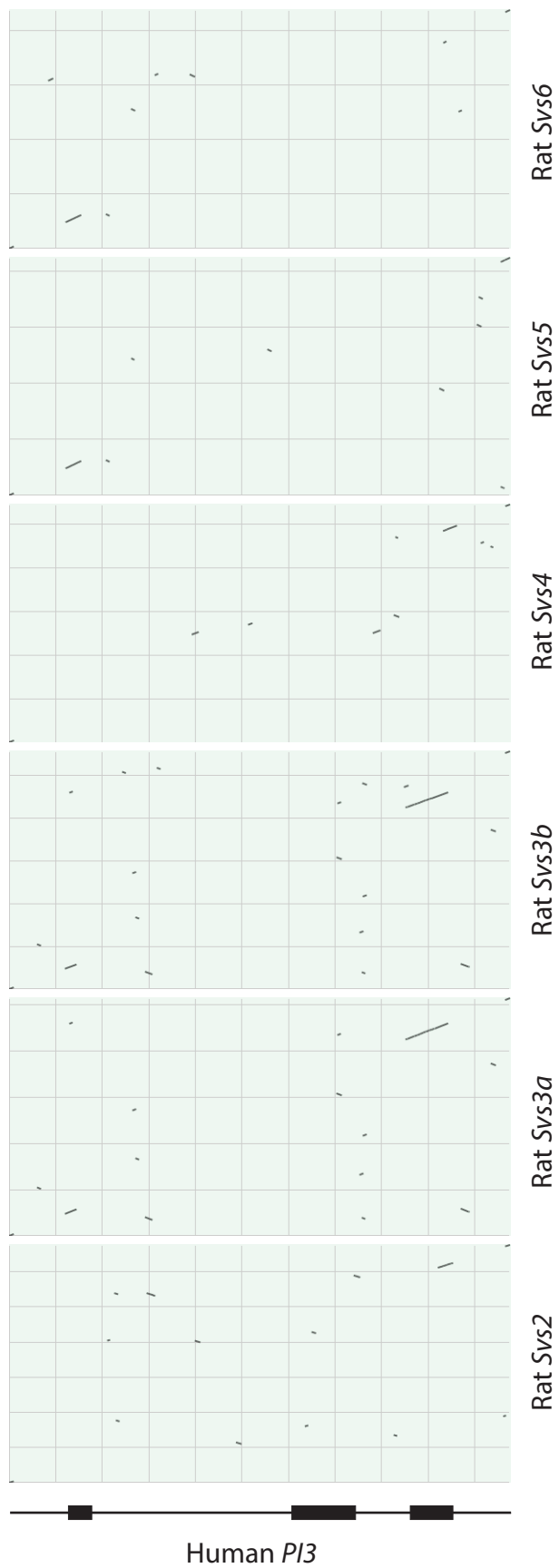

Supplement: S6 Fig — Sequences were aligned using the computer program BLAST, which also generated dotplots that illustrated the location of conserved nucleotides. The compared sequences consisted of the genes and 200 bp of 5’ and 3’ gene flanking DNA. Human PI3 is illustrated as a horizontal line with boxes showing, from left to right, the location of exons 1–3. (PDF) [file pone.0240607.s008.pdf]

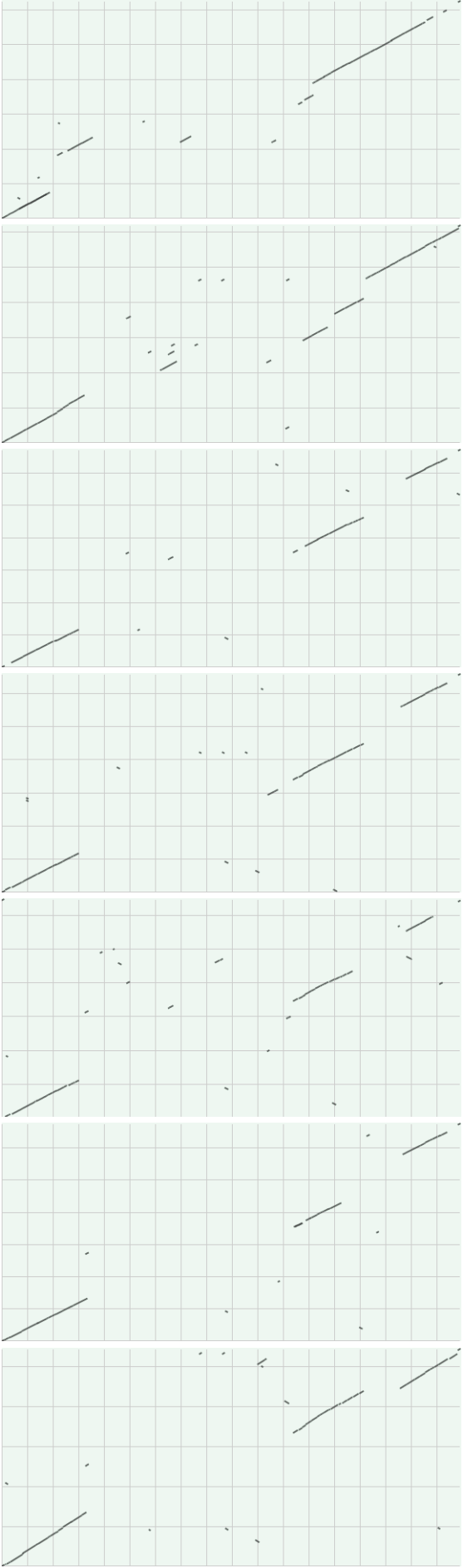

Human *SEMG2*

Supplement: S8 Fig — The dotplos were generated by the sequence alignment program BLAST. Compared sequences consisted of genes and 200 bp flanking DNA at both ends. Below the dotplots is a schematic illustration of SEMG2, with boxes showing, from left to right, the approximate location of exons 1–3, i.e. SPCE, MCE, and 3NTE. (PDF) [file pone.0240607.s010.pdf]

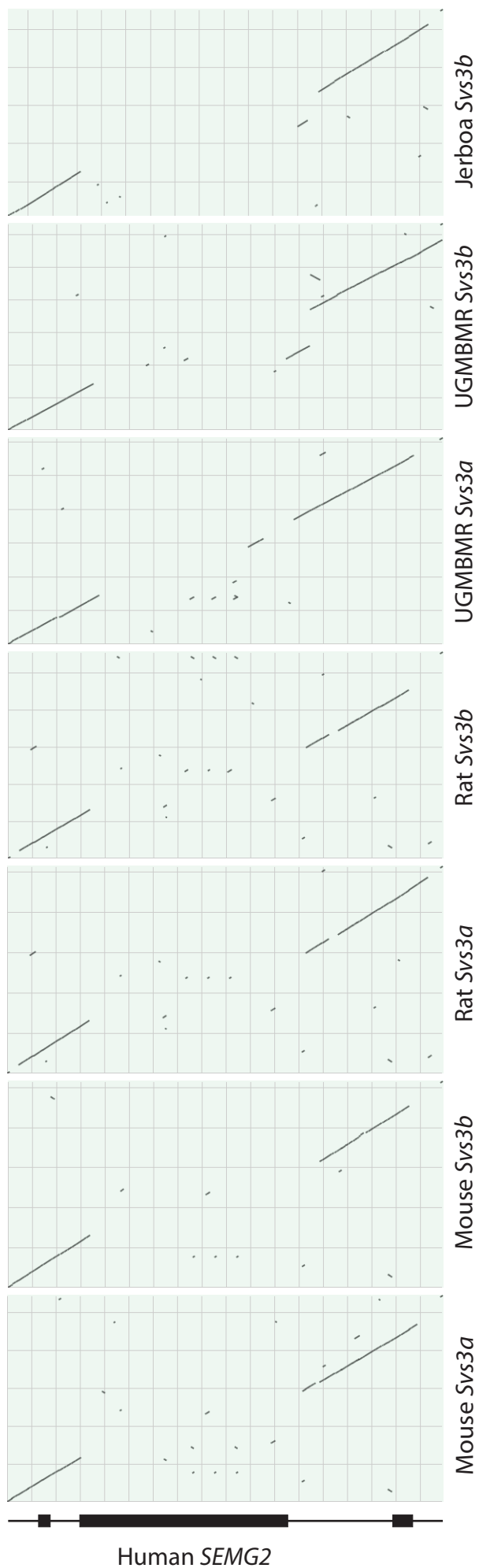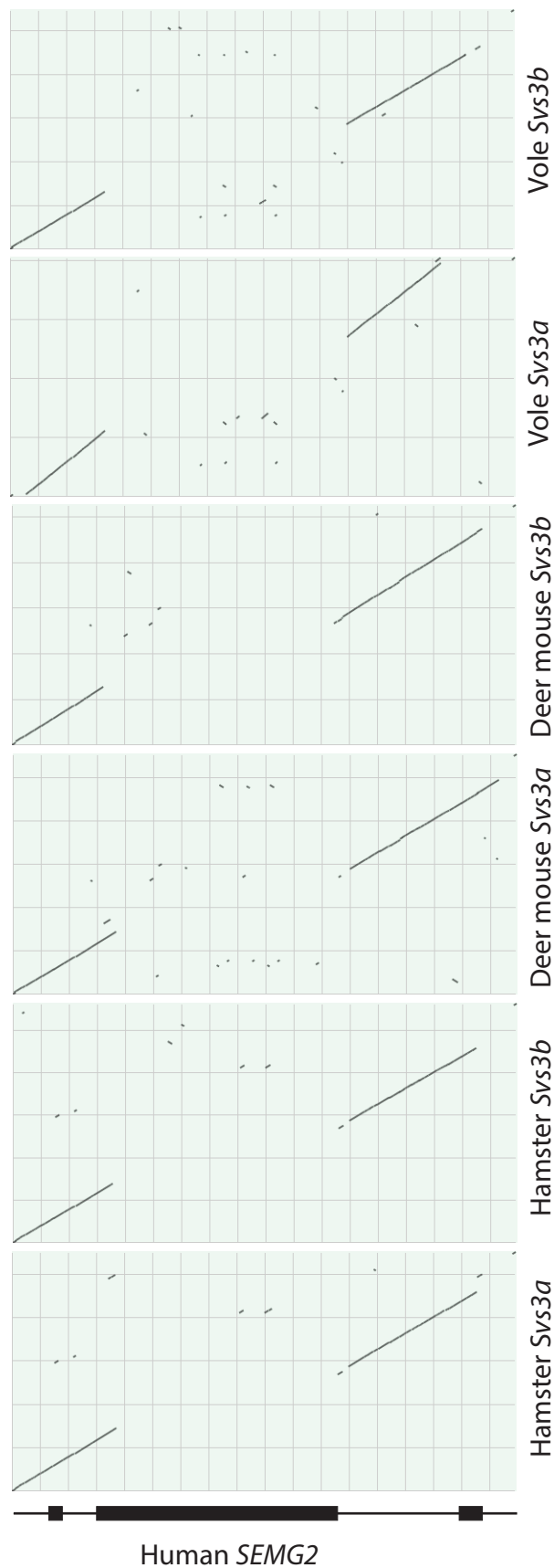

Supplement: S9 Fig — The dotplos were generated by the sequence alignment program BLAST. Compared sequences consisted of genes and 200 bp flanking DNA at both ends. Below the dotplots is a schematic illustration of SEMG2, with boxes showing, from left to right, the approximate location of exons 1–3, i.e. SPCE, MCE, and 3NTE. (PDF) [file pone.0240607.s011.pdf]

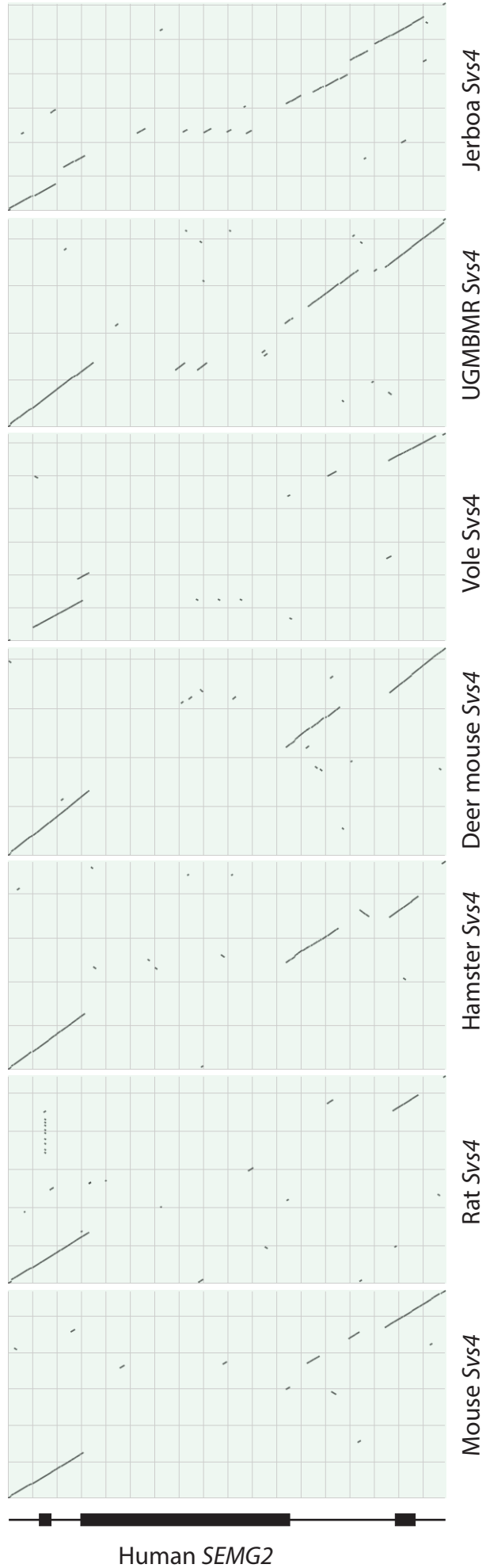

Supplement: S10 Fig — The dotplos were generated by the sequence alignment program BLAST. Compared sequences consisted of genes and 200 bp flanking DNA at both ends. Below the dotplots is a schematic illustration of SEMG2, with boxes showing, from left to right, the approximate location of exons 1–3, i.e. SPCE, MCE, and 3NTE. (PDF) [file pone.0240607.s012.pdf]

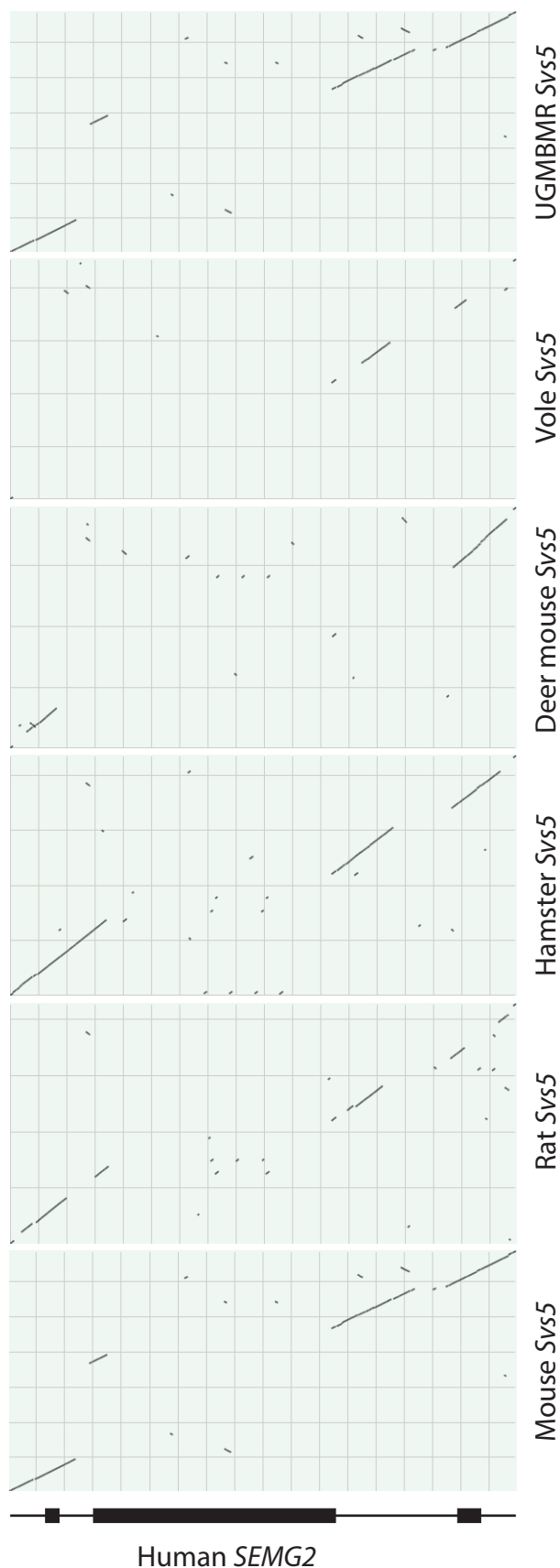

Supplement: S11 Fig — The dotplos were generated by the sequence alignment program BLAST. Compared sequences consisted of genes and 200 bp flanking DNA at both ends. Below the dotplots is a schematic illustration of SEMG2, with boxes showing, from left to right, the approximate location of exons 1–3, i.e. SPCE, MCE, and 3NTE. (PDF) [file pone.0240607.s013.pdf]

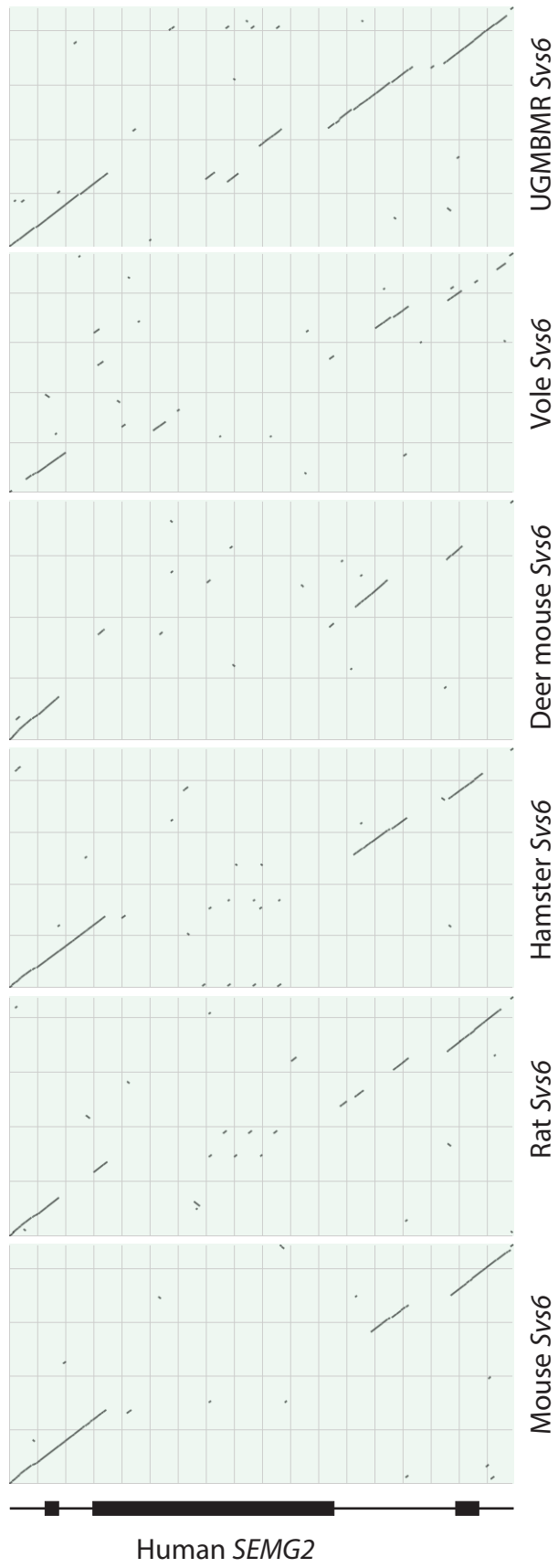

Supplement: S12 Fig — The dotplos were generated by the sequence alignment program BLAST. Compared sequences consisted of genes and 200 bp flanking DNA at both ends. Below the dotplots is a schematic illustration of SEMG2, with boxes showing, from left to right, the approximate location of exons 1–3, i.e. SPCE, MCE, and 3NTE. (PDF) [file pone.0240607.s014.pdf]

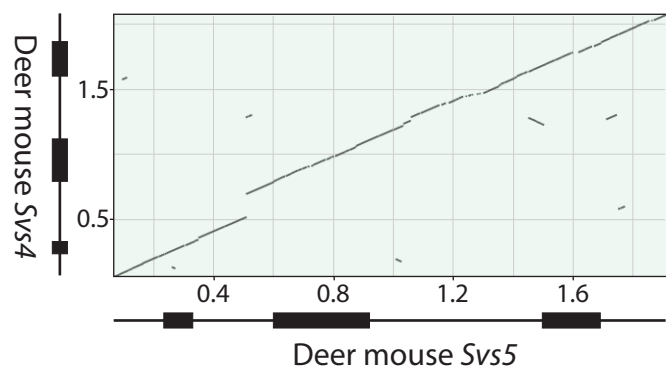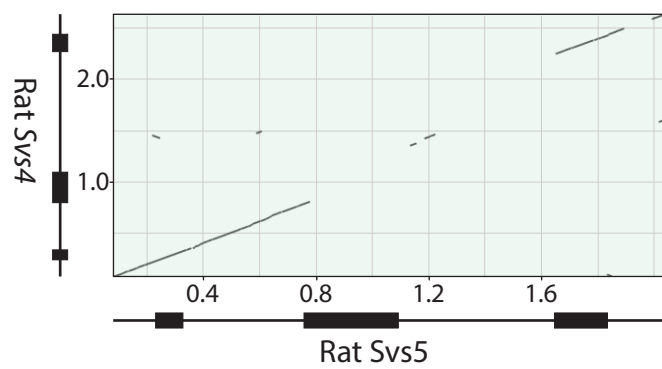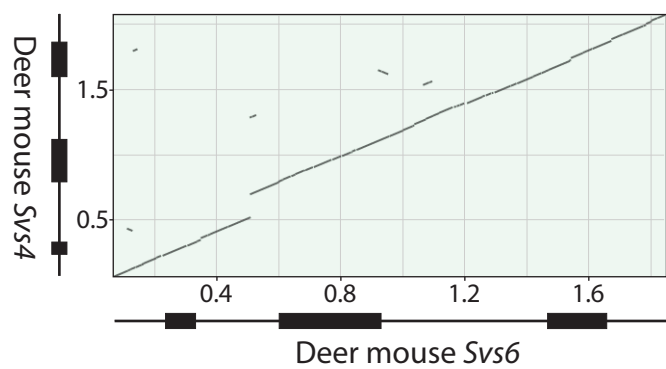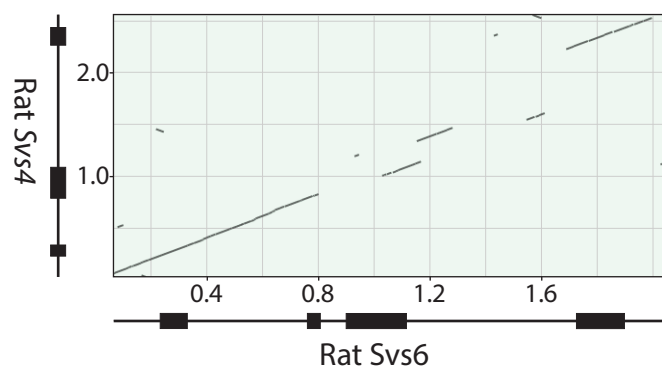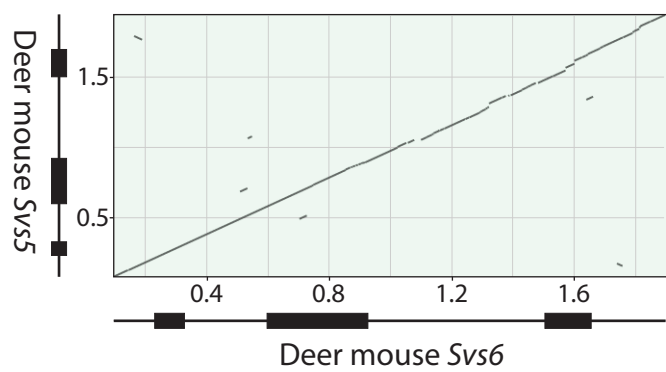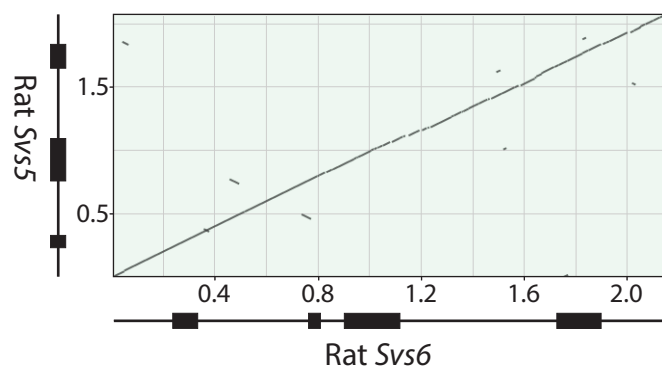

Supplement: S18 Fig — Dotplots generated by BLAST alignment of Svs4, Svs5, and Svs6 from deer mouse and rat. The illustrations below and beside the axis depict the genes, with rectangles indicating the location of exons. (PDF) [file pone.0240607.s020.pdf]

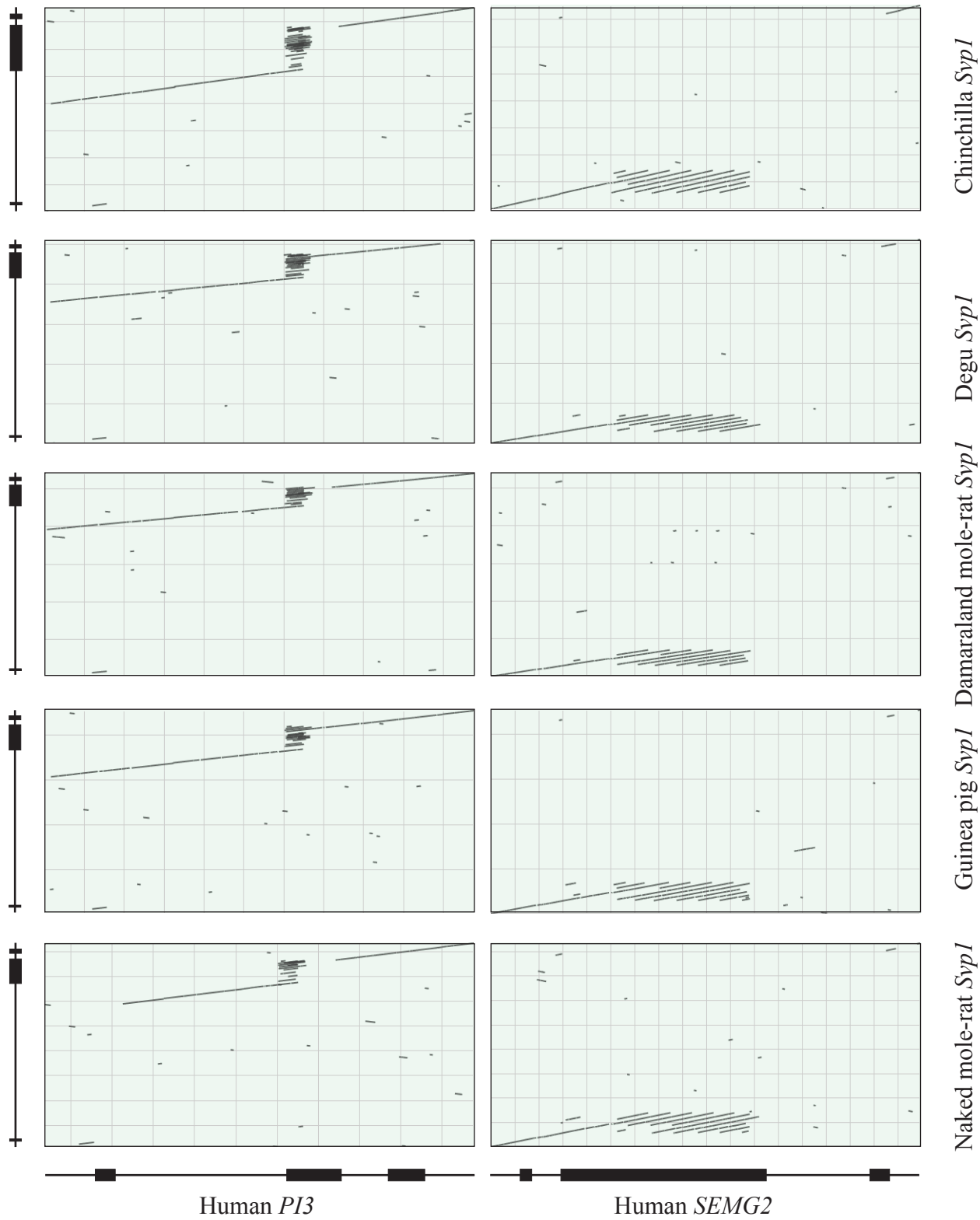

Supplement: S24 Fig — Dotplots were generated by the alignment program BLAST. The genes are outlined below and beside the dotplots, with location of exons illustrated by boxes. (PDF) [file pone.0240607.s026.pdf]

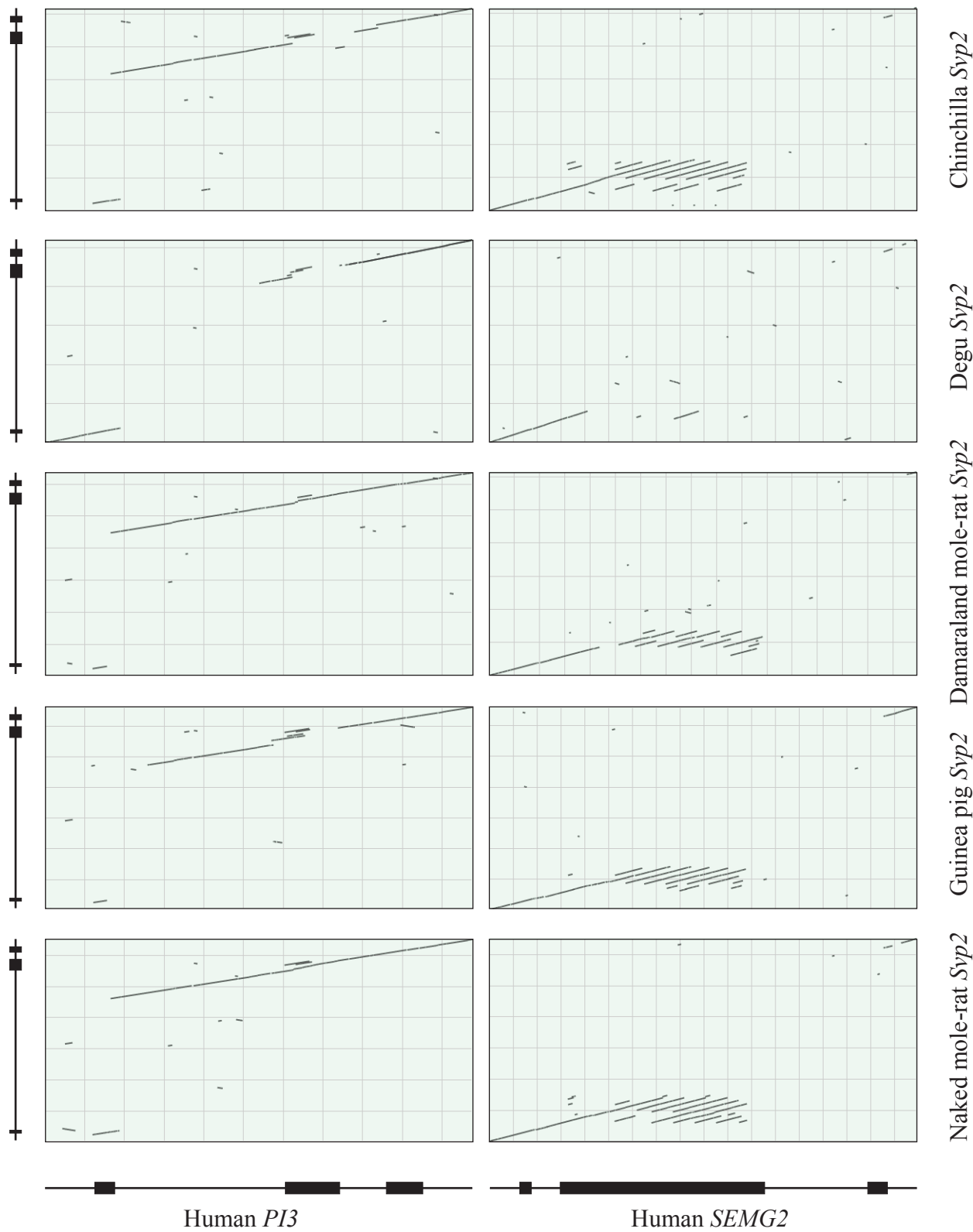

Supplement: S25 Fig — Dotplots were generated by the alignment program BLAST. The genes are outlined below and beside the dotplots, with location of exons illustrated by boxes. (PDF) [file pone.0240607.s027.pdf]

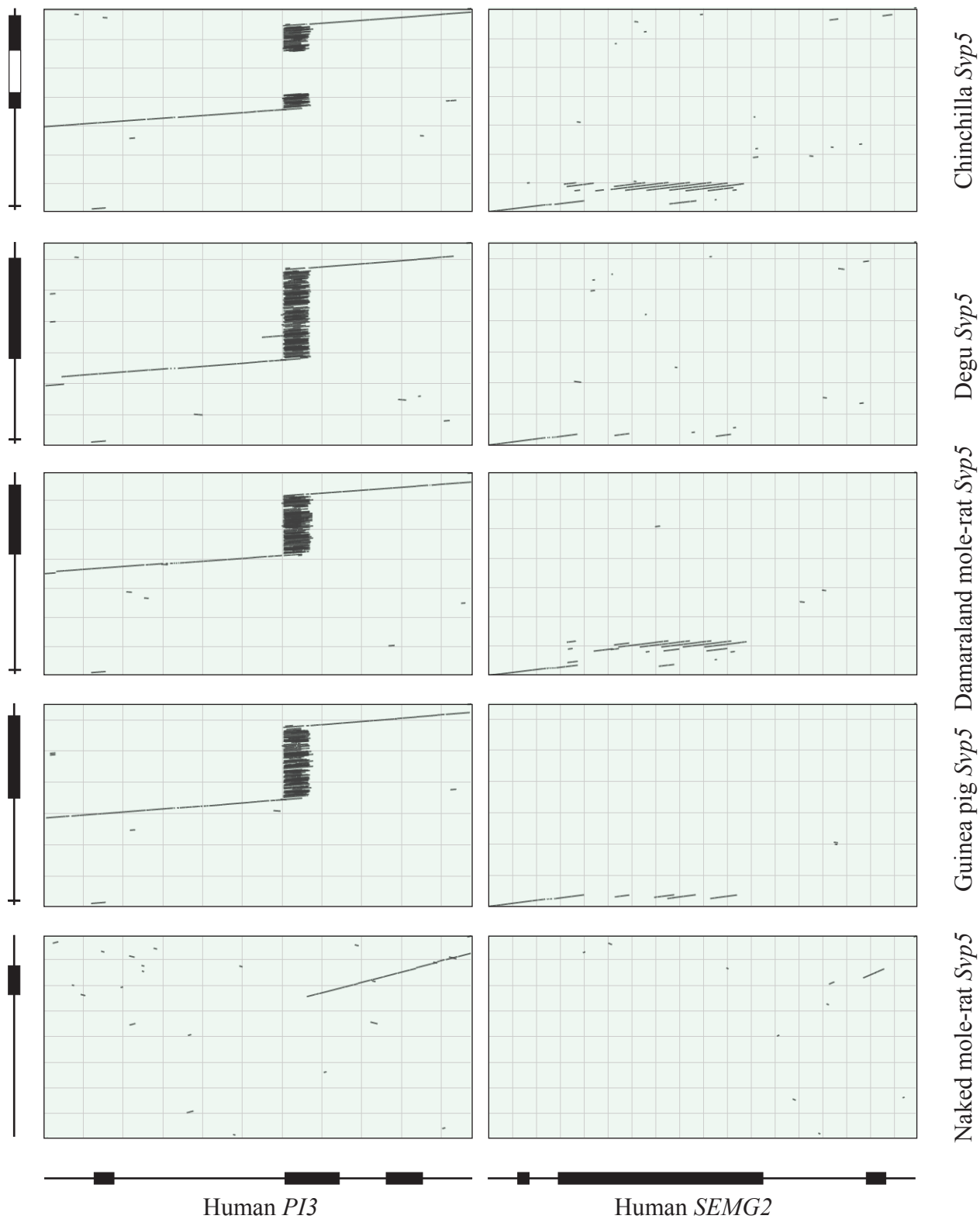

Supplement: S26 Fig — Dotplots were generated by the alignment program BLAST. The genes are outlined below and beside the dotplots, with location of exons illustrated by boxes. (PDF) [file pone.0240607.s028.pdf]
