# Supplementary material for: Identification of the major rabbit and guinea pig semen coagulum proteins and description of the diversity of the REST gene locus in the mammalian clade Glires
Source: PLoS One. 2020 Oct 14;15(10):e0240607. doi: 10.1371/journal.pone.0240607 (PMC7556508; doi:10.1371/journal.pone.0240607)
Supplement: S7 Fig — The aligned sequences are shown with conserved nucleotides indicated by vertical bars. Exon sequences are highlighted in green if they are translated or in grey if they are non-translated. (DOCX) [file pone.0240607.s009.docx]

**Rat Svs2 exon 3**

Human PI3 1844 CTGGAGCTGCCTCTCTCATCCA-CTTTCCAATAAAGAGTT--CCTTCTGCTCCACTTGTTTCTGGTTC 1908

|||||||| || | | | |||||||||||| | | | |||||| |||| | ||| ||||

Rat Svs2 3037 CTGGAGCTTCCCCCAGACACTAGCTTTCCAATAAAAAAATAACTTTCTGCATCACTCG-CTCTTGTTC 3103

**Rat Svs3a exon 1**

Human PI3 241 CTTAGCTCTTAGCCAAACACCTTCCTGACACCATGAGGGCCAGCAGCTTC 290

|| |||||| || | |||||||| | |||| | ||| | ||||

Rat Svs3a 219 CTGAGCTCTCAGTGGAGGCCCTTCCTGGTAAGATGAAGTCCATCTTCTTC 268

**Rat *Svs3a* exon 3**

Human *PI3* 1705 TCTTCTCTTCCACAGAGGGAGCCGGTCCTTGCTGCACCTGTGCCGTCCCCAGAGCTACAGGCC-CCATCTGGTCCT-AAGTCCCTGCTGCCCTTCCCCTTCCCACACTGTCCATTCTTCC 1822

|| ||||| ||| || | || | ||| || || ||||| || | | || | | | | || | ||||||| || || ||| | | || | || |||| | | ||||

Rat *Svs3a* 2107 TCCTCTCT-CCATAGGAGTCACCTGACCTGTGTGGAC-TGTGCGGTTCTTA-AGATGCTGACTGCCGTGTGGTCCTCAACACCTTGCA-CGGATGCCATGACC-CACTCAC-ACA-TTCC 2219

Human *PI3* 1823 TCCCATTCAGGATGCCCACGGCTGGAGCTGCCTCTCTCATCCACTTTCCAATAAAGAGTTCCTTC 1887

| ||| || | | | | |||||| |||| | || | ||||||||| || |||||

Rat *Svs3a* 2220 TTCCAG--AGACTTTCTAAAGTTGGAGCAACCTCACGCAGGTGCCCTCCAATAAACAGAGCCTTC 2282

**Rat *Svs3b* exon 1**

Human *PI3* 241 CTTAGCTCTTAGCCAAACACCTTCCTGACACCATGAGGGCCAGCAGCTTC 290

|| |||||| || | |||||||| | |||| | ||| | ||||

Rat *Svs3b* 219 CTGAGCTCTCAGTGGAGGCCCTTCCTGGTAAGATGAAGTCCATCTTCTTC 268

**Rat *Svs3b* exon 3**

Human *PI3* 1705 TCTTCTCTTCCACAGAGGGAGCCGGTCCTTGCTGCACCTGTGCCGTCCCCAGAGCTACAGGCC-CCATCTGGTCCT-AAGTCCCTGCTGCCCTTCCCCTTCCCACACTGTCCATTCTTCC 1822

|| ||||| ||| || | || | ||| || || ||||| || | | || | | | | || | ||||||| || || ||| | | || | || |||| | | ||||

Rat *Svs3b* 2106 TCCTCTCT-CCATAGGAGTCACCTGACCTGTGTGGAC-TGTGCGGTTCTTA-AGATGCTGACTGCCCTGTGGTCCTCAACACCTTGCA-CGGATGCCATGACC-CACTCAC-ACA-TTCC 2218

Human *PI3* 1823 TCCCATTCAGGATGCCCACGGCTGGAGCTGCCTCTCTCATCCACTTTCCAATAAAGAGTTCCTTC 1887

| ||| || | | | | |||||| | || | || | ||||||||| || |||||

Rat *Svs3b* 2219 TTCCAGAGAGACTTTCTAAAGTTGGAGCAACTTCACGCAGGTGCCCTCCAATAAACAGAGCCTTC 2283

**Rat *Svs4* exon3**

Human *PI3* 1866 CTTTCCAATAAAGAGTT--CCTTCTGCTCCACTTGTTTCTGGTTCCTATGACTTCTGGGCTC 1925

|| |||||||||||| | | ||||| || | || || ||| ||||||| |||

Rat *Svs4* 2398 CTGTCCAATAAAGAGATAACAATCTGCATCATTAACTTTTGTCTCCCGAGACTTCTTGGCTC 2459

**Rat *Svs5* exon 1**

Human *PI3* 244 AGCTCTTAGCCAAACACCTTCCTGACACCATGAGGGCCAGCAGCTTCTTGATCGTGGTGGTGTTCCT 310

|||||| || ||| ||||||||||| ||||| ||| | | ||||| || | |||| ||||

Rat *Svs5* 222 AGCTCTCAGTCAAGACCCTTCCTGACAAGATGAGTCCCACCGGGTTCTTCCTCCTTACGGTGCTCCT 288

**Rat *Svs6* exon 1**

Human *PI3* 244 AGCTCTTAGCCAAACACCTTCCTGACACCATGAGGGCCAGCAGCTTCTTGATCGTGGTGGTGTTCCT 310

|||||| || ||| ||||||||||| ||||| ||| ||| ||||| || | |||| ||||

Rat *Svs6* 222 AGCTCTCAGTCAAGACCCTTCCTGACAAGATGAGTCCCACCAGGTTCTTCCTCCTTACGGTGCTCCT 288
