# Supplementary material for: Identification of the major rabbit and guinea pig semen coagulum proteins and description of the diversity of the REST gene locus in the mammalian clade Glires
Source: PLoS One. 2020 Oct 14;15(10):e0240607. doi: 10.1371/journal.pone.0240607 (PMC7556508; doi:10.1371/journal.pone.0240607)
Supplement: S13 Fig — The DNA sequences were aligned using the computer program Clustal Omega, which was followed by minor manual adjustments of the aligned sequences Translated nucleotides are highlighted in green and non-translated in grey. (DOCX) [file pone.0240607.s015.docx]

**5’ end of the MCE in Svs2**

| Human SEMG2 | TGGAGATAATGAAT-GCATACATTTCTATTATCAATTACCAGGTGGA---TCAAAAGGCCAATTGCCAAGCGGAT |
| --- | --- |
| Jerboa | TGCGGATCATGTAA-CCAAACATTCTTGTTGTGAATTACTAGGTGAA---GCAAAAGGCTTCATTTTAGATAGTG |
| UGMBMR | TACAAATAATGTAA--CAAAGTTTCTTATTATGCATTACCAGGTGGTGAGGCAAAAGGCCACTTTGCAAGTAGCT |
| Rat | TGCAGATCATGTAACTTAAACCTTCCTCCTCTCAATTACCAGGTGGG---ACAAAAGGTCACTTCCAGAGCAGCT |
| Mouse | TGCAGATTACGTAACTTAAACATTCTTATCATCAATTACCAGGGGCT---ACAAAAGGCCACTTCCAGAGCAGCT |
| Hamster^1^ | TGCAGATTATATAA--CAAACGTTCTT---ATGAATTACTAGGGGAA---ACAAAAGGCGGCTTCACGAGTAGTT |
| Vole^1^ | TGCAGATGATATAA--CAAACATTCTTATTATAAATTACCAGGTGGA---ACAAAAGGTGGCTTCACTAGTAGCT |
| Deer mouse^1^ | TGCAGATAATATAA--CAAACATTCTTACTATAAATTTCCAGGTGGA---ATAAAAGGTGGCTTCACGAGTAGCT |
|  | ** **** *** * ** ***** ****** ************** ********** ** * ** * * |

^1^ Tandem repeats located between the nucleotides given with red font have been omitted.

* Nucleotide in human *SEMG2* that is preserved in at least 4 out of the 7 myomorph species

**3’ end of the MCE in Svs2**

| Human SEMG2 | TAGCAACCACTTGAAAAGCTGGACCAATAGCAAGGTAAGTTTGCTTTTCTTACCAAATAGGAGAGGTGCCTGTCC |
| --- | --- |
| Jerboa | TTCTCACCAACTGGAGAACAAGATCGCTAATAAGGTATG------TTCCTTACCATATAGGGGAGATACCTATCC |
| UGMBMR | CACTGGCCATCTGAAGATCAAAGTCGAGATCAAGGTATG------GTTCT-ACCAAATAGGAGAGCTATCTTATT |
| Rat | CACTGAGCAACTGAAGACCAAGATCAATGTCAAGGTATG------TTTCC-ACCAAGTAGGAAAGATATTTATCC |
| Mouse | CACTGACCAACTAAAGACCAAGATCAATATCAAGGTATG------TTTCT-ACCAAGTAGGAAAGATATTTATCC |
| Hamster | CATCGACCAACTAAAGACCAAGATCGTTATCAAGGTATG------TTTCT-ACCACATAGGAGACATATTTATCC |
| Vole | CATTGACCAACTAAAGCGCAAGATCAATATCAAGGTATG------TTTCT-ACC-AATAGGAGCGATATTTATCC |
| Deer mouse^2^ | CATTAACCAACTAAAGACCAAGGTCAATATCAAGGTATG------TTTCT-ACCAAATAGGAGAGATATTTATCC |
|  | * **** * ** * * ** ***** ******* * ***** ************** * * *** |

^2^ Because of a premature stop codon, it is unclear whether the given sequence is the true 3’ end of exon 2 in the deer mouse.

* Nucleotide in human *SEMG2* that is preserved in at least 4 out of the 7 myomorph species
