# Supplementary material for: Identification of the major rabbit and guinea pig semen coagulum proteins and description of the diversity of the REST gene locus in the mammalian clade Glires
Source: PLoS One. 2020 Oct 14;15(10):e0240607. doi: 10.1371/journal.pone.0240607 (PMC7556508; doi:10.1371/journal.pone.0240607)
Supplement: S14 Fig — The DNA sequences were aligned using the computer program Clustal Omega, which was followed by minor manual adjustments of the aligned sequences Translated nucleotides are highlighted in green and non-translated in grey. (DOCX) [file pone.0240607.s016.docx]

**5’ end of MCE in Svs3**

| Human SEMG2 | GATAATG-AATGCATACATTTCTATTATCAATTACCAGGTGGATCAAAAGGCCAATTGCCAAGCGGATCTTCCCAATTTCCACATGGACAAAAGGGCCAGCACTATTTTGGACAAAAAGA |
| --- | --- |
| Jerboa Svs3b | CGATATGTAACTCAAACATGTTTGTGATTGGTTACCAGGTGAGGCAAAAGGCTACATTCCACAAGAAGCATCCCAGTTCTCAATTAAACATGCAGATTACTTCCACCATGGATACAAAGG |
| UGMBMR Svs3b | GATAATATGATTCAAACATTCTTGTTATCAATTACCAGGTGAAGCGAAGGGCCACTTTCCAGTTAAATCATCCCAATTTCTATTTGGTCAAAAAGGCAGTTTCCAGTATGGGAGGAAAGG |
| Rat Svs3a | GAAAACTTAACTCAG--ATTCTTGTTATCCATTACCAGGTGGGACAAAAGGACACTTTCTAGTAAAAACATCCCCACTCGTATTTATCGATAAAGGCCAGTTCCTCTATGGACACAGAGA |
| Rat Svs3b | GAAAACTTAACTCAG--ATTCTTGTTATCCATTACCAGGTGGGACAAAAGGACACTTTCTAGTAAAAACATCCCCACTCGTATTTATCGATAAAGGCCAGTTCCTCTATGGACACAGAGA |
| Mouse Svs3a | GATAATGTAACTCAG--ATTCTTGTCATCCATTAGCAGGTGGAACAAAAGGACACTTCCTAGTAAAAACCTCCCCACTCATGTTTATTGGGAAAAACCAGTTCCTCTATGGGCACAAAGA |
| Mouse Svs3 | GATAATGTAACTCAG--ATTCTTGTCATCCATTAGCAGGTGGAACAAAAGGACACTTCCTAGTAAAAACCTCACCACTCATGTTTATTGGGAAAAGCCAGTTCCTCTATGGGCACAAAGA |
| Hamster Svs3a | GAGAGTGTAACTCAAACATTT-TGTTATCAATTACCAGGTGGGCCAAAAGGACACTTTCTAGTAAGAACACCCCCAGTTGTATTTATCCAAAAAGGCCATTTCCACTATGGGCCCAGCGA |
| Ψ Hamster Svs3b | GAGAGTATAACTCAAACATTTT-GTTATCAATTACCAGGTGGGCCAAAAGGACACTTTCTAGTAAGAACACCCCCAGTTGTATTTATCCAAAAAGGCCATTTCCACTATGGGCCCAGCGA |
| Vole Svs3a | GACAGTGTAACTCAAACATTCTTTTTATC-ATTACCAGGTGAGACAAAAGGACACTTTCTAGTAAACACATCCCCAGTTGTATATTTCCAGAATGACCATTCCCAGTATGGGTCCAGAAG |
| Vole Svs3b | GACAGTGTAACTCAAACATTCTTTTTATC-ATTACCAGGTGAGACAAAAGGACACTTTCTAGTAAAAACATCCCCAGTTGTATATTTCCAGAAAGACCATCTCCAGTATGGGTCCAGAAG |
| Deer mouse Svs3a | GACAATGTAACTCAAACATTCTTGTTATCAATTACCAGGTGGGAGAAAAGGACACTTTCTAGTAAAATCGCCCCCAGTTGTATTTATCCAAAAAGGCCATTTCCACTACGGGCCCAGAGG |
| Deer mouse Svs3b | GACAATGTAACTCAAACATTCTTGTTATCAATTACCAGGTGGGAGAAAAGGACACTTTCTATTAAAATCGCCCCCAGTTGTATTTATCCAAAAAGGCCATTTCCACTACAGGCCCAGAGG |
|  | ** **** ** ** **** * ***** ************ ******* ** ** * * * * **** * ** * * ** ** ***** * * * *** * * ** |

* Nucleotide in human *SEMG2* that is preserved in at least 4 out of the 7 myomorph species

**3’ end of MCE in Svs3**

| Human SEMG2 | CACTTGAAAAGCT-GGACCAATAGCAAGGTAAGTTTGCTTTTCTTACCAAATAGGAGAGGTGCCTGTCCCAAAGT |
| --- | --- |
| Jerboa Svs3b | CCGGCTGAAGACCAGGGTCTATGTTAAGGTAAG------TCTCTTCAGCGACTGGGGAAATGCCTATCCCAGTGC |
| UGMBMR Svs3b | CCAATTAAGGACCAGGACACATTTTGAGGTAAG------TTACTTTACAAATAGGGGTGAGCTCTATCACAGTGT |
| Rat Svs3a | TCTCTCAAGAATTAAAACACGTGTAGAGGTAAG------TATCTTTGCAAACAGAAGAGCTGTCTACCTCAGTGT |
| Rat Svs3b | TCTCTCAAGAATTAAAACACGTGTAGAGGTAAG-------TACTTTGCAAACAGAAGAGCTGTCTACCTCAGTGT |
| Mouse Svs3a | CCTCTCAAGAATTAAGACACATTTAGAGGTAAG------TGTCTTAGCAAACGGGAGAGCTGTCTGCCCCAGTGT |
| Mouse Svs3 | CCTCTCAAGAATTAAGACACATTTAGAGGTAAG------TGTCTTAGCAAACGGGAGAGCTATCTGCCCCAGTAT |
| Hamster Svs3a | TCAGGCAAGGACCAGGACACATTTAGAGGTAAG------TGTCTTACCAAACAGGAGAGACATCTCCCCCAGTGT |
| Ψ Hamster Svs3b | TCAGGCAAGGACCAGGACACATTTAGAGGTAAG------TGTCTTACCAAACAGGAGAGACATCTCCCCCAGTGT |
| Vole Svs3a | ATCGGTCAAGGCCAGGACACATTTAGAGGTAAG------TGTCTCAGCAGGCGGGAGAGCTATCTA-CCCAGTGT |
| Voue Svs3b | ATCGGTCAAGGCCAGGACACATTTAGAGGTAAG------TGTCTCAGCAGGCGGGAGAGCTATCTA-CCCAGTGT |
| Deer mouse Svs3a | TCAGTCAAGGATCAGGACACATTTAGAGGTAAG------TGTCGTACCAAACAGAAGAGCTAACTACCCCTGTGC |
| Deer mouse Svs3b | TCAGTCAAGGATCAGGACACATTGAGAGGTAAG------TGTCTTACCAAACAGGAGAGCTAGCTACCCCTGTGC |
|  | * ** * **** * ******* * ***** **** ****** ** ** **** ** |

* Nucleotide in human *SEMG2* that is preserved in at least 4 out of the 7 myomorph species
