# Supplementary material for: Identification of the major rabbit and guinea pig semen coagulum proteins and description of the diversity of the REST gene locus in the mammalian clade Glires
Source: PLoS One. 2020 Oct 14;15(10):e0240607. doi: 10.1371/journal.pone.0240607 (PMC7556508; doi:10.1371/journal.pone.0240607)
Supplement: S15 Fig — The DNA sequences were aligned using the computer program Clustal Omega, which was followed by minor manual adjustments of the aligned sequences Translated nucleotides are highlighted in green and non-translated in grey. (DOCX) [file pone.0240607.s017.docx]

**5’ end of MCE in Svs4**

| Human SEMG2 | TTTCTATTATCAATTACCAGGTGGAT--CAAAAGGCCAATTGCCAAGCGGATCTTCCCAATTTCCACATGGACAAAAGGGCCAGCACTATTTTGGACAAA | 418 |
| --- | --- | --- |
| Jerboa | TTGTTATTGTTCATTATCAGGAGAG---CAAAAGGACAGTTGCTAAATAGACCATCCCTATTTCT<i>TAGTCAAAAG-------ACATTATAGACCACA | 885 |
| UGMBMR | TTCTTATTGTGGAGAATCAGAAGAGG--AGCAAGGGCATTTGCTGGGTAGATCGTTCCAATCTGTAACTAGACAAAAGGTCAACGACATTATAGAAAACA | 405 |
| Rat | TTCTTATTGTCCAGTATAAGCGGAGT--AGCAAGGACAGTCGCCGG--ACATCAAGCCTATTTCTGACTAGACAGAAGATCCAGGAGTTTCTAGACAGCC | 420 |
| Mouse | TTCTTATTGTTCATTATAAGGAGAGTGTATCAAGGGCAGTTTCCAAAC<i>TTAAGCCTATTTCTGACTAGACAAAAGATCAAGGAATTTCTAGACAACA | 837 |
| Hamster | TTCTTATTGTTAATTATTAGAGGAGG--AGCAAGGACAGTTGCCAAGTAGATC<i>CCTATTTCTAACTAGGCAAAAGACTAAGGAATTTCTAAACAACA | 611 |
| Deer mouse | TTCTTATTGTTGATTGTTAGGAAATG--AGCAAGGACAGTTGCCAAGTAGATGAAGTCTATTTCTAACTAGACAAAAGATCGAGGGCTTTCTAGACAACA | 418 |
|  | ** **** * **** ** ** **** ** ******* **** ** ***** * ******** * ** * * * * * * * |  |

| Human SEMG2 | AAGACCAACAACATACTAAATCCAAAGGCAGTTTTTCTA < 51 bp> GTCAGCAATATGATTTGAATGCCCTACATAAGGCGACAAAATCAAAACAAC | 559 |
| --- | --- | --- |
| Jerboa | AA-GCAGGCAACAAGCTGTATCCCAAGATAGGTTTTCAC < 76 bp> TAACGAATCCTATTTTTCTCTCAACAGACAAAGTCCGTCAAAAGTCTGAAG | 1050 |
| UGMBMR | GA-ACAAGCAGTAAGTTGCATCCAAAGACAGGTTATTAA < 76 bp> TAATGAAACATACTTTTCTCTCAACAGAAAAATTCCGACAATCTGAAGAAA | 570 |
| Rat | A----AAAAGGCAAATTGCACCACACGATAGGTTACTAA < 76 bp> GAACAGATCATGCTTTTCTCTCGTCAGAAAAATACTCACAGTCGGAAGAAG | 582 |
| Mouse | A----AAATGGCAAATTGCATCACAAGATAGGTTACTAA < 70 bp> GAGCAAAGCCTGTTTTTCTCTCATCAGAAAAATTCTTGCAGTCGGAAGAAA | 993 |
| Hamster | ----CAAAAGACAAGTGGTACCATAAGATAACTCACTAA < 74 bp> GAATAAAACATGTTTTTCTCTCATCAGAAAAAATATCACAAGAGTCTGAGG | 771 |
| Deer mouse | ----TAGAAGACAAGTTACATCACAAGATTGGGTGCTAA < 76 bp> GAATAAAACATGTTTTTCTCTCATCAGAAAAAATGTTTCAGTCTGAAGAGT | 580 |
|  | ** ** * *** *** ** ** * * *** *** * * * ** * ** ** ** |  |

<i> insertions encompassing 412 bp ERV class II repeat in the mouse, 305 bp SINE B4 repeat in Jerboa, and 87 bp SINE Alu/B1 repeat in Hamster

* Nucleotide in human *SEMG2* that is preserved in at least 4 out of 6 myomorph species

**3’ end of MCE in Svs4**

| Human SEMG2 | AACCACTTGAAAAGCTGGACCAATAGCAAGGTAAGTTTGCTTTTCTTACCAAATAGGAGAGGTGCCTGTCCCAAAGTTGGGGACTC-TCCAGGAACATGG |
| --- | --- |
| Jerboa | GACCAAAGGAAGTCCCGGACCAATATCAAGGTAAC------GCTTTGACCTAATAAGGGAGATGCCTACCCCAGGGCTGGGATGTGGTACCTAGGCCCGT |
| UGMBMR | AGCCAGCTGAAGACCCAGACCAATATTATGGTAAG-------GTTTTACCAAATAAGAGAGATAACTACCGCAGTGTTTGGAAGTTGTATGTGGGTACAC |
| Rat | GGGCAGCTGAACATCTGGACCAATATGCCGGTGAG------GATATGCCTGAGTGAGGCGGATGCCTACCC-AAGCTTTAGAAGTTGTGCATGGCAGAGG |
| Mouse | GACCAGCAGAAGACCTGGACCAACATGATGGTGAG------GATTTTCCCTAGTTAGGGAGATGCCTATCC-AGTGTTTAGAAGTTGTGCATGGGTACTC |
| Hamster | GACCAACAGAAGACCTGGACCAATATGATGGTGAG------AATTTTCCCTAGTAGGGGAGTTGTCTACCC-ACTGTTTAGAAGTTGTGCATGGGTACTC |
| Deer mouse | GACCAGCAGAAGACCTGGACCAACATGATGGTGAG------GATTTTCCCTAGTTAGGGAGATGCCTATCC-AGTGTTTAGAAGTTGTGCATGGGTACTC |
|  | **** *** * *********** * *** ** * ** ** * ** * *** ***** ** * *** * * * * ** * * |

* Nucleotide in human *SEMG2* that is preserved in at least 4 out of 6 myomorph species
