# Supplementary material for: Identification of the major rabbit and guinea pig semen coagulum proteins and description of the diversity of the REST gene locus in the mammalian clade Glires
Source: PLoS One. 2020 Oct 14;15(10):e0240607. doi: 10.1371/journal.pone.0240607 (PMC7556508; doi:10.1371/journal.pone.0240607)
Supplement: S16 Fig — The DNA sequences were aligned using the computer program Clustal Omega, which was followed by minor manual adjustments of the aligned sequences Translated nucleotides are highlighted in green and non-translated in grey. (DOCX) [file pone.0240607.s018.docx]

**5’ end of MCE in Svs5**

| Human SEMG2 | AATGCATACATTTCTATTATCAATTAC-CAGGTGGATCAAAAGGCCAATTGCCAAGCGGATCTTCCCAATTTCCACATGGACAAAAGGGCCAGCACTATT 409 |
| --- | --- |
| UGMBMR | CACTCAAATATCCTTATGATCAATTATGATGGCAGGATCAAGGACAGTTT-CCAAGTACATTATTCCTATTTGTAAATAGACAAAA-GGTCAGTGACATA 1673 |
| Brown rat | ATCGGCATCGGGTAACATCCTCGTTGT-CAGGTGTG---AAGGGCTGTTTGACAAGTAGATCACGGCAATTTCTAACCAGGCTAAAAAGTCAATTATTTT 393 |
| House mouse | ATCTGTATCAGGTAGCATCCTCATTGT-CAGGTGTG---AAGGGCTGTTTGACAAGTAGATCACTGCAATTTCTAACTAGGCTAAAAAGTCAATGGCTTT 384 |
| Hamster | AATACAAACATCCTCGTTGTCAATTGTGAGGGGAGTTGAGAGGGCCATTTGACAAGTAGACCATTCCAGTTTCTAACTGGACTAAAAGCTCAATGGCTTT 414 |
| Deer mouse^1^ | ---------------------------------------------------------------------------------------------------- |
|  | * ** * *** ** ** *** *** **** **** ******* * * * * *** * ** ** |

| Human SEMG2 | TTGG---ACAAAAAGACCAACAACATACTA<82 bp>TGATTTGAATGCCCTACATAAGGCGACAAAATCAAAACAACACCTAGGTGGAAGTCAACAACT 581 |
| --- | --- |
| UGMBMR | ATAGACAACATAACAGACAACAAGTTACAT<74 bp>AGAGACAGCTAATGAGGCATAG-TTTTTCTCTTAACAGGAAAATTCCAGAAATCCTTTGAAGA 1839 |
| Brown rat | ATAAGCAACAAAACAT-CAGCACGGTATCT<69 bp>TGGATATAGAGAATGAGACATG--TTTTCTCTCAACAGAAAAATTCTCACAGTCGGCTGAAGA 552 |
| House mouse | ATAAACAACAAAACAG-CAACAGGGTACAT<85 bp>TGGACATAGAGAATGAGACACG-CTTTTCTCTCAACAGAAAGGTTCTCTCAGTCGGCGGAAGA 560 |
| Hamster | GTAGGCAGCAAAACAG-CAACAAGGTACAT<84 bp>TGGGCAGAGAGAATTAGGCGTG-TTTTTCTCTTCACAGAAAGATTTTCACAAGACACATCTAG 589 |
| Deer mouse | ------------------------------<48 bp>TGAACATAGAGAATCAGGCATG-CTTTTCTCTCCACAGAAAAATTCTCACAAGCGTCTGACGA 392 |
|  | * ****** ***** *** *** * * * * **** * ** * * ** |

^1^ Nucleotide sequences homologous with MCE in *SEMG2* is deleted in the first intron of deer mouse *Svs5*.

* Nucleotide in human *SEMG2* that is preserved in at least 3 out of 5 myomorph species

**3’ end of MCE in Svs5**

| Human SEMG2 | CAACCACTTGAAAAGCTGGACCAATAGCAAGGTAAGTTTGCTTTTCTTACCAAATAGGAGAGGTGCCTGTCCCAAAG-TTGGGGACTCTCCAGGAACATG |
| --- | --- |
| UGMBMR | GGATCGGCTGAAGACATGGACCGAAATTATGGTAAG------GGTCTTACCAAATAAGGGAGATGTCTACCCCAATGTTTAGAAGTTGTGAATA--TGTA |
| Brown rat | CGGCCAACTGAAGACTTGAGCCAATAGGCAGGTAAG-----TGTTATCACCAGGTGAGGGC-----------------TTACAAGCTACTCGTGCCTAAT |
| House mouse | TGGCCATCTGAAGACTAGAGCCAGTATGCAGGTAAG------TGGATCACGGGGTGAGGGG-----------------TTAGAAGCTACACATACCTATT |
| Hamster | TGGCCAACTGAAGACCTGGGCCAATATGAAGGTAAG------AATATCGCCAGGTGAGGGAGATGCCTACCTCAGGGTTTTGAAGCTATGCATGGCTATT |
| Voule | TGGCCAGCTGAAGATCTGGACCAATATAAAGGTAAG------GATATTACCAGGTACCCGAGATGCCCGCCTAAGCATTTCAAAGTTATGCATGGCTATT |
| Deer mouse | TGGC-AGCTGAAGACCTGAACCAATATGAAGGTAAG------AATATTATCAGGTGAGGGGGAGGCCTGCCTCAGGGTTTCCAAGTTATGCATGGCTATT |
|  | *** **** * ** ****** ******** * ****** * * * * * ** * * ** * * ** * ** |

* Nucleotide in human *SEMG2* that is preserved in at least 4 out of 6 myomorph species
