# Supplementary material for: Identification of the major rabbit and guinea pig semen coagulum proteins and description of the diversity of the REST gene locus in the mammalian clade Glires
Source: PLoS One. 2020 Oct 14;15(10):e0240607. doi: 10.1371/journal.pone.0240607 (PMC7556508; doi:10.1371/journal.pone.0240607)
Supplement: S17 Fig — The DNA sequences were aligned using the computer program Clustal Omega, which was followed by minor manual adjustments of the aligned sequences Translated nucleotides are highlighted in green and non-translated in grey. (DOCX) [file pone.0240607.s019.docx]

**5’ end of MCE in Svs6**

| Human SEMG2 | CATTTCTATTATC--AATTACCAGGTGGA--------TCAAAAGGCCAATTGCCAAGCGGATCTTCCCAATTTCCACATGGACAAAAGGGCCAGCACTAT 408 |
| --- | --- |
| UGMBMR | AAACTCAAATATTCTTATTGTGGAGAATCAGGAGAGGAGCAAGGGC-ATTTGCTGTGTAGATCGTTCCAATTTGTAACTAGACAAAAG-GTCAACGACAT 400 |
| Brown rat | ATTGGCTAACATCCTCATTGTTAGGTGTG-----------AAGGGCTGTTTGACAAGTAGATCACAGCAATTTCTAACTAGGCTAAAAAGTCAATGGTTT 393 |
| House mouse | CAGG--TAGCATCCTCATTGTCAGGTGTG-----------AAGGGCTGTTTGACAAGTAGATCACTGCAATTTCTAACTAGGCTAAAAAGTCAATGGCTT 407 |
| Hamster | AATA-CAAACATCCTCGTTGTCAATTGTGAGGGGAGTTGAGAGGGCCATTTGACAAGTAGATCATTCCAATTTCTAACTGGACTGAAAGCTCAATGGCTT 413 |
| Voule | GGTAACAAACACCCTCATTGTCAGCTGTG-----------AGGGG--ATTTGACAAGTAGATCATTCCAACTTCTAACTAGACTAAAAGGTTAATGACTT 399 |
| Deer mouse^1^ | ---------------------------------------------------------------------------------------------------- |
|  | * * *** *** * ** ** *** *** **** **** ******** * * * * *** * ** * |

| Human SEMG2 | TTTGGACAAAAAGACCAACAACATACTAAATC<67 bp>GTCAGCAATATGATTTGAATGCCCTACATAAGGCGACAAAATCAAAACAACACCTAGGTGG 569 |
| --- | --- |
| UGMBMR | TATAGAAAACAGAACAAGCAGTAAGTTGCATC<79 bp>AGAGATAGCTAATGAAGCATACTTTTCTCTCAACAGAAAAATTCCAACAGTCATCTGAAGA 573 |
| Brown rat | TATAAGCAACAAAAC-ATCAGCACAGTACCTC<68 bp>GGATATAGAGAATGAGACATG-TTTTCTCTCAACAGAAAAATTCTCACAGTCGGCTGAAGA 553 |
| House mouse | TATAAACAACAAAAC-AGCAACAGGGTACATC<84 bp>GGACATAGAGAATGAGACACGCTTTTCTCTCAACAGAAAGGTTCTCGCAAGCAATTGAAGA 584 |
| Hamster | TGTAGGCAGCAAAAC-AGCAACAAGGTACATC<83 bp>GGGCAGAGAGAATTAGGCGTGTTTTTCTCTTCACAGAAAGATTTTCACAAACATCTGAAGA 589 |
| Voule | CATAGACAACAAAAC-AGCAACAAGGTAGATC<80 bp>GGACATAGGGAGTGAGACAGTCTTTTCTCTCCACAGAAAAGTTCTCACAATCATCTGAAGA 572 |
| Deer mouse | --------------------------------<50 bp>GAACATAGAGAATCAGGCATGCTTTTCTCTCCACAGAAAAATTCTCACAAGCGTCCGACGA 392 |
|  | * * *** ** ** * ** ** ** *** * * **** * * * ***** **** * * |

^1^ Nucleotide sequence homologous with MCE in *SEMG2* is deleted in the first intron of deer mouse *Svs6*.

* Nucleotide in human *SEMG2* that is preserved in at least 4 out of 6 myomorph species

**3’ end of MCE in Svs6**

| Human SEMG2 | ACCACTTGAAAAGCTGGACCAATAGCAAGGTAAGTTTGCTTTTCTTACCAAATAGGAGAGGTGCCTGTCCC---AAAGTTGGGGACTCT--CCAGGAACA |
| --- | --- |
| UGMBMR | GCCAGCTGAAGACCCAGACCAATATTATGGTAGG--------TTTTACCAGATAAGAGAGATAACTACCAC--AGTGTTTGGAAGTTGTACGTGAGTACA |
| Brown rat | AGGAACTGAAGACCTGGACAGAAATAATGGTAAG------GATCTTACCAGGCCTGGGGGATG-CTAACCC--AGTGTTAG-AAGTTGTGGGTGGGTACT |
| House mouse | AGGAACTGAAAACCTGGACAGAAATGATGGTAAG------GATCTTAGCAGGTATGGGGGAGGTCAACCCC--AGTGTTAG-AAGTTGTGGGTGGGTACT |
| Hamster | ACCAGCGGAAGACCTGGACCAATATGAAGGTAAG------GTTATTGCTAGGTGAGGGAGACACC-----------TTTCT-CAGCCATGCATGAGTACT |
| Voule | ACCAGCTGAAGATCCGGATCAATACAAAGGTAAG------GATATTGCTAGGGGAGGGAGACACACTCACCCCAGAGTTTAGAAGTTGTGCATAAGTACT |
| Deer mouse | ACCAGCTGAAGGCCTGCATCAATATGAAGGTAAA------GATGGTGCTAGGTGAGGGAGACCCCTACCCC--AGGGTTTAGAAGTGGTGTGTGAGGACT |
|  | **** **** * *********** ******** * ** * * * * *** * *** ** * * * ** |

* Nucleotide in human *SEMG*2 that is preserved in at least 4 out of 6 myomorph species
