# Supplementary material for: Identification of the major rabbit and guinea pig semen coagulum proteins and description of the diversity of the REST gene locus in the mammalian clade Glires
Source: PLoS One. 2020 Oct 14;15(10):e0240607. doi: 10.1371/journal.pone.0240607 (PMC7556508; doi:10.1371/journal.pone.0240607)
Supplement: S19 Fig — The amino terminal tandem repeats and the extended central repeat region were removed from the sequences, which were then aligned with Clustal Omega, followed by some minor manual adjustment. The upper part shows the aligned sequences with amino-terminal tandem repeats, highlighted in green or blue, reinserted. The underlined sequences are encoded by SPCE (exon1) and the conserved Cys preceding the central repeat regions are highlighted in grey. A frame shifted sequence, leading to premature stop, in the deer mouse is written with red font. The lower part displays the central repeat regions grouped according to suborder. The sequences were manually aligned in order to demonstrate conserved sequences both within and at the termini of the repeats. Some of the longer, perfectly conserved repeats are underlined. The hydrophobic AA, surrounded by Gln and Lys at the center of the poorly defined tandem repeats are highlighted in red and similar repeats with a different central residue are highlighted in purple. Aromatic residues are highlighted in yellow. (DOCX) [file pone.0240607.s021.docx]

Jerboa MMPTIFLTLSLLFTVQTHAAQLGFHG-EA-----------------------------------------KGFILDSAAQ--VTKQ--------------NSLLNHGQKGMQELITKESI

UGMBMR MKSTVFI-LSLILFLEKQVAGIGFYGGEA-----------------------------------------KGHFASSSSK-FLFGQKGGLSYGNKGGSEGKGGLSYGNQGGSEGAAEERM

Rat MKSSIF-ILSLFLLLERQAAVVGQYGGT------------------------------------------KGHFQSSSS-GFMLGQ--------------KGHLNFGLKGGSEEAAEESI

Mouse MKSSVF-VLSLLLILERQSAVVGQYGAT------------------------------------------KGHFQSSSSEGFMLGQ--------------KGRLSFGIKGGSDEAAEESL

Hamster MKSPVF-ILSLLLILERQASVVGFYGETKGGSFTKGGSFTKGGSFTKGGSFTSGGSFTKGGSFTKGGSFTKGGFTSSSSE-FM-----------------KGHVNYGLKGASEDAAEESV

Vole MKSSVF-ILSLLLILERQDAVVGQYGGTKGG-FTKGG-FTKGG-FTKGG-------------FTKGG-FTKGGFTSSSS-GSM-----------------KGHVSYGLKGGSDDVAEESV

Deer mouse MKSLVF-ILSLLLILERQAAVVGQYGGIKGG-FTKGG-FTKGG-FTKGG-------FTKGG-FTKGG-FTKGGFTSSSS-GFM-----------------KGHVSYGLKGGSEDAAQESA

*** ** ** * ***** **** ** ** * **** ** ** * ***** *****

Jerboa FRQAKGQVQDHGGDI---KEMHVSQIHRGVKDR-PCHVEQITKRKSQD----------- <---- 0 AA -----> ----------------------ISQKTKQKGFAAVKTQTQ

UGMBMR FVQTKHRVSDQDGDM---KQTRISQTLTGRKAATRCSNEQIARRKFQDSQMKSH----- <---- 45 AA -----> ----------------------FTQQSKQKGFAMDEELSG

Rat FMQSQHQMFGQDGGDM--AQTSVSQEHTGVKGAAICRKGQVSQLKSQESQIKSFRQVKS <---- 172 AA -----> FSQVKSQSAQLKSFGQQKSLKGFSQQTQQKGFAMDEDLSQ

Mouse FMQSQRRVYGQGGGDM--TQTRVSQEHTSVKGAALCRNGQVSQLKSQESQIKSYGQVKS <---- 133 AA -----> FSQVKSQSSQLKSYGQQKSLKGFSQQTQHKGFAMDEGMSQ

Hamster FLQTKHQAYDQGNGM---THTRLSQEHTGVKGASLCRIGQMSQLKSQESQVKSFGQVKS <---- 134 AA -----> FGQLKSQDAQLKSFGQQKSLKGFSQQTQHKGFAMNEELSQ

Vole FMQTKHQVYGQDGEM---SQSHLSQEHTGVKGAALCRKGQVSQLKSQESQVKSYGQVNS <---- ND -----> YGQAKSQDTQLKSFGQQKSLKGFSQQTKHKGFAMNEELSQ

Deer mouse FLQTKQQAYSKGGSMQQVQQARLSQEHTGVKGAALCRKGQTSQIKSQESQIKSFGQAKS <---- 111 AA -----> FGQLKSQSGQLKSFGQTKSLKGFSQQTQQKGFAMGGGLNS

* * * * * ********** * *** ** ****** ** * * * *** **** ** ********** ***** * ***

Jerboa TVHFHNHQGEEQGKLKNKFHQIIQSKEV----FPHYGQQRSHSYEDYLMRYKQQGHNLDQQQRQSTQDGVHHTYAKERLVLYQSQSKI

UGMBMR VH----TKSEEDVQQVQEKSGQYSKTGS----SAQFGQQRSQSYEGYLEQYKQKLQDHYQQRKNFNQDKMQQYFSKGGSEMYQRQYKE

Rat VRKQFDDD-DLSVQQKSTQQMKTEEDLSQFGQQRQFGQERSQSYKGYLAQYRKKLQEQ-QQQKNFNQD---NFFTKGGAGLYQAQLKG

Mouse VRKQFSDD-DLSVQQKSTQQMKTEEDLSQFGQQRQYGQERSQSYKGYLEQYRKKVQE--QQRKNFNPG---NYFTKGGADLYQAQLKG

Hamster VRKQYDEDDDQSVQQKSSQQVKTGEDL-QFGQQRQFGQEHSQSYKGYLEQYKKKSQDHYQQRKDFNQD---GYFTKGGADLYQVQLKG

Vole VRKQYNDD-ESSVQQKSS-QVKTEEDLAQFGQQRQFGQERSQSYKGYLEQYKKKSQDHYQQRRNFNQD---IYFTKGGADLYQTQLKG

Deer mouse AERICYGWRAITSA*

* **** * ** ***** *** ** * * ** **** * *** *** * *

Central repeat regions

Rat SGQLKSGGSQLKSFGQVKSSESQLKSFGQVKASGSQLKSFGQVKASGSQLKSYGQMKSSGSQVKSFGQMKSSGSQVKSFGQMKASESQIK

Mouse SGQLKSGGSAFGQVKSSVSQIKSYGQLKSGGQLKSGGPAFGQVKSQESQIKSYGQLKSSGQLKSGGSAFGQVKSSVSQIKSYGQLKSGGS

*******

Rat SFGQRKSQGGQLQSYGQMKSYGQTKSLESQAKSFGQVKSQSGQMKSSYGQRKSYGEETQLKSF----DQDAQLKSYGQQKSQKQSS

Mouse QVK-------------------------------------------SYGQTKSYGEEGQLNSFSQLKSQGAQLKSYGQQKSQQQSS

**** ****** ** ** * ************ ***

Deer mouse --------------LGSQVKSYGQMKSYGQVKSQESQVKSYGQMKSYGQLKSQNAQLKSHGQLKSQDAQLKSYGQLKSFGEDAQLKSYAQQKSQVAQQ--------------SFGQLKSQSGQLKSFGQLKSQSGQLKS

Vole HESQLKSSYGRLKSQNSQVKSYGXXXXXXXXXX <--------------- Sequence of undetermined size is missing in database --------------> XXXXXXXXXXKSQDAQLKS

Hamster HESQLKSSYGQLKSENAQVKSFGQVKSYGQVKSHESQLKS-----SYGQLKSQSAQLKSYGQLKSQDAQLKSFGQQKSYGEEAQLKSYGQLKFQDAQLKSYGQLKSQDAQLKSYGQLKSQEAQLKSYGQLKSQDAQLKS

**** ** ******** *** ** ******** ***** ************ ** ** ** ****** * * ** * ****** **** ****** ****

UGMBMR NSQLRSQEGQLNSHIQIKSQDTQLRSQESRAKSNVQRKSQGSQDF
