# Supplementary material for: Identification of the major rabbit and guinea pig semen coagulum proteins and description of the diversity of the REST gene locus in the mammalian clade Glires
Source: PLoS One. 2020 Oct 14;15(10):e0240607. doi: 10.1371/journal.pone.0240607 (PMC7556508; doi:10.1371/journal.pone.0240607)
Supplement: S20 Fig — Sequences were aligned with Clustal Omega. The sequences encoded by SPCE are underlined and conserved Cys are highlighted in grey. Tripeptides sequences of Gln and Lys surrounding a hydrophobic or a non-hydrophobic residue are highlighted in red and purple respectively. (DOCX) [file pone.0240607.s022.docx]

Rat Svs3a MKSIFFSLSLLLLLEKQAAGIGIYGGTKGHFLVKTSPLVFIDKGQFLYGHREEQEEAPEESILVQTKHHVYSQDA-DADTAEAHGSQKQT

Rat Svs3b MKSIFFSLSLLLLLEKQAAGIGIYGGTKGHFLVKTSPLVFIDKGQFLYGHREEQEEAPEESILVQTKHHVYSQDA-DADTAEAHGSQKQT

Mouse Svs3a MKSIFFSLSLLLLLEKKAAGIELYGGTKGHFLVKTSPLMFIGKNQFLYGHKEEQEEAPEESIFVQTKHHEYGQDA-DADMGGALSSQELT

Mouse Svs3b MKSIFFSLSLLLLLEKKAAGIELYGGTKGHFLVKTSPLMFIGKSQFLYGHKEEQEEAPEESIFVQTKHHAYGQDA-DADMGEALSSQELT

Voule Svs3a MKSIFFSLSLLLLLEKQAVGTGIYGETKGHFLVNTSPVVYFQNDHSQYGSRRAQEDGAEESSFEQTKHRVYGQDA-DVDLGETQSSQQQT

Voule Svs3b MKSIFFSLSLLLLLEKQAAGIGIYGETKGHFLVKTSPVVYFQKDHLQYGSRRAQEDGAEESSFEQTKHRVYGQDA-DADLGETQSSQQQT

Hamster Svs3a MKSIFFSLSLLLLLGKQAAGIGTYGGPKGHFLVRTPPVVFIQKGHFHYGPSEEREAASEESVFTQTKQHAYSQDA-DAEVRETQSSQEQT

Deer mouse Svs3a MKSIFFSLSLLLLLEKEAAGIEIYGGRKGHFLVKSPPVVFIQKGHFHYGPRGAQEDEPEGSVVVQTKHHVYRQDAAEADLGETQSSQQQT

Deer mouse Svs3b MKSIFFSLSLLLLLEKEAAGIEIYGGRKGHFLLKSPPVVFIQKGHFHYRPRGAQEDEPEGSVVVQTKHHVYRQDAAEADLGETQSSQEQT

************** *:*.* ** *****:.: *:::: :.: * :* * * . ***:: * *** :.: : .**: *

Rat Svs3a GLKEDIVCDEEDELAQQKSQLKSQSQIKSQTQVKSHEAQVKSQTGQLKTAGQVKSQTKLKSHGASLKFYKAPLHLQKDVSQQQIKGKGYD

Rat Svs3b GLKEDIVCDEEDELAQQKSQLKSQSQIKSQTQVKSHEAQVKSQTGQLKTAGQVKSQTKLKSHGASLKFYKAPLHLQKDVSQQQIKGRGYD

Mouse Svs3a SLKEDIVCEEEDELAQQKSQLPSQSQIKSQTQVKSYAAQLKSQPGQLKTIGQVKSQTMLKSHGAPLKSFKARLNLREDIP-QQVKGRGYG

Mouse Svs3b SLKEDIVCEEEDELAQQKSQLPSQSQIKSQTQVKSYAAQLKSQPGQLKTIGQMKSQTMLKSHGAPLKSYKARLNLREDIP-QQVKGRGYG

Voule Svs3a GVSEDIVCNEEDEISQQKSRLQSHSQIKSQTQVKSHAAQVKSQTGQLKTLGQVKSQIKLKSHRAPLKSSRASQTLQEAFP-QQIKGKAHA

Voule Svs3b SVSEDIVCDKEDKISQQKPQLQSHSQIKSQTQVKSHAAQVKSQTGQLKTLGQVKSQIKLTSHRAPLKSSRASQTLQEAFP-QQIKGKAHA

Hamster Svs3a DLNEDIDCDEEDEISQQKSQLQSQSQIKSQAQLRSQEAQLKSQTGQRKTLGQVIAQVKLKSHSAPLKPYQAPLTLQKVSA-QQIKGKEYA

Deer mouse Svs3a GLNEDLVCDEEDEISQQKSQLKSQSQIKSQAQLKTGGVQVKSQTGQLKTLGQVKSQIKLKSYRAPLKSYQAAITLQEGLP-QQIKGKDYA

Deer mouse Svs3b GLNEDLVCDEEDEISQQKSQLKSQSQIKSQAQLKTSGAQLKSQTGQLKTLGQVKSQIKLKSYRAPLKSYQAAITLQKGLP-QQIKGKDYA

.:.**: *::**:::*** :* *:******:*::: .*:*** ** ** **: :* *.*: * ** :* *:: **:**: :

Rat Svs3a LHQDLPQVRQQHANVHRLKRKLGQSSKTVAFLP-IRHRFQPYH-GYFTQFQEHLHGSVHHTKSFHHGPGMCYCPNGGLMLYQGIFTE

Rat Svs3b LHQDLPQVRQQHANVHRLKRKLGQSSKTVAFLP-IRHRFQPYH-GYFMQFQEHLHGSVHHTKSFHHGPGMCYCPNGGLMLYQGIFTE

Mouse Svs3a LAEDLAQVRQQPAKVHRLKGKHRQSRKTAAFYPQFRRRSRPYP-RYFVQFQEQLQGSVHHTKSFYPGPGMCYCPRGGVILYQDAFTD

Mouse Svs3b LAEDLAQVRQQPAKVHRLKGKHRQSRKTAAFYPQFRRRSRPYP-RYFVQFQEQLQGSVHHTKSFYPGPGMCYCPRGGVILYQDAFTD

Voule Svs3a LDEDQAQVRQQHKMVHRLKSKLAWARRTAEFLPYFRRHFQGYD-GYFVQFQGQLQGGIHHTKPFHQAQQTCYCPNGELILYQDAFTE

Voule Svs3b LDEDQAQVCQQHKMVHRLKSKLAWARRTAEFLPYFRRHFQGYD-GYFVQFQGQLQGGIHHTKPFHQAQQTCYCPNGELILYQDAFTE

Hamster Svs3a LDEDLARVHQQDKKVHKLKRNLGWARKTAENLPHLRQHSQGYD-GYVMQFQEQLQGGIHHTKSLHQAQGMCYCPKGGLFLHQEVFAE

Deer mouse Svs3a LKEDLAQVRQQHKKVHSLQRKLGQARKTAAFFPYFRHHSQDYDDGYFVQFQEQLQGGIRHIKSFHRGHGACYCPKGGLTLYQDAFTE

Deer mouse Svs3b LKEDLAQVRQQHKKVHSLQRKLGQARKTAAFFPYFRHHSQDYDDGYFVQFQEQLQGGIRHIKSFHQGHGACYCPKGGLTLYQDAFTE

* :* :* ** ** *: : : :*. * :*:: : * *. *** :*:*.::* * :: . ****.* : *:* *::
