# Supplementary material for: Identification of the major rabbit and guinea pig semen coagulum proteins and description of the diversity of the REST gene locus in the mammalian clade Glires
Source: PLoS One. 2020 Oct 14;15(10):e0240607. doi: 10.1371/journal.pone.0240607 (PMC7556508; doi:10.1371/journal.pone.0240607)
Supplement: S21 Fig — The primary structures were manually aligned after removal of the central repeat regions. Stars (*) and colon (:) indicates fully and 3 out of 4 conserved residues respectively. Gln and Lys surrounding a hydrophobic or a non-hydrophobic residue are highlighted in red and purple respectively. (DOCX) [file pone.0240607.s023.docx]

Jerboa Svs3b MKSTIFFILPLIFIPYTQVAASGFVGEAKGYIPQEASQFSIKHADYFHHGYKGGLE-GPETGIVSATKRIHYDAA---DN 77

UGMBMR Svs3a MKSAVFFILPLLLILEKQASGIGIYGEAKGHFPVKSSQFLFGQKGSFQYGRKGARDDEPEENIFMQTKHHGHGQD---AD 77

Rat Svs3b MKS-IFFSLSLLLLLEKQAAGIGIYGGTKGHFLVKTSPLVFIDKGQFLYGHREEQEEAPEESILVQTKHHVYSQDA-DAD 78

Deer mouse Svs3b MKS-IFFSLSLLLLLEKEAAGIEIYGGRKGHFLLKSPPVVFIQKGHFHYRPRGAQEDEPEGSVVVQTKHHVYRQDAAEAD 79

*** ** * *:: ::::::::::* **:: : : :: :: * :: : : ** : :**:: : :: ::

Jerboa Svs3b SQPTHISQDNTAKKGGTRC-------<------ 0 AA ------>----LSQQTFQEVISEQNKQGMHTVKGEQIQK 123

UGMBMR Svs3a MEQTQISREHMGLKEDALCDE-TEMA<------ 211 AA ------>LTELKSQQAFS-QQTKHKGYAMNEHLSQLRHQ 344

Rat Svs3b TAEAHGSQKQTGLKEDIVCDEEDELA<------ 54 AA ------>KAPLHLQKDVSQQQIKGRGYDLHQDLPQVRQQ 190

Deer mouse Svs3b LGETQSSQEQTGLNEDLVCDEEDEIS<------ 54 AA ------>QAAITLQKGLP-QQIKGKDYALKEDLAQVRQQ 190

: *: :::::: *:: : * :: : : : : ::

Jerboa Svs3b HEEVPHVHG-FTQYTETKEILPQTRQERVYSV-EMV----------GIPKGSHIHQEQRHSHCAKGGLELFQS--KV 186

UGMBMR Svs3a HEDAQQIKRSLGQSTETAEFLPPFGHQHSQSY-EGYLVQYQQQLRNHAHQTKSLNQDQGECHCSRGGAIVYQQRFKV 420

Rat Svs3b HANVHRLKRKLGQSSKTVAFLPI-RHR-FQPY-HGYFMQFQEHLHGSVHHTKSFHHGPGMCYCPNGGLMLYQGIFTE 264

Deer mouse Svs3b HKKVHSLQRKLGQARKTAAFFPYFRHH-SQDYDDGYFVQFQEQLQGGIRHIKSFHQGHGACYCPKGGLTLYQDAFTE 266

* : : ::* * ::* : : : :: : : : :: :: : : * **: ::* :

Central repeat region

UGMBMR Svs3a QVKSYIQLNSQESQLKSQGGQRESQVQLKSPLAQVKSQGQPKSQFQVKSRFAQIKSQGQLKSQGQLKSPFTQVKSQGGQL 182

KSQFQEDSQLAQIKSQQSQLKSVDGQLKSQFQVKSQFAQIKSQGQLKKSPFTQLKSQQGHLKSHFSQIKSQGGQLTSPFA 262

QIKSQRGQLKSQQGQLKSQGQLKSQFQLNSQLAQIKSQQGQLKSQVQLKSP 313

Rat Svs3b QQKSQLKSQSQIKSQTQVKSHEAQVKSQTGQLKTAGQVKSQTKLKSHGASLKFY 158

Deer mouse Svs3b QQKSQLKSQSQIKSQAQLKTSGAQLKSQTGQLKTLGQVKSQIKLKSYRAPLKSY 159
