# Supplementary material for: Identification of the major rabbit and guinea pig semen coagulum proteins and description of the diversity of the REST gene locus in the mammalian clade Glires
Source: PLoS One. 2020 Oct 14;15(10):e0240607. doi: 10.1371/journal.pone.0240607 (PMC7556508; doi:10.1371/journal.pone.0240607)
Supplement: S22 Fig — Aligned sequences are shown with exon 2 and the beginning of exon 3 in rat and mouse highlighted in green. Positions with homology to the murine splice donor or acceptor site, or both, are mutated in non-murine genes. Potentially alternative splice donor and acceptor sites are highlighted in grey and purple respectively. The underlined sequence is the last of 6 tandem repeats in vole Svs6; for clarity were the first 5 omitted. Identical nucleotide in 5 or 6 species is indicated with a star (*). (DOCX) [file pone.0240607.s024.docx]

| Mouse | TTTTCTCTCAACAGAAAGGTTCTCGCAAGCAATTGAAGAG---TTTTCTAGTGAAAGTTCTGAAGTAAGTATAGTAGGGGGTGGGCAGTAGGGGGATCAA 642 |
| --- | --- |
| Rat | TTTTCTCTCAACAGAAAAATTCTCACAGTCGGCTGAAGAC---TTTTCTAGTGAAAGTTCTGAAGTAAGTATAATGGGGGGCGGGGGTGTATGTGATCAA 611 |
| Hamster | TTTTCTCTTCACAGAAAGATTTTCACAAACATCTGAAGACCAATCTTCTAGTGAAACCTCTGAAATAACTGCAACA-----GGGGGTAGCGGGGGATCAA 645 |
| Vole | TTTTCTCTCCACAGAAAAGTTCTCACAATCATCTGAAGAA---CATTCTAGTGAAATCTCTGAAGTCGGC-----------GGGAGCAGTGGTGGATCAA 619 |
| Deer mouse | TTTTCTCTCCACAGAAAAATTCTCACAAGCGTCTGACGAC-AACATTGATCTAAAGGCACTG-------------------GGGGGCAGCGGAGGATCCA 433 |
| UGMBMR | TTTTCTCTCAACAGAAAAATTCCAACAGTCATCTGAAGAT---GTCTCCAGTGAAAGCTTTGAGGTACATATGTTG-----GACAAAGGCCCGGGACTAA 626 |
|  | ********* ******* ******** * ******* *** ******* ***** * ** * ******* |

| Mouse | GTTCTACCCATGACAAACACG---GCCAAAGTGAAAACTCATGGTGTTCCTTTAAGGCAAATAGTCCAAAAAGCATTGTCCATGAGGAGGTTTATGAGGA 739 |
| --- | --- |
| Rat | GTTCTACCCGTGACAAACATGACGACAAAAGTGAAAACTCATGGTGTTCCTTTAAGGCAAAAATCCCCAAAAGCGTCATCCATGAGGATGTCTATGAGGA 711 |
| Hamster | CCACTACCCACCATG---AATACAGCCGGAGCGAGAGCTCATGGAGTTCCTTTAAACCAAAAAGTCCCCAAGGCAGCCTCACCGAGGAAGTCTATGAGGA 742 |
| Vole | GTTCTGCCAATGAGG---AATACAGCCGGAGTGAGAGCTCATGGAGCAGCTTTAAGTCAAAAAGTGCAAAGAGCAGTGTCGGCGAGGAAGGCTCTGATGG 986 |
| Deer mouse | GTTCTTCCCATGAAG---AATACAGCAGCAGTGAGGGCTCATGGAGCTCCTTTAAGTCAAAAAAACCGAAAAGCATCGTCACCGAGGAAGTCTATGAGGA 530 |
| UGMBMR | ACTCTGCTGAAGAAG---AATACAGCATAACTGAGAAAACACGGACTGGTTATCGTTCAGAGAAGCCCAAAAGCATGTACAGCGAGGAAATCTATGAGGA 723 |
|  | *** ** * ** * ** ** ***** * ******* * ****** **** * * *** *** ** ***** *********** |
