# Supplementary material for: Identification of the major rabbit and guinea pig semen coagulum proteins and description of the diversity of the REST gene locus in the mammalian clade Glires
Source: PLoS One. 2020 Oct 14;15(10):e0240607. doi: 10.1371/journal.pone.0240607 (PMC7556508; doi:10.1371/journal.pone.0240607)
Supplement: S23 Fig — The aligned products of functional genes are shown. Residues are highlighted in green if they are present in >50% of the sequences, i.e. 9 or more. The sequences highlighted in grey, in deer mouse Svs5 and Svs6, are in a different reading frame, due to conserved mutations. Sequences encoded by SPCE are depicted by a thick underlining. The sequence with the double underlining is the first copy of 6 tandem repeats present in vole Svs6. The remaining repeats were omitted prior to alignment. The complete tandem repeat region of vole Svs6 is shown below the aligned sequences. (DOCX) [file pone.0240607.s025.docx]

Svs4_Jerboa MKSAWFFLLSLLLLDATPPAEPKQHYKVRQKSEDVSSTELRLM-RSDKDLSHSSIRNEYRSDASSLFDMPSKRY--STLSEE---------DQRLSG--GKLLKKNLGSEEKFSHFSSRKKT-QYHQSSD- 115

Svs4_Deer_mouse MKSTSVFVLTLLLFLVTEVTGKK-KEKMFQSEE--SSSETFVSSQGDKRSSSGSSSEAYSLEKSS--SSRSKEPR-SSFSEESYEES----SHRS-HKKLGSGDTGGSNMESESSFSKKKKN-KFSQDQTE 119

Svs4_Rat MKSTSLFLCSLLLLLVTGAIGRKTKEKYSQSEE--VVSESFASGPSSGSSDE-------LVRDKPYGPKVSG----GSFGEEASEEI-----SSR-RSKHISRSSGGSNMEGESSYAKKKRS-RFAQDVLN 111

Svs4_Mouse MNSTSLFLFSLLLLLVTGAIGKKTKEKFLQSEE--TVRESFSMGSRGHMS--------RSSEPEVFVR-----PQ-DSIGDEASEEMSSSSSSRR-RSKIISSSSDGSNMEGESSYSKRKKS-RFSQDALE 113

Svs5_UGMBMR MKSTSFFLLSLFLVLVTQAVGRRPRGKFQKSFEDFSSSESSEMTMMGKDP------EEHSSSEKTWSRYHSEGPQ-DSE---------EDSEDSGS---SNYRA-KHSKEGGESAYRRRQRV-RFEQDVDK 110

Svs4_UGMBMR MKSTSFFLLSLFLVLVTQAAGRKTREKFRQSEEI-TSTELFESGIGDKGPGGNSAEE-FSILEKSRTVHHSNDNPKGMYSEEIYEETRRKRKDPGSG----FDKATNPLEEGERAYKKRKHSNLFSQDMNK 125

Svs6_UGMBMR MKSTSFLLLSLFLVLVTQAAGRRPREKFQQSSED-VSSESFEVHMLDKGPGLNSAEEEYSITEKTRTGYRSEKPK-SMYSEEIYEETLHKKNKDR--VQGNYKAKRFQDEESESSYRNRKIKPLFTGYE-- 125

Svs5_Rat MSPTGFFLLTVLLVLVTEAASRGPREKFSQSAED-PYSENMNLKILASGRGSSSTFGAYSRSENSRSNFKSKSPS-SITREKVNEES--RSEMSSTSSHFGLKMRRSHGGGEMNPFETKVKTRITRK---- 123

Svs5_Mouse MSPTSFFLLTLLLVLVTE--ARGARERFSQSAED-PSSSHMGIKIRAGGSGSGSAMEEYSVSENSWSNFKSKHPS-SISSESFHEESSSSSEMSSSGGHFGLKMRGSQAGGGMSSFKTRVKSRILK----- 122

Svs6_Rat MSPTRFFLLTVLLVLVTEAAARRPREKFSQSAED-FSSESSE----------------------------AKIPK-SVIHEDVYEEKKFTRDM--ASSDDGDDKRSLSAGEIERSFTRKKDKPRFAQEMNE 99

Svs6_Mouse MSPTSFFLLTMLLVLVTETAAKRPRERFSQAIEE-FSSESSE----------------------------ANSPK-SIVHEEVYEEKKFKRNM--VNGEDGEDSKRASAGEIERSYLRKKEKQQFAQEMDK 99

Svs5_Hamster MNPTSFFLLTLLLVLVTEPAVGRPRERFSQDT----SSEDLEVKVLGSSGGSSSIQDEYSHSESSWSSFKSKSPK-TTFTEEIYEERREK----PGDGDLSF-KTRGSQDEGMSSYKSRTKTRITGK---- 117

Svs5_Deer_mouse MNPTGFFLLTLLLVLVTETASRKTREKFSQASDD-NIDLKAL----GGSGGSSSSHEEYSSSEGSWSSFKSRSLEPTTFNLEAYDERKHKHTA--DNGNLGY-KTRGSQREDMSSFKSRMKTRIAGK---- 119

Svs6_Deer_mouse MNPTGFFLLTLLLVLVTETASRKTREKFSQASDD-NIDLKAL----GGSGGSSSSHEEYSSSEGSWSSFKSKKPK-SIVTEEVYEERRHKQRS--GDSGDGD-RSGESAGEME-SFTRRKAKQRFGQEMNK 121

Svs6_Hamster MNPTSFFLLTLLLVLVTEPAVGRPRERFSQTSEDQSSSETSEITATGGSGGSTTTHHEYSRSESSWSSFKPKSPQ-GSLTEEVYEERKYK-HT--GNSGAGD-KMSLSAGELERSYTGRKERQRFAQEIGK 126

Svs6_Vole MNPTSFFLLTLLLVLVTEAAARGPREKFSQSSEE-HSSEISEV--GGSSGGSSSANEEYSRSESSWSSFKSKNPK-SGFSEEG---------------SDGD-RASESAGEIERSYTRRKEKQRFGQEVAK 201

Tandem repeat region in Vole Svs6

SWSSFKSKNPKSGFSEEGSWSSFKSKNPKNGFSEEGSWSSFKSKNPKSSVSEEGSWSSFKSKNPKSSVSEEGSWSSFKSKNPKSSVSEEGSWSSFKSKSAKSSVGEEG
