# Supplementary material for: Identification of the major rabbit and guinea pig semen coagulum proteins and description of the diversity of the REST gene locus in the mammalian clade Glires
Source: PLoS One. 2020 Oct 14;15(10):e0240607. doi: 10.1371/journal.pone.0240607 (PMC7556508; doi:10.1371/journal.pone.0240607)
Supplement: S27 Fig — The 3’ end of human PI3 was aligned with homologous parts in Svp5. Translated nucleotides are highlighted in green and the stop codons in red. The known 3’ non-translated NT in PI3 and guinea pig Svp5 are highlighted in grey. As can be seen, the spice donor site is mutated in the guinea pig and the degu, and the spice acceptor site in the degu. (DOCX) [file pone.0240607.s029.docx]

Guinea pig Svp5 GGGATGGAGTGTTTGATCCCCGAGTGAGGCAAGCACTAGCTGGAAGAAAGGGGGACCCCTGCCAATACAGAAGAAGGTTGAGTGGTGGGGAGTGATGTCAGGGTGGTGGAAAGAAGGAAA

Chinchilla Svp5 GGGATGGAGTGCTTGATCCCCGAGTGAGGTGAGCACAGGCTGGAAGAAGAGGGGACCCCTACCAATACAGAAGAGGGTTGAGTGGTGGGGAGTAATGTCAGGGTGATGGAAAAGAGGAAA

Degu Svp5 GGGATGGAGTGTTTAATCCCTGAGTGAGTTGAGCACTAGTTGGAAGAAGAGGGGACCTCTGCCAATACAGAAGGGGGTTGAGTAATGGGAAGTAATGTCAGGGTGGTGGAAAGGAGAAAA

Human PI3 GGGATGGCCTGTTTCGTTCCCCAGTGAGGTGAGCACTAGCTGGAGAACGAGGAGACCCCTGAAGACACAAAAGAAGGCTGAGCGGTGGGGAAGCATCCCAGGTTGGTGGGAGGGAGGTTG

Guinea pig Svp5 TGGGGGAGTTGCTGGTCAGACTGAGAGACTGAGGGGTCTCAGAGGCCATGCGCAAGATGCAGA-GGGGATGTCCCTATCTGTGTACTAGGTCTGAGTGCTTTGACCTGCTGAGTCATATC

Chinchilla Svp5 TGGGG-AGTTGTTGGACAGACTGAGAGACTGAGGGGTCTCACAGGCCATAAGCAAGGTGCAGA-GAGGATGTCCTTACCTCTGCACTAGGCCTGAGTGCTTTGACCTGCTGAGTCATATC

Degu Svp5 TGGGG-AGTTGTTGGACAGACTGAGAGACTGAGGGGTCTCA--GGCCATAAGCAAAGTGCAGA-GAGGATGTCCCCATCCCTGCACTAGGTCTGAGTTCTTTGACTTGCTGAGTCATATC

Human PI3 TGGG--AGGTGACAGAAAGACTGGGAGACTGAGGGGTCTGAGAGGCTATAACCAGAGTGCCTAGAAGGATGATCTGTCTTCCTCACTGCCTCTGAGTGCTTTGATGTGCTGACTCTCACC

Guinea pig Svp5 TCTGATTCTCTTCTCATCCACCAGGTAAAACCTGTCCTTACTGCACCTGTCCTGGGCCTATGTCTGCAGGCCTTTCTGTAGTCCCTAGTAGCACTTAGTAGTCTTAGTAGCACTACCCTT

Chinchilla Svp5 TCTGATTCTCTTCCCTTCCACCAGGTAAAACCTTTCCTCACTGCACCTGTCCTGGGCCCAGGCATGCAGGCCTTTCTCTAGTCCC-------------AGCTCTTAGTAGCACCACCCTT

Degu Svp5 TCTGATTCTCTTCTCTCCCACCGGGTAAAACCTGTCCTCAGTGTACCTGTCCTCGGCCCAGGCCTGCAGACTTCTCTCTAGTGCC-------------AACTCTTAGTAGCACAATCTTT

Human PI3 TCTGATACTCTTCTCTTCCACAGAGGGAG-CCGGTCCTTGCTGCACCTGTGCCGTCCCCAGAGCTACAGGCCCCA-TCTGGTCCT-------------AAGTCCCTGCTGCCCTTCCCCT

Guinea pig Svp5 TCC--CAGTGCCCATTGTTCCTCCTGATCTGGATGCCCAGTCTTGGGACTGCCTTTGTCTTCTACTTTCCAATAAAAAAGAAAGAAAAAA-----CCCTCCTGCTCCATTTGTTTCTGGC

Chinchilla Svp5 TGCCACAGTGCCCATTCATCCTCTTGTTCTGAATGCCCAGTCCTGGGACTGCCTTTGTCTTCTACTTTCCAATAAAAAGAAAAAACA--------CCCTTCTGCTCCATTTGTTTCTGGC

Degu Svp5 TCCCACAGTGCCCATTATTCCTTTTGTTCTGGATGCCCAGTCCTGGAACTGTCTTTATCTTCTACTTTCCAATAAAAAGAAAAAAAAAAAAAAACCTCTTTTGCTCCATTTGTTTCTAGC

Human PI3 TCCCACACTGTCCATTCTTCCTCCCATTCAGGATGCCCACGGCTGGAGCTGCCTCTCTCATCCACTTTCCAATAAAGAG---------------TTCCTTCTGCTCCACTTGTTTCTGGT
