# Supplementary material for: Identification of the major rabbit and guinea pig semen coagulum proteins and description of the diversity of the REST gene locus in the mammalian clade Glires
Source: PLoS One. 2020 Oct 14;15(10):e0240607. doi: 10.1371/journal.pone.0240607 (PMC7556508; doi:10.1371/journal.pone.0240607)
Supplement: S28 Fig — The aligned primary structure of Svp2 precursors are shown with star symbols (*) indicating residues conserved in all of the analyzed species. Underlined residues are encoded by SPCE. (DOCX) [file pone.0240607.s030.docx]

Guinea pig Svp2 MKSTLFFILALLLILENQASGRRLRGSARAQDPVVSRVWHKEEVEESESSRGQDFDKRRFWEKDDPTGEH 70

Damaraland mole-rat Svp2 MKSTVFFILALLLILESQAAGKSLRGPARAQDPLINRVWHKE-VEETESSRGQDFDKHRFWEKDGPTGER 69

Naked mole-rat Svp2 MKSTIFFILALLLILENQVSGRRLRGPSRAQDPLISHVWHKE-VEESESSRGQDFDKLHFWEKDDPTGER 69

Degu Svp2 MKSTVFFILALLLVLEHQAYGRRLRGPIRSQDPLISRVWHKE-VEETESSRGQDMDKRRFWEKGDPTGER 69

Chinchilla Svp2 MKSTVFFILALLLILE--ASGRRLRAPVRSQDPLISRVWHKE-VEETESSRGQDFDKRRFWEKDDPTGER 67

**** ******** ** * ** * *** ***** *** ******* ** **** ****

Guinea pig Svp2 VSVRHEHLEKSHIRFKEDSIDDSGSAGGLNPLKGHLRLKRHDAMEELVSVEDQALANGADPGKSNMQRV 139

Damaraland mole-rat Svp2 VSVRREHLEKSHIRFKEDSTDDSGSVGGLNSLKGHLRLKRHDSMEELASVEERDSANGVDPGKS---PI 135

Naked mole-rat Svp2 VSVRHEHLEKSHIRFKEDSMDDSGSAGDLDPLKGHLQLKQHDSMEELVSVEARDSANGIDPGKSHIQCV 138

Degu Svp2 FSVRHEHLEKSHVRFKEDSMDDSGSVGGLDPLKGHLRLKRHDSMEDLVSVEDQNSAN-VDPGK----HI 133

Chinchilla Svp2 VSVRHEHLEKSHIRFKEDSVDDAGSVGGLDPLKGHLRLKRHDSMEELVSVEDQDSANVGDPAKSHSQHV 137

*** ******* ****** ** ** * * ***** ** ** ** * *** ** ** *
