# Supplementary material for: Identification of the major rabbit and guinea pig semen coagulum proteins and description of the diversity of the REST gene locus in the mammalian clade Glires
Source: PLoS One. 2020 Oct 14;15(10):e0240607. doi: 10.1371/journal.pone.0240607 (PMC7556508; doi:10.1371/journal.pone.0240607)
Supplement: S29 Fig — Sequence alignment of translations from hystricomorph Svp1, including potential pseudogenes, are shown. Damaraland and naked depict the mole-rats. Star symbols (*) highlighted in red indicate stop codons, whereas the same symbol written below the aligned sequences indicates fully conserved residues. The underlining at the amino-terminals depict residues encoded by SPCE and the underlining central in the guinea pig sequence shows the location of the processing site that generates Svp3/4 from the N-terminal part of the precursor and Svp1 from the C-terminal part. The residues highlighted in yellow show conserved residues, which suggest that the tandem repeats in Svp3/4 and Svp1 are homologous and probably have evolved following an initial duplication of a peptide encompassing Trp, Lys, and Gln. (DOCX) [file pone.0240607.s031.docx]

Damaraland Svp1 MKSTVFFILSLLLILENQAAGRRLHDSVGSQDPLLSRAWAKSQDMEESVSGRGLNVEGSHISDKGEESMGEH-----EEFEKGHIWHNVEDPEGEHVSVRREHLEKS-VRPNEEDPKGER 114

Guinea pig Svp1 MKSTIFFILSLLLMLENQAAGRRLSGSAGAQDPVISHVWAKSQDMEEAVSGSGLTAEGGRGSDR-EESVGERVSLRQEEFEKGHIRSSVEEPEGEHVSVRREHLEKSHIRHNVEEPEGER 119

Degu Svp1 MKSTVFFILSLLLLLENQAAGRRLHGSVGAQDPLISRVWAKSQDMEESVSGPGLGAEGGRVSDR-EDSLGEHISVRHEEFEKGHIRHNTEEPEGEHVSVRREHLEKSHLRHNAEEPERER 119

Chinchilla Svp1 MKSTVFFILSLLLILENRAAEGRLRGSAGAQDPLISRVWAKSQDMEESVSGPGLTAEGGHISDKGEASMGEHISLRHEEFEKGHIRHNVEEPEGERVSVRHEHLEKSHVRHNVEEPEGER 120

Naked Svp1 MKSTIFFIFPLLLILENQAARRRLRGSVGSQYPLLSHVWAKS*DMEESVLGRGLDVEGGRTPDKGEGSMGEHDSVRHEEFEKGHIWHNVEDPEGERISLRHEHIEKSHVQDNVEDPEG*S 120

**** *** *** *** ** ** * * * * * **** **** * * ** ** * * * ** ******** * **** * * ** *** * * *

Damaraland Svp1 ---------- ---------------------------VSVRHEHLKKSHIRHSAGNPTRKRISLRHEELEKGHIRYKSEDPEGERVSLRHKSIEKTHK 175

Guinea pig Svp1 ---------- ---------------------------VSVRREHLEKSHIRHSAEEPEGER------------------------VSVRHERIEKTHK 156

Degu Svp1 ---------- ---------------------------LSVRHEHLEKSHVRHSGEEPMGERVSLRHEEMEKGHIRYKTDDPVGERVSVRHEHIEKTHK 180

Chinchilla Svp1 VSVRHEHLEK <-- sequence gap --> GERVSVRHEHLEKSHVRHNVEEPEGERVSVRHEHLEKSHVRHNAEDPMGERVSLRHEEMEKGHVRYKAEDPAGERVSLRHEHIEKTHK 218

Naked Svp1 ---------- ---VSLRYEHVEKGHVQHNVEDPKGEPVSVRCEHLEKSDVQHSADDPTRECISLRHEELEKGHIRYKAEDPEGEQVSFRHECIEKTHK 205

*** *** ** * * ** ** ******

Damaraland Svp1 RFRDDSMEDS--TESLDRTMKGRIRFK*QEPIVALVSMEGQDTVKDNLWVKSQGASEERFSVKGQDSVQGHLQMKGQSSMAERSSVTGQDSLKSRLQMKGQDSVADRFSITGQDSLKSRL 293

Guinea pig Svp1 RFHDDSVEESDSASSVDHRKKGHIRFKRQDPIAALAAIEGQDAVKDSLWVKGQASSEERFSVKGQDLVKGHLQ------------------------MKGQSSLAERFSVTGQDSVKGRL 252

Degu Svp1 RFNDNSMEESDSAESYDNRMKGHIRFKRQDPMAALVSIDSQDAMKDSLWVKTQGVSEERFSIKGQESLKSQLQMKGQSSMGERFS-TGQDSLTSRLQMKGQSSMGERFSVTGQDSLKGRL 299

Chinchilla Svp1 RFRDDSVEESGSAETFDRRMKGHIRFKRQDPMAALVSIDSQASMKD--WVKGQGVSEERFSVKGQDLVKAHLQMKGQSSMAERFSVTGQDSVKGRLQVKGQDTLAERFSITGQESMKGRL 336

Naked Svp1 RFRDDSMEDSDSAKSLDCPMKGHICFK*QEPMVAVVSMEGQDAMKDSLWVKSQGLSEEQFSVKGQDSLQGNLQMKGQSSMVERFSVTG*DSLKGR------------------------- 300

** * * * * * ** * ** * * * * ** *** * *** ** *** **

Damaraland Svp1 QMKGQDSMAEGFSLAGQDSLKGCLQLKGQSSMAE*FSVTGQDSLKGC----------------------------------------------------------------- 340

Guinea pig Svp1 QMKGQDTLAERFSMTGQDSVKSRLQMKGQDSLSERFSMTGQDSVKGRLQMKGQSSLAERFSVTGQDSVKGRLQMKGKDTLAERFSVTGQDSVKGR----------------- 347

Degu Svp1 QLQGQDTMAERFSVSSQDSVKGRLKLKGQDTLAERFSVSGQDSVKGRLQIKGQDTLAERFSVSGQDSVKGHLQLKGQDTLAERFSVTSQGSVKGH----------------- 394

Chinchilla Svp1 QVQGQDSLTERFSVSGQDSVKGRLQMKGQSSMAERFSVTGQDSVKGRLQIKGQDSLTERFSVTGQESVKGRLQIKGQDSLAERFSVSGQDSVKGRLQIKGQDSLTERFSVSG <------ 448

Naked Svp1 ---------------------------------------------------------------------------------------------------------------- 348

Damaraland Svp1 ---------------------------LQIKGQDLMEERFSVAGQDSVKDLARIKGQDAIQSGFSIKSQGSVKGLTGDI 392

Guinea pig Svp1 ---------------------------LQMKGHDLLEERFSVSGQDSVKGLARIKGQESVQSGFSVKGQGSLKGL---I 396

Degu Svp1 ---------------------------LQLKGQDLMEERFSAAGQSSVKGFGRVKGQESFQSGFAVKGQGSVKSLTGDI 446

Chinchilla Svp1 sequence gap --> VSGQDSVKGRLQIKGQDSLTERFSVSGQDSVKGLGRIKGQDTIQSGFSIKGQASVKGLIGDI 510

Naked Svp1 ---------------------------LQMKGQSSMAERFSVTGQDSVKGLARVKGQDVIQLGFSMKGQGSVKGLIRDI 352

** ** **** ** **** * *** * ** * * * * * *
