# Supplementary material for: Identification of the major rabbit and guinea pig semen coagulum proteins and description of the diversity of the REST gene locus in the mammalian clade Glires
Source: PLoS One. 2020 Oct 14;15(10):e0240607. doi: 10.1371/journal.pone.0240607 (PMC7556508; doi:10.1371/journal.pone.0240607)
Supplement: S30 Fig — Alignments of the highly conserved termini in hystricomorp Svp5 is shown. Conserved residues are indicated by star symbols (*). (DOCX) [file pone.0240607.s032.docx]

Guinea pig Svp5 MKPTVFLILSLLLIWVKQASGKKLLVAVKGQETVQGQVWTTGQDPMEEEFIVQRKDPMIGSIWVNGQGFVEEAVSVKGLSSMKGRMRVKGQALIGKTVSVKGLHPVKGRMKVKGQTLMGK 120

Chinchilla Svp5 MKSTVFLILSLLLIRVKQASGKKLLVAVKGQEASQGQVWTTGQEPVEEEFVVQHKDPMIGHIWVDGQDFVEEAVSLKGLSSVKGRMRVKGQALIGEAVSVKSLSPVKGHMKVKGQTLMGK 120

Degu Svp5 MKSTVFLILSLLLIRVKHAAGKKLLVAVKGQETVQGHVWTTQQDPVEEQFLVQQKDPMIDHIWVNGQDFVEEEDSVKGLS------------------------PVKGHMKVKGQTLMGK 114

** *********** ** * ************ ** **** * * ** * ** ***** *** ** **** * **** **** ***********

Guinea pig Svp5 AVSVKGHGSLKSRMQVKGQDLMGEEFLVQGNDPVISHIWVNQEDFVENPVSVKSLGMVKGRGYLKGHGYLKGQGSLKGQLQIKGQDSMEEEITVKGLQPVKGPIQVKGQDLMGEEFLVQH 240

Chinchilla Svp5 AVSIKGHGSLKSRMQVKGQDLMGEEFLVQGTDPVISHIWVNGQDFVEKPVSVKRLGVVKGQ------GYLKGQGSLKGHLQIRGQDSMEEVVSVKALQPVKGHMQVKGQDLTGEQFLVQG 234

Degu Svp5 AVSIKGHGSLKSRMQVKGQDLMGEELLVQGNDPVISHIWVNRQDFVEKPVSVKSLGVIKGQ------GYLKGKGSLKNQLQIKGQDSMEEIVTVKGLHPVKGTIQIKGQDLTGEEFLVQS 210

*** ********************* **** ********** **** ***** ** *** ***** **** *** ******* * *** * ***** ** ****

Guinea pig Svp5 TDPMISHIWVDGQDFMEEEVSDKGLSPVKGHM <----- 1120 AA repeat region -----> KGLGPVKSHTQVKGQNFQDAAVSVKTLGSIKRQGSSRSQSSLKGPIQ 1439

Chinchilla Svp5 KDPMISHIWVDGQDFVEEAVLVKGLSPVKGHM <----- >631 AA repeat region -----> KGLGAVKGRMQVKGQDFQDAAVTVKAPGSVKGRGSSRGRGSLKGPMQ (554)

Degu Svp5 TDPVISHIWVNGQDVVEEAVSVKGVGAVKGRG <----- >1638 AA repeat region -----> KGVGSLKSHMQVKGQDLLGETVSVKGLSPVKGQGSVRGLSSPRGPTQ 1929

** ****** *** ** ** *** ** * * ***** * ** K** * * ** * *

Guinea pig Svp5 IKGQDSTKEAVLVQGLSSPKSHMQIH-ENAMEDTVLIKGQDRMKRHRQFKGQDHMEEHAAFKGQGMFKRRSFSKPGSCPDITGQCTQTS-DSKCGSDVECPGTKKCCVGMCGGMECLIPE 1557

Chinchilla Svp5 IKGQDSMKEAVSAQGLSSVKNHMQSKGQNTMEKTVLIKDQDLMKSHMQFKGQNHMEEQEAFKGQETFKGQSFSKPGTCPDIVAQCTYTSYPNKCGSDVECPGTMKCCLGMC-GMECLIPE (673)

Degu Svp5 IKGQDSTKVAILAQGLSSLQNRMQLNGQTGMEETVLTKGQDLMKSHTQFKGQNHMQEQEAFKGQETFKGQSFSKPGSCPEIVAQCTYASNPNMCGSDVECPGTKKCCLGIC-GMECLIPE 2048

****** * * ***** ** ** *** * ** ** * ***** ** * **** ** ****** * * *** * *********** *** * * *******
