# Supplementary material for: Identification of the major rabbit and guinea pig semen coagulum proteins and description of the diversity of the REST gene locus in the mammalian clade Glires
Source: PLoS One. 2020 Oct 14;15(10):e0240607. doi: 10.1371/journal.pone.0240607 (PMC7556508; doi:10.1371/journal.pone.0240607)
Supplement: S31 Fig — Nucleotide sequences of the genes, tentatively denoted Svsc1-Svsc4, are given with translated nucleotides highlighted in green, and non-translated in grey. The TATA box in the upstream promoter region is doubly underlined and translations in one-letter code are written above the coding nucleotides. Two poly-Gln tracts in Svsc3 are highlighted with thick underlining. (DOCX) [file pone.0240607.s033.docx]

Kangaroo rat *Svsc1*

TAATTTTAAAAAAGGGCAGTGTCCTTTGTAACTTCAGCCAAGCTCCATGGCATATCGAGTCAAGAAAGGTATAAA

M K S T I F F I L S

TGAGGAGCTCAGCTTGGCTTTCAGACAAGGTACTTCCTGACAAGATGAAGTCCACCATCTTCTTCATCCTGTCCC

L L F I L Q N Q A S G G G F H

TGCTCTTCATTCTGCAGAACCAAGCATCTGGGGGTGGATTCCATGGTGAGTGGAGGGTGACATCTAGGGAGAAAG

TACTTAAGGGAAAACTCTTCCAAGAATACTCTAGAAATTAGCAGAATATCCTTAGAGCCACTAGGGGAGTCTTGT

CCCTGATAAGATTTTACCTTCCTTCACTGTTACGATAGCTCTTGCATGAACATAAGTGAGGAGATGTTAAAAAGG

G A

TAGGGGGTAACAACTGTCAGGGAACTTAAAGAGATCGTGTAGCTCAGACATTCTCTCATTTCAATTACCAGGTGC

G Q N P L E G L K S K F P N R P R N H R H F G H H

TGGGCAAAATCCTTTGGAAGGCTTAAAATCCAAATTTCCAAATAGACCAAGAAACCACCGGCATTTTGGCCATCA

K R I E S E M G S V G G G E T R G V D G G T E M K

CAAGCGCATTGAATCTGAAATGGGGTCTGTAGGTGGAGGTGAGACAAGGGGAGTTGATGGTGGGACTGAAATGAA

Y A V S V F A T E D R T K N R P N E D L N E S F G

ATATGCAGTTTCTGTCTTCGCCACCGAAGACAGAACTAAAAACAGACCCAATGAGGATTTAAATGAAAGTTTCGG

V N R G H R R E H F E S F H K R K E R N S D G G F

AGTAAACCGGGGTCACCGCAGGGAACACTTTGAAAGTTTCCATAAACGCAAGGAACGAAACAGTGATGGAGGATT

G F S K K K T V H I Q H E H M Q *

TGGATTTTCAAAGAAAAAGACAGTCCACATTCAACACGAACACATGCAGTAATACAGTCACTGGCCAAGTGAAGA

CCTGGACCAATTTTGAGGTAAGCGTTTTGGTTCTGACCACATAAGTGTGATATGTACCTCAGTGCTTAGAACTTT

GTGGGCCCATCGGGAAATGGATACCCAGTGCCCACACTAATGCAAGTGCAGTGTTACCAATGTTAGGAAGTTGAG

CAGCAAATCCTGGTAAGGAGAGCACATGCACATGATAGAGACAGAGAAAGCTACCTGGGCTATCACTGGGTGCTG

AATTCCTATCCTATCTTTCTTTCTTTCCTTCTTCCTTTCTTTCTTTCACTCGTTCTTCCTTTCTTTCTATTTTTT

TGTGTCGGTCTTGGGGCTTGAACTCTGGGTCTGGGCGCTAGCCTGAGCTCTTCAGCTCAAGGTGAACGCTCCACC

ACTTGAGCCACCACATCACTTCCGATTTTCTGGTGGTTAATTGGAGATGAGTCTCACAGACTTTCCTACCTGGGC

TGGCTCTGAACCATGATCCTCAAATCTCAGCCTCCTTTGTAGCTAGGATTACAGGCGTGAGCCACTGGTGCCCAG

CCCAGGCTGGCCTTGAACCACCATCTTCAGATCTCAGCCTCCTGAGTAGCTAGGATTACAGGTGTGAGCCACCAG

TTCCTGGCTCTATCCTGAATGATTCTTTAAAGAGGCTTTATTCAGCACAAAGCTACCAGTTGAAAGTATTTTGAG

CATCTTCCCTGAATATAAGCATATTTATGCTTCTCATGGCATCCTGTCATTCTTACAGTTTTGAATATTGAAGCT

CAATTCCTTGCTTATATTGGTATGAAGTCTTTATTCTCTGTCTTTATACTCATGAATCCCTGTCTCTTTCAGAGA

TCACATGTTAAACAATCTTTCCCATCAGAGCTCAGCAAAGAGATGTGATACAGGGGAGATGAAGAGTAGGTGCAA

GAGATAGTGATGGTCCACAGGGACCAGTGAAAAGTTAGCCATATCAAGAGGGAAATTTAAAGTCCACGGAGGAAA

AAAAACAAAAAGACATGCCCTGCATGTTTGCAGTAAATAACACATCAATTAAAGGAAAAGCAAGCAACAAAATCA

ATGGACAAAGCTAGTATCCTCATTTCCAGTGTCTGGTACATTAGAGGGCAAAATAATAGAGGCCCATCTCCTCTC

CCCACCCGTCCCCACTCACTGCCCTCATGGCTCAGCTTGATCCTCTGCCTCCCTAGGAACCACCTCACTTGAGTG

AAGTCTGTGATGTGTTCGAGATGAAGACTTCCATGTGGTCCCACAGCCTTGGTCCATGGATGACACCTTGTACTC

TCATTTGCTTTCCTTGAGCTTTGGAAAACTGAAGATTCCTCATATACTTGCTTTCAAATAAAAAGATCACTTTCT

GCATCATTTGCTTCTGACTCCTGAGACCTTATTTCCTTGAAGTTTAGGGGATAAGGAGATAATTCTTGTGCTAGA
