# Supplementary material for: Identification of the major rabbit and guinea pig semen coagulum proteins and description of the diversity of the REST gene locus in the mammalian clade Glires
Source: PLoS One. 2020 Oct 14;15(10):e0240607. doi: 10.1371/journal.pone.0240607 (PMC7556508; doi:10.1371/journal.pone.0240607)
Supplement: S32 Fig — Nucleotide sequences of the genes, tentatively denoted Svsc1-Svsc4, are given with translated nucleotides highlighted in green, and non-translated in grey. The TATA box in the upstream promoter region is doubly underlined and translations in one-letter code are written above the coding nucleotides. Two poly-Gln tracts in Svsc3 are highlighted with thick underlining. (DOCX) [file pone.0240607.s034.docx]

Kangaroo rat *Svsc2*

AAGCTGGCATTTACTGGTAATGGCAGTGTCTGTGGTGACTTTAGCCACACCCCTAGCACACCCAGTTGAATAAGA

M K S T T F F

TATAAATGGGATGGATACACAACTCAGCTCTCAGCAAAGATCCTTCCCAGCAAGATGAAGTCCACCACTTTCTTC

I I S L L L I L Q E Q I A V G I G Y H

ATCATTTCTCTGCTTCTCATTCTGCAGGAGCAAATAGCAGTTGGAATTGGATATCATGGTGAGTGGGGAAAGTCT

GTTGGGGGAAAGATAACTAGGGGAGAAAGGTGTCTTGTAAGGGAACTCTAAAAGTGACTAGAACCTTCTTCACTC

AGAGCTTCTAATGCCTGGTTAAAAAAAAAAAAACACCAAGTCTCCTCCACACATCCCTTATGAGAACATCAGAGA

GCCGAGGAAGAACAGGGTTGGGTTACAAGTTGCAAGGGGTCATGAGTGGCAAGGGGTCCCTGGAGATGGTATAAC

G S P P G Q L S N E I P A F P K R

TTGGACGTCTTATTGTCATTTACTAGGCAGTCCTCCAGGCCAGTTGTCAAATGAAATACCTGCATTTCCAAAGAG

P R G S R G F G H H R T V H V T E D S S L S Q P E

GCCAAGAGGTTCACGGGGTTTCGGACATCATCGCACTGTGCATGTTACAGAAGATAGTTCTCTAAGCCAACCTGA

P K V G D S D S D T K Y T L K E K E T I I S E E H

ACCAAAAGTTGGTGACAGTGATTCTGATACAAAATACACACTTAAAGAAAAAGAAACCATTATCTCCGAGGAACA

R S E N K P D S F S S G D S L E N K V E S R V H R

CAGATCTGAAAACAAACCTGACAGCTTTTCTTCCGGTGACAGTCTCGAGAATAAGGTAGAAAGTCGTGTACACAG

H F H E R R H K T E K E D Q G G Y E F S K K R T I

ACATTTCCATGAAAGACGCCACAAAACTGAAAAGGAAGATCAAGGCGGCTATGAATTTTCCAAGAAAAGGACCAT

Q Y Q H E H N E *

CCAGTATCAACATGAACACAATGAATAATACACCCACTGACCAACTGAATACCTGGACTGATACCAAGGTAAGAT

TTGTTTTAACCAAATAAGAATGATACTAAATCCAGTGTTTAAAAATAGTACATGAGGACTTTCAAGGCCCATTAT

GGGATGGATACCCACTGTCCACATCAGCAGAAGTGCTGTTACAAACTTGGAGAGCATGAGTGCCACTCGCTGGTA

AGAAGAACACCTGCCAGAGGCACAAAACTATCCCTATCACAGAACCCTAAATGTATATCCCCAGTAAGCATTTTA

AGGGGCACTTACCTAACAGTAAATTCTTTTCTGAGCGTACACCTAAGCATATTTCTGAATTTGAAATATAACCAT

ATTCTTTGGAATATTGGAATTTAGTTTTTTGCTTATATTGGTATTTTCTTTCTCACTAGGAGTTATGGGGAAACC

CCTGTAATCCCAGCTCCTTGGGAGATAGAGGCAGGAGAATCACAAGTTAGAGGCCAACTTGGACAAGTTACTGAG

ATCATGTCTCAAAAATAAAATAACAACCAAAAAAAAGGGACTGGGTGCAGCCTCAGTGGTAGGATTCCCAATACC

ATATATATATATATATATATATATATATATATATATATATAACATACCATATATATAACATACCATATATATAAC

ATACCATATATATAACATACCATATATATTACATACCATATATATTACATACCATATATATTACATACCATATAC

ATTACATACCATATATATACATACATATTCTTTATCTCGACACCAACGAGTGTACTTGCTTCTCTGTTGGGGTAC

ATCTATCCTCTTTCAGAGACTGCTGGCCCAAGGTTGAGCTGCCCTTCCTTCAGAATATAAGAAGAAGACAATCAC

AAATAAAGATCCCTGTGTGTGATGCAGGAGAGATGAAAAGTGGGCACAAGAAGTGTTGAGAAATATCAGGTATAA

GAGGGACATTTGCAGTGTCATGACAGAAATGTCAAGCCCAAGGAGAGAAAATGATGTGCCCTGCACATTTGTACT

AATAACACATCGGTTAGAGGAAAAGTAAGCAACAAAATCAATGGACAAAGCTAGTATTGTCATTGTCAGTGGCTG

GTACAGTAGGGAGTGTGCCTCCTATCCCCACCCACCACCCTTCGCTGCCTCACTTCTCAACTTGCCCTCTGCCTC

CCCAGGAGTCACCTAACCTGAGTGAAGACTGTGATGTGTTCCAGATGAAGACTTCCACGTGGTCCCGCAGCCTCG

GTCCATGGATGACACCTTGTGCTCACACCTGCTTGTCTTGGGGTTTCTAAACCTGGAGATTCTTCAAACACCTGC

TTTCAAATAAAAAGATCACTTTCTGCATCATTTACTCTTGACTACTGAGATCTTATTTCCTTGAAGTTTAGGGAA
