# Supplementary material for: Identification of the major rabbit and guinea pig semen coagulum proteins and description of the diversity of the REST gene locus in the mammalian clade Glires
Source: PLoS One. 2020 Oct 14;15(10):e0240607. doi: 10.1371/journal.pone.0240607 (PMC7556508; doi:10.1371/journal.pone.0240607)
Supplement: S33 Fig — Nucleotide sequences of the genes, tentatively denoted Svsc1-Svsc4, are given with translated nucleotides highlighted in green, and non-translated in grey. The TATA box in the upstream promoter region is doubly underlined and translatations in one-letter code are written above the coding nucleotides. Two poly-Gln tracts in Svsc3 are highlighted with thick underlining. (DOCX) [file pone.0240607.s035.docx]

Kangaroo rat *Svsc3*

GCGCCTTCTCGCACCAGCCACACCCGTGGCACACACAGTCAAAGAAGATATAAACCAGAGGCTCCACTCAGCTCT

M K S P I F I L S L L L I L E K Q A

CAGCTGACATCTTCCTAGCAAGATGAAGTCCCCCATCTTCATCCTTTCTCTGCTCCTCATTCTGGAGAAGCAAGC

A G M P F Y

CGCTGGTATGCCATTCTATGGTGAGTGGGAAAGGCTGGCTAGGGGGAAAAAGTCCTTCAGGGAGAATGTCTTCTA

AGGTTACTTTGGTCGGGGGGTGGCAGTTAGCAGAAGTACCTTTAGGCATACCTTCTTCTTCACAATGAACTGAGT

CTTCCCCACCAAAATCCAAATCCCTTGGGTAATCATCAGTGGTTCCATAAAAAAGGGTCTAGATAAGGCTTGGAA

G Q T K S Q F P D

GGCAGCATTAGAGATCATGTAACTCACACATTCTTATTATCAATTACCAGGCCAGACAAAAAGCCAGTTTCCAGA

R S Y E H L L T Q Q I Q Q A L Q Q Q T Q H H H E P

TAGATCCTATGAGCATCTACTTACACAACAGATTCAACAGGCACTTCAACAACAGACTCAACACCACCATGAACC

T A T K G V L A A E G I V T K T K S Q V Q T K D F

AACAGCCACAAAAGGAGTTTTAGCTGCAGAAGGAATTGTAACCAAAACAAAATCCCAGGTACAGACCAAAGATTT

G M P Q Q Q Q Q Q Q Q Q Q Q Q M T Q Q T I A R E D

TGGCATGCCGCAACAACAACAACAACAACAACAACAACAACAACAGATGACACAGCAAACTATAGCTAGAGAAGA

T L C A A E G I K T Q Q L S K G I S S Q Q S H Q K

TACACTGTGTGCTGCAGAAGGAATCAAAACCCAACAACTATCCAAAGGCATTTCTTCCCAGCAAAGTCATCAGAA

T L V L K G E P S Q Q K T L V V K G E E S Q K T L

AACACTTGTCTTGAAAGGTGAGCCAAGTCAGCAGAAGACACTAGTTGTGAAAGGTGAGGAAAGTCAGAAGACACT

I L T G D P N Q Q K T L L V Q G A P S Q Q K T L V

TATCCTGACAGGCGACCCAAATCAGCAGAAGACACTTCTGGTGCAAGGTGCACCAAGTCAGCAGAAGACACTTGT

V T N E P S Q L K T L V V T N E P S Q Q K T L V V

TGTGACAAATGAGCCAAGTCAGTTGAAGACACTTGTTGTGACAAATGAGCCAAGTCAACAGAAGACACTTGTTGT

T N E P S Q L K T L V V Q G E P N Q L K S L M V P

GACAAATGAGCCAAGTCAGTTGAAGACACTTGTGGTGCAAGGCGAGCCAAATCAGCTGAAGTCACTTATGGTGCC

D A P S Q L K S L M V P G A P S Q L K T L M V P G

AGATGCTCCAAGTCAGCTGAAGTCACTTATGGTGCCAGGTGCGCCAAGTCAGCTGAAGACACTTATGGTGCCAGG

V P S Q L K S L I V P G A P S Q L K S L M V P G V

TGTACCAAGTCAGCTGAAGTCACTTATAGTGCCAGGTGCGCCAAGTCAGCTGAAGTCACTTATGGTGCCAGGTGT

P S Q L K S L V V P G A P S Q L K S L M V P G A P

GCCAAGTCAGCTGAAGTCACTTGTGGTGCCAGGTGCTCCAAGTCAGCTGAAGTCACTTATGGTGCCAGGTGCGCC

S Q L K S L M V P G V P S Q L K S L M V P G A P S

AAGTCAGCTGAAGTCACTTATGGTGCCAGGTGTGCCAAGTCAGCTGAAGTCACTTATGGTGCCAGGTGCTCCAAG

Q L K T L M V P G A P S Q L K T L M V Q G E P S Q

TCAGCTGAAGACACTTATGGTGCCAGGTGCGCCAAGTCAGCTGAAGACACTTATGGTGCAAGGCGAGCCAAGTCA

L K T L M V S G A P S Q L K T L M V P G A P S Q L

GCTGAAGACACTTATGGTGTCAGGTGCGCCAAGTCAGCTGAAGACACTTATGGTGCCAGGTGCTCCAAGTCAGCT

K S L V V K G M K T Q I H Q Q K E D Y G P Q A Q R

GAAGTCACTTGTGGTGAAAGGCATGAAGACACAGATCCATCAACAAAAGGAAGACTATGGCCCACAGGCTCAGAG

H V V Q L V N T K E S M L Y H G P T Q Q Q Q Q Q L

ACACGTTGTCCAACTTGTTAACACAAAAGAAAGCATGCTCTATCATGGACCAACACAACAACAACAACAACAACT

Y Q V Q Y K Q Q L H G H S D Q L K A A Y L S Q G Q

ATATCAGGTCCAATACAAACAGCAGCTACATGGCCATAGTGATCAACTAAAAGCTGCTTATCTTAGTCAAGGGCA

C R C I K G I A L K S I A *

GTGCAGATGTATAAAGGGGATAGCGTTGAAATCTATTGCCTAATACACTCATTGGCTAACTGAAGACCTGGATCA

ATATTGAGGTATGTTTTCTTAGCAAACAGGATACATACATGTTTAGAAGTAGTACATACAAAGTCCCTGGGCCCA

CAATGGGAATGTATCCACTGTGCACATCCATGGAAATGTTGTGCTATAAACTTTGGGGAAATGTATAACAGTTCC

TGGTGAGGTGGGTACTGGTCAAGATACAGAAAGCAAACTTAGCATCTTCAAGGGTCTTCTCTCTTTTTTTCATTG

GAGGGGGGCAGTTTTTGGTGGTACTAGGATTTGGACTTGTTAGACAGGCACTCTGACACTTGAACCACTCTTACC

ATTTTTTGCTTTTCTTTTTCTTTTTTGTCAATTGTGGAGCTTGAACTCTGGGCTTGGACACTATTCCTCAGCTCT

TCAGCTCAAGGCGAGTGCTCTACCACTTGAGCCACAGCACCACTTCAGGTTTTCAGGTGGTTAATTGGAGATAAA

AGTCTCATGGACTTTCCTGCCCAGGCTGGCTTTGAACCACAATGCTCAGATCTCAGCCTCCTGAGTAGCTAGGGT

GACAGGCGTGAGTCACTGGCTCCCAGCCTTTTTGCTTTTCTGTATTTTTTTGGAGTGTAGGGAGGCACCATTACC

AGAGTTTGAACTCAGTACCTTGAGCTCTCATTCAGTTTTCTTGCTCACAGCCGATGATCTGCCAATAGAGCCACA

CATCCAGCCTGGATTTTTGCTGGTTAATTGGAAGTGGGAGTCTCTCAGACTTTTCTGTCCAAGCTTGCTTTGAAC

TATGACCTTCCAGGTCTCAATCTCCTGAGTAGCTTAAATTATAGCTCTGAGCCATAGCACCCAGCTGCCTTGGTT

ATTTTTGAGATAGGCTCTTGTATTTATGCCTGGACCACTTAACTACACAATCTATTTCCACTATCCACATAGCTG

AGATGATAGGCACCGTCCACCATGCCCAGCTTTTTATTGATTGAGATGGGGTGGAGGAGTCTTTCTGTTTTTCCA

TCTGGTCTCTCTCTACAATCCTCCCAATTTCCACCTTTCAAGTCGCTAGCATTACAGGCATAGGCTACCACATCC

AGCTTTAGTGGTGTTTTGTTGCACGTTATCTATTCTAAAATTAAATGTGTACCACATATCCAATCATATTTATGA

GCTCATGTTGTAACCTTTTAAATTCGGGTGCTTTTGCTTAGTAAAGCTTAATTTCTTTTCTCACTTTGCCATGAA

GTATTAGAACAGTGAGTTCTTTGTTATGCATAGCATAGCTATTCTTGTATCTCTAGTAGAGTGCATTTATCCTCC

TTTGTAGATCCAACCATTAGCTTCTTTTTCCCTCACAATACAGGAGAGAGAGTAATACCAAAACGGTCCCTGCTT

GTGATGCAGGAGAGCTGAAAAAGGGAGGCACCAGAGGAACCACAGGTCCCAGTTGGAATATTTGCAATGTCAGGA

GAGAAATCTGGAGTCCAAGGAGGAAAAAAAAGGTGTTCCCTGCAAGTTTCCATTAATAGTCCTTCATCTGAGCAA

AGGAAAGCAACAGAAACCATGGATCCCCTGGACCTTAGGGGTCTACCACCCCAATGTGCAGGAGTTGGTCCAGAT

GGAGCTAAAAGGGACTTGGGGTCACAGCTCATGTCTTGACCACTGACTCTTCACTGGCTTCACATCACTGTGCCC

TCTTTCTTTAGGAGTCACCTGACCTGAGTGAAATCTGTGATCCAGACTCCCATGTGCCCCAAGATATTCTTCTAT

GGATGGGATCACCCGTTCATGCTTGCTTGTCTTTAACTTGGAGATTGCTCAAAATTTTGCTTTCTAATAAAGAGT

TAACTTTCTGCATCATTTGTTTTGACTCCTGAGATCTCTTGGTTCCTACAATGTCAGGGGCTTGGTGAGAATTTC
