# Supplementary material for: Identification of the major rabbit and guinea pig semen coagulum proteins and description of the diversity of the REST gene locus in the mammalian clade Glires
Source: PLoS One. 2020 Oct 14;15(10):e0240607. doi: 10.1371/journal.pone.0240607 (PMC7556508; doi:10.1371/journal.pone.0240607)
Supplement: S34 Fig — Nucleotide sequences of the genes, tentatively denoted Svsc1-Svsc4, are given with translated nucleotides highlighted in green, and non-translated in grey. The TATA box in the upstream promoter region is doubly underlined and translatations in one-letter code are written above the coding nucleotides. Two poly-Gln tracts in Svsc3 are highlighted with thick underlining. (DOCX) [file pone.0240607.s036.docx]

Kangaroo rat *Svsc4*

CTGGCATTAAAGTAGAAAGGGCAGTGCCTTTTAGCTTCAGCCACACCCTTGATACACCCTGTCCAGGAAGATATA

M K S T I I F I L S

AATGAGAAACAACCCTCAGCTCTCAGCTAAGACTCATATTGGCAAGATGAAGTCCACCATCATCTTCATCCTTTC

L L F V L E K Q A A G V A F Q

TCTGCTCTTTGTTCTGGAGAAGCAAGCAGCTGGTGTTGCGTTCCAAGGTGAGTGGGGTGGTTTGGGCAAGGGAAA

GATACTTCAAGGAGCAGACCCTCCACCTAAGGTTATTATTCTGGGGCTGAGCAGAAATTTCTTCAAGGCAGCCCT

TCTTCTTTTATGCTGGTACGAAGGCGTGAACTCAAGGGCTGGAGCGCAGTCCATTAGCTTTTACACTCAAGGCTG

GCAATCTACTACTTGAGCCAAACCTCTGTTTTCAGCAGCCCCTCTTCTTAATGAGAACAAGTCTTCACTAGAATT

CAGATGTCTTGGGGGTGGGTGGCAGGTGGGGGTGGAGGGTCATGAATGAATTGATGCAAGAGATCTGGGTAGGCT

G Q T K S Q

TGGAAGAAAGGAGAGGTGAGAGATGATGTAACATTTTTTTTATTATTATCAATTACTAGGTCAGACGAAAAGCCA

L P D R S Y E Y L L A Q Q K T H Q H V G Q K G A K

GTTGCCAGATAGATCCTATGAATATCTACTTGCACAACAAAAAACTCATCAGCATGTTGGACAAAAAGGCGCCAA

G I S S E E S F L T Q T K S Q M Q G S D L S M Q Q

AGGAATATCAAGTGAAGAAAGTTTTCTGACCCAAACTAAAAGCCAGATGCAGGGCAGTGATCTTTCTATGCAACA

T Q T K Q A Y V A K K Q A S L C Q A G G L S Q Q K

AACACAAACTAAGCAGGCCTATGTGGCCAAAAAACAAGCCAGCCTTTGCCAGGCAGGAGGTTTAAGCCAACAAAA

S A Q M I A T K H A G G Q T Q V H K H F D M T Q A

GTCCGCCCAAATGATCGCTACTAAGCATGCTGGTGGACAGACACAAGTCCATAAACATTTTGATATGACCCAAGC

K G R S G Q Y M K T K G S S L Y L G A K G A S Q L

CAAGGGAAGGTCAGGTCAATATATGAAGACAAAAGGAAGCTCACTCTACCTTGGAGCCAAAGGCGCTTCCCAGCT

K G T S Q Y M K T K G S S L Y Q G T K G T K F Q E

CAAGGGAACTAGTCAATACATGAAAACCAAGGGAAGCTCCTTATATCAGGGAACCAAAGGCACAAAATTCCAAGA

R Q V S F K G Q S Q Y P S D E Q M Q F V K G A Q I

GAGACAGGTCTCATTCAAAGGGCAATCACAATATCCCAGTGATGAGCAAATGCAATTTGTCAAGGGAGCTCAAAT

K Y Q D S M E Q Y L Q *

CAAATACCAAGACTCTATGGAGCAATATCTTCAGTAATACGCTGAAGACCAGGACAGATTTGGAGGTAAGTTTCT

TACTATACTATACAGGAGAGATACCTACCCTGATGTTTAGAAGTGGTAGATGAGGCCTTCATGGGATTATTTTAT

GGGATTGAGACTTCTTGTCTACTTCAATAGAAGTGCTGTGTTCCCAGGGCTTTTGGGGGGGGGCGGGAGGAGTAG

AGGGCGGGAGAGGTAGATGCGCTAGGCTCCTGATTAGAAGAATGAAAAGCTACCTTGGTTATCATTAGGCACTGA

ATTTCTACTCAGTAAATATCTGCGATGGTTTCCTTTTACCTAGATTTTAAAAAATAACTTTTTTTCTGACCATGG

ACCACCTAGATAAGCATATTTATAAGTTGATAGTGTAAGCATTGCTAATTATTAGACACCAGAGTAGTAAAATTT

TATTACTTGCTTATATTGATCTAAAGCATAAAAAATAATATTTTCTCTTTCTGCATACTGTTGAGCATGCTCGCA

TCCCTGTTTGGGTATAGCTGTCCTTCTCCAGAGAGCCCAGGTCCAAGGACTTCATTTGCTCTCTTTCTTTCAGAA

TATAGGGAAGGAATCCTAATACAGAGAAAGATGTCTGTCTGTGGCATCAGAGAGTTGACTAGAGAGGGAGAGACC

CCACTTTTCCCCATTGAAAATTCATAATGCCAGGAGCGAAATCTGAAACCCAATGAAGGAAAATGATGCTTCCTG

TAGGCAAGGAACATAGGCAATATCCTCAGCTGACTCCTGTGGTCTAGCATACTAATATGTGTGAGTTGGTTCAGC

TGATACCAAAAGGGACAGTGGTCAAATCTGACATCCAAACCTTCTCCATGATCACATTACTCACGGTCCTCTCTT

TCCCGAAGGGTCATCTTACCTGAGTGCAGTCTGTTACGCCTTCAAGATTCATTCTCCTGGGTGATTCCAGACCCT

TGGTCCATGGATGATGCCACCTCCTCACACTTGCTTTTCTTTGGGATTTCTAACATGTGCTTTTCAATAAAAACA

TAACTTTCTGCATCATTTGCTTTTGACTTCTGAGATTTTTTTGCTTTTTTCAGGTTTAGAGGGCTGGGAGACATC
