# Supplementary material for: Identification of the major rabbit and guinea pig semen coagulum proteins and description of the diversity of the REST gene locus in the mammalian clade Glires
Source: PLoS One. 2020 Oct 14;15(10):e0240607. doi: 10.1371/journal.pone.0240607 (PMC7556508; doi:10.1371/journal.pone.0240607)
Supplement: S35 Fig — The primary structure of Svsc1 aligned with that of Svsc2 are shown at the top. Below, Svsc4 is shown aligned with Svsc3, from which a central repeat region has been removed. Conserved residues are indicated by star symbols (*). Highlighted in grey are the conserved Cys, which are homologous with equally placed residues in myomorph Svs2 and Svs3. The Lys-Gly dipeptides in the C-terminal half of Svsc4 are highlighted in yellow and the tandem repeats are underlined. The central repeat region in Svsc3 is shown at the bottom, with the Gln-Leu-Lys motif highlighted in red. (DOCX) [file pone.0240607.s037.docx]

Svsc1 MKSTIFFILSLLFILQNQ-ASGGGFHGAGQNPLEGLKSKFPNRPRNHRHFGHHKRIE-SEMGSVGGGETRGVDGGTEMKY

Svsc2 MKSTTFFIISLLLILQEQIAVGIGYHGSPPGQLSNEIPAFPKRPRGSRGFGHHRTVHVTEDSSLSQPEPKVGDSDSDTKY

**** *** *** *** * * * * ** * ** *** * **** * * * * **

Svsc1 AV----SVFATEDRTKNRPNE----DLNESFGVNRGHRREHFESFHKRKERNSDGGFGFSKKKTVHIQHEHMQ

Svsc2 TLKEKETIISEEHRSENKPDSFSSGDSLENKVESRVHR--HFHERRHKTEKEDQGGYEFSKKRTIQYQHEHNE

* * * * * * * ** ** * ** **** * ****

Svsc3 MKSPI-FILSLLLILEKQAAGMPFYGQTKSQFPDRSYEHLLTQQIQQALQQQTQHHHEPTATKGVLAAEGIVTKTKSQVQ

Svsc4 MKSTIIFILSLLFVLEKQAAGVAFQGQTKSQLPDRSYEYLLA-------QQKTHQHVGQKGAKGISSEESFLTQTKSQMQ

*** * ****** ******* * ****** ****** ** ** * * ** * * **** *

Svsc3 TKDFGMPQQQQQQQQQQQQMTQQTIARED-TLCAAEGIKTQQLSKGISSQQS<- 261 AA repeat region ->KT

Svsc4 GSDLSM---------QQTQTKQAYVAKKQASLCQAGGLSQQKSAQMIATKHA--------------------------GG

* * ** * * * ** * * * *

Svsc3 QIHQQKEDYGPQAQRHVVQLVNTKESMLYHGPTQQQQQQ-----------------LYQVQ--YKQQLHGHSDQLKAAYL

Svsc4 QTQVHKHFDMTQAKGRSGQYMKTKGSSLYLGAKG-----ASQLKGTSQYMKTKGSSLYQGTKGTKFQERQVSFKGQSQYP

* ** * ** * ** * *** * * * *

Svsc3 SQGQCRCIKGIALKSIA-------

Svsc4 SDEQMQFVKGAQIKYQDSMEQYLQ

* * ** *

Central repeat region in Svsc3

HQKTLVLKGEPSQQKTLVVKGEESQKTLILTGDPNQQKTLLVQGAPSQQKTLVVTNEPSQLKTLVVTNEPSQQKTLVVTNEPSQLKTLVV

QGEPNQLKSLMVPDAPSQLKSLMVPGAPSQLKTLMVPGVPSQLKSLIVPGAPSQLKSLMVPGVPSQLKSLVVPGAPSQLKSLMVPGAPSQ

LKSLMVPGVPSQLKSLMVPGAPSQLKTLMVPGAPSQLKTLMVQGEPSQLKTLMVSGAPSQLKTLMVPGAPSQLKSLVVKGM
