# Supplementary material for: Identification of the major rabbit and guinea pig semen coagulum proteins and description of the diversity of the REST gene locus in the mammalian clade Glires
Source: PLoS One. 2020 Oct 14;15(10):e0240607. doi: 10.1371/journal.pone.0240607 (PMC7556508; doi:10.1371/journal.pone.0240607)
Supplement: S36 Fig — The alignment was done with Clustal Omega using default settings, followed by a minor manual adjustment. The residues shown N-terminal to the sign (<>) are encoded by exon 1 and those C-terminal to the sign are encoded by exon 2. The Cys duplets highlighted in red are in this setting specific for Wfdc15b in myomorph rodents. The location of the Trp highlighted in green separates Wfdc15c from Wfdc15d in hystricomorph rodents. NMR and DMR are abbreviations for naked mole-rat and Damaraland mole-rat. (DOCX) [file pone.0240607.s038.docx]

Jerboa Wfdc15a MRLGRPSLFAATILLCFHVTQPGIPSM---<>-------TPKRGLCPHFSLACPTLHPPLCWQDTECQGFDKCCFIDCQLRCVRPTKFSTK 79

DMR Wfdc15d MKLSSLSLLALTILLCLHMAQPGLRKNG--<>------VKQKPGFCPEFFLNCAFTGFPGCWSDRGCKGTKKCCFYNCRHQCIEPWSSVD- 80

NMR Wfdc15d MKPSSLSLLALTILLCLHVAQPGVRKNG--<>------IKQKPGFCPEFFLNCAFTGFPGCWSDRGCKGTKKCCFYNCRRRCTEPWSSVT- 80

DMR Wfdc15c MKLSSLSLLALTILLCLHMAQPGLRKNG--<>------TKWKPGYCPEFFLECAFNGFPGCLSDRNCKSGKKCCYYNCRHQCMKPALSVD- 80

NMR Wfdc15c MKPSSLSLLALTILLCLHVAQPGVRKNG--<>------TKWKPGFCPEFFLECAFNGFPGCRSDRSCKAGKKCCYYNCRHQCVEPALFLD- 80

Chinchilla Wfdc15c MKLTSLSLLALTILLCLHMAQPGIRKNG--<>------TKWKPGYCPEFYLECIFSGFPGCLTDRSCKGNKKCCYYNCRHQCMEPSLSLD- 80

Guinea pig Wfdc15c MKLSSLSLLALTVLLCLHVAQAGLRKNG--<>------TKWKPGFCPEFGLDCIFNGFPGCLSDRSCKGIKKCCYYNCRHQCMDPS----- 76

Degu Wfdc15c MKLTSLSLLALTVLLCLHEAQPGLRKNG--<>------TKWKPGHCPEFYLECMFSGLPGCLSDRSCKGTKKCCYYNCRHQCMDPSMTVD- 80

Degu Wfdc15d MKPTSLSLLALTVLLCLHEAQPGLWRKR--<>------VKRKPGFCPEFSLECIFIGLPVCWNDRSCRGSKKCCFYNCHLQCMEPWFSLD- 80

Chinchilla Wfdc15d MKLPRLSLLALTVLLCLHVAQPGFWRNR--<>------AKQKPGYCPEFFLHCAFTGLPQCWGDQSCKGSKKCCFYNCQLQCVEPRSSLD- 80

Pika WFDC15 MKLRCLSLVTVGVLLCLHSAQPGIMRAARP<>------VNPKPGFCPEFHPECPFTLFPSCRHDQSCKGAKKCCFYACRRQCVEPWQTLD- 82

Rabbit WFDC15 MRPSRASLLALCVLLCLPGAQPGVMRRA--<>------ADPKPGYCPEFFPECPFTLFPSCRRDQGCKGAKKCCFYQCRRQCKEPWLSLD- 80

Ground squirrel Wfdc15 MKLSSLSLLSVAALLWLRVAQANMKE----<>------VQQKSGYCPEFFLSCSFTLLPRCRRDGGCNGPKKCCFYQCQQQCMQPWPTLD- 78

Jerboa Wfdc15b MKLSRLSLLIVTIFLCYDMAQSGVNRK---<>-----ALTPKPGYCPEFPQSCPFVIIPFCKYDRGCKGRKKCCFFYCQYKCVDPWLVSN- 80

Kangaroo rat Wfdc15 MKHSSLFLLMVTVLLCLHLAQPRFLWRR--<>------GTEKSGYCPEFHLDCRFTLLPKCNHDRGCKEDRKCCFYYCRKQCVKPWWTLN- 80

Hamster Wfdc15a MKPSSLILLTTTILLCLNMVQPKHT-----<>ATRKPPKTSKPGFCPEYFVDCPFIRLPLCKKDKGCKGNKKCCFYDCQMHCMEPWISMD- 83

Deer mouse Wfdc15a MKPRSLTLFTTTILLCLSMVQPRIKKKR--<>-----VTTPKPGYCPEFFLPCPFVRLPVCKLDKGCRGIKKCCFYYCQMRCVEPWTTAT- 81

Vole Wfdc15a MKPRSLTLFTTTILLCLSMVQPRIKRK---<>------VTPKPGYCPEFFLPCPFELLPVCQHDKGCKGIKKCCFYYCQMRCVEPWTTTF- 79

Rat Wfdc15a ---NSLLQFTTTTLLCLSMVRLSVTRKG--<>------VTPKQGYCPEFLLNCPFVLLPLCNRDSGCKGTKKCCFYYCQMRCVEPWTSLT- 77

Mouse Wfdc15a MKPSSLLLFTTTILLCLSMAQPRATRKG--<>------VTPKQGYCPEFLLDCPFVLLPVCSRDKGCKGTKKCCFYYCQMRCVEPWTTLT- 80

Mouse Wfdc15b MKLLGLSLLAVTILLCCNMARPEIKKKN--<>------VFSKPGYCPEYRVPCPFVLIPKCRRDKGCKDALKCCFFYCQMRCVDPWESPE- 80

Deer mouse Wfdc15b MKSLSLSLLTVTVLLCCNITQPRFWERT--<>------VIVKSGFCPEYHLSCPFVLLSKCKRDRGCKGNKKCCFYNCQMRCVEPWATLD- 80

UGMBMR Wfdc15b1 MKLISLSLLTVTILLCCNMAQPKFKNN---<>------VTTKPGYCPEFHLSCPFVLLPVCRYDRGCKGDKKCCFYYCQKRCVEPWDSMY- 79

UGMBMR Wfdc15b2 MKLISLSLLTVTILLCCNMAQPKFKNN---<>------VTTKPGYCPEFHLSCPFVLLPVCRYDRGCKGDKKCCFYYCQKRCVEPWDSMY- 79

Rat Wfdc15b MKLLSLPLLTVTILLCCNMAQAIFWRKK--<>------ALSKPGFCPEFHLPCPFVLVPKCRRDRGCSGSLKCCFYYCQMRCVVPWDNSD- 80

Hamster Wfdc15b MKPLSLSLLTVTILLCCDMTQPTFRKGM--<>------AVDKPGYCPEFHLSCPFTLLPICWRDRSCRGAKKCCFFYCQMRCVEPWHTLD- 80

Vole Wfdc15b MKPLSLSLLAVTILLCCNMTQPIFRRNK--<>------ALIKPGYCPEFHLSCPFVLLPLCKRDIGCKGAKKCCFYYCQKRCTDPWNTLN- 80

* * * ** * * * * *** * * *
