# Supplementary material for: Identification of the major rabbit and guinea pig semen coagulum proteins and description of the diversity of the REST gene locus in the mammalian clade Glires
Source: PLoS One. 2020 Oct 14;15(10):e0240607. doi: 10.1371/journal.pone.0240607 (PMC7556508; doi:10.1371/journal.pone.0240607)
Supplement: S37 Fig — Sequences surrounding the splice donor site in WFDC12 are aligned with exon sequences highlighted in green and translations written in one-letter code. As can be seen, the splice phase is the same in mouse and kangaroo rat, which differ from the splice phase in jerboa and human WFDC12. The other analyzed rodent and lagomorph WFDC12 had the same phase as the human gene, except for the rat, which had the same phase as the mouse and kangaroo rat genes. (DOCX) [file pone.0240607.s039.docx]

Exon 2 Intron 2

Q C G F K C V L P V K D N S E E

Mouse CAATGTGGCTTCAAATGCGTGCTGCCGGTGAAAGACAACAG-TGAAGAGTAAGTAGCTCCCAGTGCTG-TGCGCGGCCCTCCCACCTCCCCAGTCTCTGT

|| |||||||| | || | | ||| |||| |||| | | || ||||| ||| | | | | | | | ||| ||||| ||||| | | |

Kangaroo rat CACTGTGGCTTTACCTGTATACAGCCTGTGAG-GACACTGGATCGAGGGTAAGGAGCCCGCTGCTCACCTCCCCTGCCTACCCACACCCCCACCCCCAGC

H C G F T C I Q P V R T L D R G

H C G F T C I Q P V R T L D R G

Kangaroo rat CACTGTGGCTTTACCTGTATACAGCCTGTGAGGACACTGGATCGAGGGTAAGGAGCCCGCTGCTCACCTCCCCTGCCT-ACCCAC--ACCCCCACCCCCAGC

||||||||||| | ||| | ||||||| | |||||| || ||||||||| || || | || | || | || | | || | | |

Jerbo CACTGTGGCCTCAAGTGTGTGATGCCCGTGGAGACACTGGA-CGAAGGTAAGGAGCCC-CTGC-CCCTAGACGTGGCTCTCCCACTGGGTCCCACCCCCCGC

H C G L K C V M P V E T L D E

H C G F T C I Q P V R T L D R G

Kangaroo rat CACTGTGGCTTTACCTGTATACAGCCTGTGAGGACACTGGATCGAGGGTAAGGAG-CCCGCTGCTCACCTCCCCTGC-CTACCCACACCCCCACCCCCAGC

||||||||||| | ||| | ||||||| | |||||| || ||||||||| || || | || | || | || | | || | | |

Human CACTGTGGCTTCAAGTGTGTGATTCCTGTGAAGGAACTGGA-AGAAGGTAAGGAGACCTGCCTCCCAGGGCTGGGGCTGTCCCTTCCCTGCCTCTATCTGA

H C G F K C V I P V K E L E E
