# Supplementary material for: Identification of the major rabbit and guinea pig semen coagulum proteins and description of the diversity of the REST gene locus in the mammalian clade Glires
Source: PLoS One. 2020 Oct 14;15(10):e0240607. doi: 10.1371/journal.pone.0240607 (PMC7556508; doi:10.1371/journal.pone.0240607)
Supplement: S38 Fig — The aligned primary structures of apparently functional rodent Tgm4 are shown. The amino terminals were heterogeneous due differences in the location of start codon. Residues at the catalytic site are highlighted in blue and additional residues of importance for the catalytic activity of transglutaminases are highlighted in yellow. The fullength sequence of kangaroo rat was not available, but residues of importance for catalysis are present, as can be seen. (DOCX) [file pone.0240607.s040.docx]

Kangaroo rat --------MTTMNELQALRIDFLRKQNTTAHHTDNFQTN------KLVLRRGQVFHLRLMLSKSLEP-HEQLKLQFAIGPNPSIAKHTLVELNLKMPTASRFEGWKASLTSNTGREVIVA 105

Guinea pig --MADNKQETMAEELQIRRVDFMRRDNTAAHHTAEFQTP------ALVLRRGQAFTLRLKLNQRLRSQQDTLTLQFSMGRQPCVSKHTLISLDLPSSASCHGWKASIRSEAGL--EVVIA 111

Degu --MADDMQETMAEELQIYHVDFMKRDNTAAHHTADFQTP------GLVLRRGQPFTIRLELSQALRTSQDALILQFSMGHRPSVSRHTLISLDLQSSAGCHGWKASIHSKAGL--EVVIV 110

Chinchilla ----------MAEELQIRHVDLMKQDNAAAHHTADFQTP------ALVLRRGQVFTLRLQLSQPLQPGQDHLTLQFSMGLEPSVSRHTLISLDLQSSASWHGWRASIRSQAGS--EVVIA 102

Jerboa MDCDSLISEDFTMRLQIIQVNLRKKANSIAHHTSDFQNE------KLVLRRGQVFHLLVVLNRPLGP-QDQLMLQFITGKKNAISRHTMVELNARTSNLSQDWKATISKVSGS--EVTVA 111

UGMBMR ------MDTENKKELNFIHVWFKTKKNAQAHHTSNYKDSNNEKETIDVLRRGQAFTLFLKTNRHLQN-QDHVRLQFLSGLDYS---YMVVELSLVPSSSSQGWHLKRIKQSDAKREVKII 110

Mouse --------MDSRNVLIIYAVNVERKLNAAAHHTSEYQTK------KLVLRRGQIFTLKVILNRPLQP-QDELKVTFTSGQRDP---PYMVELDPVTSYRSKGWQVKIAKQSGV--EVILN 100

Rat --------MDSRNMLVVYSVNLEKKLNAAAHHTIEYQTQ------KLVLRRGQIFSLKVMLNRPLQS-HDELKLIFNTGHNMP---FYTVELDPMTSYRSKGWQVKIAKQSGV--EVVLN 100

Hamster --------MDTISALKVSSVDMEKKENMAAHHTSEFQTK------NIVLRRGQIFNMKVILNRPLQP-QDELKLIFS-IGQNS---SKYVELDPMTSFRSQGWHVKIAKQSGS--EITLS 99

Vole --------MDSRSALVVYSVNLEKKENAAAHHTSEFQTQ------KLVLRRGQIFNIKVVLNRPLQP-QDELKLVFS-TGERM---SHFVELDPMTSFRSKGWQVKITKQSGG--EVTLA 99

Deer mouse --------MDTRKALVVYSVNMEKKENAAAHHTLDFQTT------KLVLRRGQLFNMKVILNRPLHP-QDELKLTFFVRGSKE---SSLVELNPMTSFRSKGWQVKIAKQSGE--EVKLT 100

* . * . : * **** ::: ****** * : : .: * :* : : * :.*. * :.*: . . :. *: :

Kangaroo rat ITSAPNSIVGKYEMAVKT-GSHTFR-VHEDFYLLFNPWCIDDPVFLPDEEERKEYILNDTGYIYMGFAKHIRGKPWNFGQFEKNILSCAIYLLTQSYLKILEMRDPVLVARTMCAMMSSA 223

Guinea pig VTSAPNAIVGLYSLELKS-GRHLLKPEDSMVYLLFNPWCPEDAVFLPSEEDRAEYVLSDTGYVYMGSFRQVKEKPWNFGQFEKNVLDCCIFLMTQSYLKTMDMRDPVMVGRNMGAMINSQ 230

Degu VTSAPNAVVGQYSLEVMS-GRHVFKPEEDTMYLLFNPWCADDAVFLPTEEDRAEYILSDTGYLYMGSFRQVKEKPWNYGQFEKNVLDCCIFLMTQSYLKTVDMRDPVMVGRNMAAMVNSQ 229

Chinchilla VTSAPHAIVGVYSLEVKS-GRHLFEPEDSTVYLLFNPWCPDDAVFLPTEEERAEYILSDTGYVYMGSFRQVKEKPWNYGQFEKNILDCCIFLMTQSYLKTMDMRDPVMVGRNMGAMVNSQ 221

Jerboa VTSSPDALVGKYRLSVKC-GNHTFK--CEKIFLLFNPWCKEDKVFMPNEEERTEYVLNDTGYIYMGVTKQIRGKPWIFGQFEKHILNCCLSLLKPHIIKTTDMNDPVEVSRSMCTMMSSA 228

UGMBMR VTSPPDAMVGKYKLCMKRDGSRSIM--SKEFFIIFNPWCAEDTVFMPNEEDRAEHVLNDTGYIYMGFAKQIKEKPWTFGQFEKHILHCCFSLLTH--LQPRELQSPVLVSRSICTMMCAV 225

Mouse VISAADAVVGRYKMRVNE-----YK--AGVFYLLFNPWCSDDSVFMASEEERAEYILNDTGYMYMGFAKQIKEKPWTFGQFEKHILSCCFNLLFQ--LENNEMQNPVLVSRAICTMMCAA 211

Rat VISAADAVVGRYTMNVNE-----FD--AGVFFLLFNPWCSDDSVFMASEEDRAEYVLNDTGYMYMGFAKQIKEKPWTFGQFEKYILNCCFRLLTH--LEPKEMQSPVLVSRAICTMMCAA 211

Hamster VISAADAVVGQYSLYINS-----HD--AGSFFLLFNPWCADDTVYLPSEEERAEYILNDTGYIYMGFAKQIKEKPWTFGQFEKSVLDCCMYLLSN--LTPHELQSPVSVSRTICTMMCSV 210

Vole VISAADAMVGKYYLKVNE-----SK--GGIFFLLFNPWCADDSVYMPSEEERAEYILNDTGYIYMGFAKQIKEKPWTFGQFEKHILKCCFSLLTH--LEPSELQSPVIVSRTICTMMCAV 209

Deer mouse VISAADAMVGRYKLRVND-----YK--VGEFFLLFNPWCADDNVYMPSEEDRAEYVLNDSGYIYMGFAKQIKEKPWTFGQFEKHVLSCCFSLLEQ--LEHVDMQSPVNVSRNICTMMCAA 211

: * .*:** * : : .:::***** :* *:: .**:*:*::*.*:**:*** :*:: *** :***** :* **: *: : ::..** *.* : :*: :

Kangaroo rat NSNGVLTGNWSGNYAGGTAPHLWSSSVAILQQFHHTKQPVNFGQCWVFSGILTTVLRALGIPARSVTAFESAHDTEKDLTVDIYLNQTGKTIPDLTKDSVWNFHVWTDAWMRRPDLPVGN 333

Guinea pig NKDGVLVGNWTGEYQGGMAPYLWTGSVPILQRYYSTREPVSFGQCWVFAGVLTTVLRALGIPARPVTVFDSAHDTEENLTLDVYVNEMGKTVPNKTDDSIWNFHVWTEAWMKRMDLPPGN 350

Degu NKDGVLVGNWSGEYEGGTAPHLWTSSVPILKQYYSSRTPVSFGQCWVFAGVLTTVLRALGIPARPVTAFDSAHDTEENLTLDMFVNEMGKTVSNKTNDSIWNFHVWTEAWMRRRDLPPGN 349

Chinchilla NKDGVLVGNWSGEYQGGTAPHLWTGSAPILQRYYSTRTPVSFGQCWVFAGVLTTVLRALGIPARAVTVFDSAHDTEENLTLDMFVNEMGKTVPNKTNDSIWNFHVWTEAWMKRKDLPPGN 341

Jerboa N-KGVLVGNWSGDYSKGTAPYVWTSSVPILQQYYTTKMPVNFGQCWVFSGILTTALRALGIPTRSVTNFESAHDTEKNLTVDIFLDENGKTIGTMTRDSVWNFHVWNDVWMMRSDENGDN 347

UGMBMR N-NGVLVGNWSGDYSNGTAPYVWTSSVPILQQHYVTGLPVCFGQCWVFSGILTTALRAVGIPARSVTNFESAHDTGKNLTVDIYLDDSGQTIGDLTKDSVWNFHVWADAWMKRPDLPQGN 345

Mouse N-GGVLMGNWTGDYADGTAPYVWTSSVPILQQHYVTRMPVRYGQCWVFSGILTTALRAVGIPARSVTNFESAHDTEKNLTVDIYLDESGKTIPHLTKDSVWNFHVWTDAWMKRQDLPHGY 330

Rat NNFGVLVGNWTGDYSNGTAPYVWASSVPILQQHYITRMPVRFGQCWVFSGVLTTALRAVGIPARSVTNFESAHDTEKNLRVDIYLDESGKTIPHLTKDSVWNFHVWTDAWMKRQDLPQGH 331

Hamster N-NGVLMGNWSGDYKNGTAPYVWTSSVPILQQHYATKLPVCFGQCWVFSGILTTALRAIGIPARSVTNFESAHDTGKNLIVDIYLDESGKTNTDLTKDSIWNFHVWTDAWMKRQDLPPGH 329

Vole N-NGVLVGNWSGDYSKGTAPYVWTSSVPILQQHFITKEPVCFGQCWVFSGILTTALRAVGIPARSVTNFESAHDTGKNLIVDIYLDESGKTITHLTKDSVWNFHVWTDAWMKRQDLPQGH 329

Deer mouse N-NGVLVGNWSGDYSNGTAPYVWASSVPILQQHYFTKLPVCFGQCWVFSGILTTALRAVGIPARSVTNFESAHDTEKNLTVDIYLDESGKTIAHLTKDSVWNFHVWTDAWMKRRDLPQGN 330

* ***:***:*:* * **::*:.*.***:::* : ** :******:*:***.***:***:* ** *:***** :** :*::::: *:* * **:****** :.** * * .

Kangaroo rat DGWQALDGTPQEISH--------------------------------------------------------------------------------------------------------- 358

Guinea pig DGWQVVDGTPQELSQGLYCCGPSPVAAVRKGNIFMGYDTKFVYSEVNADKLVWLVRSVDGREKVSLLSVETMSIGKNISTKAAGQDRRSDITHQYKFPEGSAEEREVMDHAFSLLSFKRE 470

Degu EGWQVLDGTPQELSQGIYCCGPSPVAAVRRGDIFMGYDTKFVFSEVNADKLVWLVRTVGEREKVSLLSVETMSIGKNVSTKAVGQDRRSDITHQYKFPEGSREERAVMDHAFSLLSFRRE 469

Chinchilla DGWQVVDGTPQELSQGLYCCGPSPVAAVRRGNIFMGYDTKFVFSEVNADKLVWLVRTVGRREKVSLLSVETMSIGKNISTKAVGQDRRSDITHQYKFPEGSSDERAIMDHAFSLLSFRRE 461

Jerboa HGWQALDGTPQEISQERFRCGPAPLTAIRKGDVAVKYDTKFIFTEVNGDKLIWLVKQTPEDKKFILIAVETMSIGKNISTKAVGQDQRVDITYQYKYPEGSPEERQAMNKASNLLNKVVE 467

UGMBMR DGWQVLDSTPQEISDGGFRAGPAPLSAIRQGNVLLKYDTKFIFTEVNGDKLIWLVKQDQGRDKNTLIAVETMSIGKNISTKMVGQNRRQDITSEYKYPEGSPEERKAMERASGRLHPGED 465

Mouse DGWQVLDSTPQEISDGGFRTGPSPLTAIRQGLIQMKYDTTFVFTEVNGDKFIWLVKQNQEREKNILIAVETASIGKKISTKMVGENRREDITLQYKFPEGSPEERKVMAKASGKPS--DD 448

Rat DGWQVLDSTPQEISEGQFRIGPSPVSAIRQGLVQIMYDTTFVFTEVNGDKYIWLVKQNQEREKNVLIAVETASIGKNISTKMVGENRRQDITLHYKFPEGSPEERKAMEKASGKRP--DD 449

Hamster DGWQVLDATPQEISEGGFRTGPSPLSAIRQGAVQIQYDTKFVFTEVNGDKFIWLVKQKEGKEKNVLIAVETASIGKNISTKMVGENKREDITLQYKFPEGSPEERKAMEKASGKHT--DE 447

Vole DGWQVLDATPQEISEGGFRTGPSPLSAIRLGKVEIQYDTKFVFTEVNGDKFIWLVKQNQGREKNVLIAVETASIGKNISTKMVGENKREDITLQYKFPEGSPEERKAMEKAAGKHP--DE 447

Deer mouse DGWQVLDATPQEISEGSFRTGPSPLSAIRQGAVQTQYDTKFVFTEVNGDKFIWLVKQNKGK---VLLAVETASIGKHISTKMVGENRREDVTLQYKFPEGSPEERKAMDRASGKRP--DE 445

.***.:*.****:*: : **:*::*:*:* : ***.*:::***.** :***: *::*** ****::*** .*:::* *:* .**:**** :** * :* . :

Kangaroo rat ------------------------------------------------------------------------------------------------------------------------

Guinea pig H-TIPTKENLLELLVQEEPVVMGDPLNFSVTLRRKAATPQTITFSGSFDLQSYTGRQLAHLGVVQKTVQVQEQVSSVVLTWEASTYTGSLDAYEDEPVIKGYIMAEVMETN-ETIATEVS 588

Degu Y-TLPVKENLLELLVQEDPVLLGDPLHFSVTLRRKAATPQTIVFSGSLDLQSYTGRQLGHLGVVQKTMKVQEKVSNVVLTWESDTYTGSLDTFDDEPVIKGFIMAEVVETK-ETMATEVL 587

Chinchilla Y-TLPTKENLLELLVQEEPVRLGDPLNFSVTLRRKAATPQTVIFSGSFDLQSYTGRQLAHLGVVQKTVHVKEQVSNVVLTWESDTYTGSLDAFDDEPVVKGFIMAEVTETQ-ETIATEVS 579

Jerboa HK-PPTREDFYHISVTEDSVQLGDPIILTIVLKRNMVSLKNVNISCSLDLQTYTGKRVASLGVIQKTVQIQNPETEVVLTMNASSYINSLGMLDDELVIKGFAIAEVVGSNNEMVATEVS 586

UGMBMR QPGSPSTDSSLRVSVIECSVEIGHPIILTLVLERKTATPQTVNISCYLNLQTYTGKKKKGLGIIRKSQKITNQESQVTLTMDTNSYIHNLGMVDDELVIKGFIITEIAGSR-ERVATEVT 584

Mouse KLNSRTLNNSLQISVLQNSLELGAPIYLTITLKRKTATPQNVNISCSLNLQTYTGNKKTNLGVIQKTVQIHGQESRVFLTMDASYYIYKLGMVDDEMVIGGFIIAEIVDSG-ERVATDTT 567

Rat KLNSRTL----HISVLQNSVELGHPINLTIVLKRKTATPQNVNISCSLDLQTYTGNKKTNLGVIQKTVQIQGQESEVSLSMDSSFYIYKLGMVDDEMVIKGFIIAEIVDSG-ERVATDTT 564

Hamster DKPNRSSSNSLKIHVLQNSVELGYPVTLTLALKRKTDVPQNVNISCSLDLQTYTGGNKTNLGIIHKTVHVQGQESEVVLNMAADSYIYKLGVFDDEMVIKGFIIAEVMETG-DKAATDMT 566

Vole DKPGRRPVNSMLQIHVLQNSVELGYPVTLTLVLKRKTATPQNVNISCSLDLQTYTGSQKTNLGIIQRTVQIQGQESEVVLSMDANSYIYKLGMVDDEVVIKGFIITEIVETGDKVATDTT 567

Deer mouse NKPTLPHNSFLQIHVRQNSVELGYPITLTLVLKRKTHTPQNVSISCSLDLQTYTGNKKTNLGVIQKSVQIQGQESEVALSMDANSYIYKLGMVDDEVVIKGFIIAEITETG-DKAATDTT 564

.: * : : :* *: :::.*.*: :.: :* ::**:*** . **:::*: :: : * *. :. * .*. :** *: *: ::*: : : **:

Kangaroo rat -------------------------------------------------------------------------------------------------------

Guinea pig LFFQYPQLTLELPNTGRMGQALVGTCVFRNSLLIPLTGVRFSVESLGLSSSQSIEHGTVAPGETVQSQISCTPVRLGLRKVIVKLSSQQVKEVHAEKVVLVTP 691

Degu LCFRYPLFTIEMPNTGRIGEVLLCTCTFKNSLMIPLTHVRFSVESLGLSSLQSIEQGTVPPGKTIQSQIRCTPVRAGPRKFIIKLNSQQVKEIHAEKVVLITQ 690

Chinchilla LCFRYPLFTIELPNTGRVGKELVCTCIFRNSLLIPLTQVRFSIESLGLSSVQSVKQGTVPPGQTVQSQIRCVPVRAGPRKFIIKLSSLQVKEIHAEKVVLITQ 682

Jerboa LSFLYPDFFIEMPNTARVDQPLIFTCSFKNSLPIPLTEIKFSVESLGISSMKTMDQGVLPPGKSIQFQMNCVPVKLGPSKFIIKFTSRQVKEVHAEKMVLITN 689

UGMBMR LCFLYQGFHIEMPSTGKVHQELLLICKLKNTLPIPLTNMRFSVESLGIVTTESLDQGTLPPGQSLQFEMRCIPMRTGPRKIIVRFTSREVKEVHVEKMVLITD 687

Mouse LCFLYSAFSVEMPSTGKVKQPLVITSKFTNTLPIPLTNIKFSVESLGLANMKSWEQETVPPGKTITFQMECTPVKAGPQKFIVKFISRQVKEVHAEKVVLISK 670

Rat LCFLYSAFSVEMPSTSKVNQPLTITCNFKNTLPIPLTNIKFSVESLGLNNMKSWEQETVPPGKTINFQIECTPVKTGPRKFIVKFISRQVKEVHAEKVVLITK 667

Hamster LSFLYPAFSVEMPDTCKISWPLTIKCSFKNTLPIPLTDIRFSVESLALSHMQSWEQGTVPPGKFIHFQMKCTPEKIGPRKFIVKFTSRQVKDVHSEKIVLITN 669

Vole LSFLYPAFSVEMPDTGKVNWPLLITCTFKNTLPIPLTNIRFSVESLALSNMQSWDQGTVPPGQSITFQMKCTPVKSGPKKFIVKFTSRQVKEVHAEKIILITK 670

Deer mouse LCFLYPAFSVEMPSTGKINMPLSFNCSFKNTLPIPLTNIRFSVESLALSKMQSWKQGTLSPGMSLNFQMKFTPVRTGAKKFIVKFTSREVKEVDAEKIILIT- 666

* * * : :*:*.* :: * . : *:* **** ::**:***.: :: .: .: ** : :: * : * *.*::: * :**::. **::*::
