# Supplementary material for: Identification of the major rabbit and guinea pig semen coagulum proteins and description of the diversity of the REST gene locus in the mammalian clade Glires
Source: PLoS One. 2020 Oct 14;15(10):e0240607. doi: 10.1371/journal.pone.0240607 (PMC7556508; doi:10.1371/journal.pone.0240607)

Figure 1

The image was generated by scanning of the stained gel. Figure 1 was created by omitting the lanes marked with X.

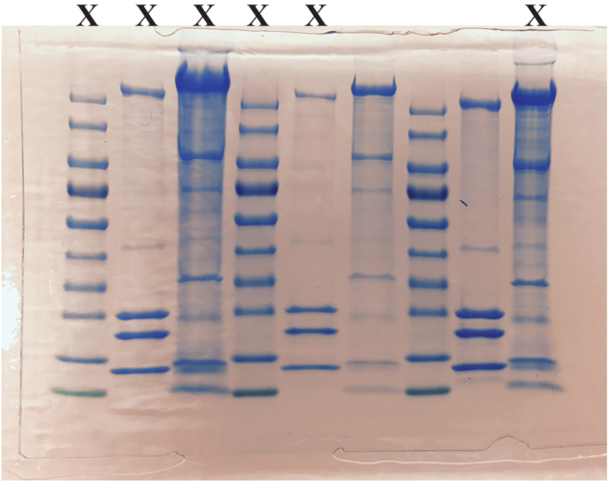

Figure 2A

The image was generated by scanning of the stained gel. Figure 2A was created by omitting the lanes marked with X.

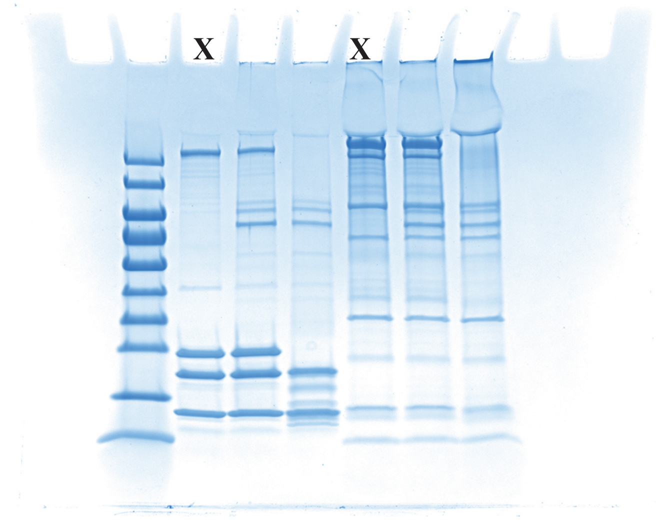

Figure 2B

The image was generated by scanning of the un-stained gel illuminated by UV light. Figure 2B was created by omitting the lanes marked with X.

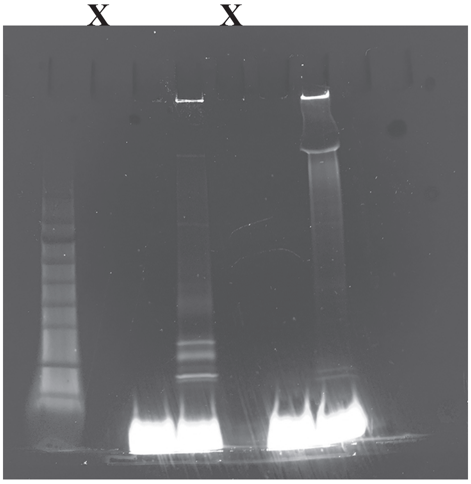

Supplement: S1 Raw images — (PDF) [file pone.0240607.s041.pdf]
