# Supplementary material for: Identification of the major rabbit and guinea pig semen coagulum proteins and description of the diversity of the REST gene locus in the mammalian clade Glires
Source: PLoS One. 2020 Oct 14;15(10):e0240607. doi: 10.1371/journal.pone.0240607 (PMC7556508; doi:10.1371/journal.pone.0240607)
Supplement: Attachment — Submitted filename: Response to Reviewers.docx [file pone.0240607.s042.docx]

Response to the Academic Editor and the Reviewers

Academic Editor

We have considered the reviewers’ suggestions and made changes to the manuscript. The title has also been slightly modified to ‘Identification of the major rabbit and guinea pig semen coagulum proteins and description of the diversity of the REST gene locus in the mammalian clade Glires’. We did not feel that the manuscript could be shortened without losing vital information, but we have introduced new subheadings that we think will improve the readability of it. There is sufficient information in the manuscript to independently reproduce our experiments. The information regarding software and databases was in our original submission unfortunately placed in a context, such that it could lead to confusion and misunderstandings. It has now been moved to a more appropriate place.

Reviewer 1

The reviewer refers to Glires as clade Glires, even though it has also been ranked as a grandorder. After careful consideration, we have decided to use the term clade in order to describe Glires. Hence, the title of the paper has also been changed.

Response to comment 1

Our data undoubtedly show that a new gene was created by the merger of SEMG2-like and PI3-like genes in ancestors of both hystricomorph species and rabbits. This could, of course, have happened by two independent events, but according to the principle of parsimony, it is more likely that it happened in a common ancestor. As there were no signs of a similar gene merger in any of the other analyzed species, it also seems more likely that the merger happened after the separation of hystricomorpha from myomorpha and castorimorpha. We are fully aware that this might be controversial and in an effort to avoid being provocative, we have changed the wording of the last sentence in the abstract from ‘hystricomorpha separated from myomorpha’ to ‘hystricomorpha may have separated from myomorpha’.

Our study also hints at a close relation between sciuromorph species and pika, both of which are lacking functional SCPs (and also Wfdc12). This is not discussed in our paper as we deemed it too speculative. However, both this finding and the gene merger in hystricomorpha and rabbit suggest that it might be worthwhile to critically scrutinize the phylogeny of Glires. This is not our area of expertise, but it is our hope that the findings in this paper will stimulate, not only to studies on SCPs evolution, but also to extended studies on the phylogeny of Glires.

Response to comment 2 and 3

The manuscript is relatively, but not extremely, long. We do not feel that it is verbosely written. Instead, we think that the length very much reflects the content and most likely condensation would mean removal of interesting parts of the presentation. We do not agree to this – see also our response to comment 4. To ease up the reading, we have introduced subheadings in the Material and Methods and Discussion sections and also introduced new or modified old subheadings in the Results section.

Response to comment 4

In our view, reporting only the structures of Svp5 and SVP200 would generate a thin paper. Questions regarding the relationship between Svp5 and SVP200 also prompted us to widen the study and analyze REST genes in all rodents and lagomorphs that we could find in sequence databases. This study showed that 3 out of 4 rodent suborders had unique sets of REST genes, whereas the forth suborder seemed to be lacking them. We also noted that the SCPs were pseudogenes in eusocial rodents, which led us to also investigate the prostate transglutaminase TGM4, which in turn showed that all species lacking a functional SCP also carried a non-functional TGM4. We think that by extending the investigation, we have made discoveries that have transformed an ordinary paper to a very interesting paper that we have no intension to split up.

Reviewer 3

Response to comment 1

There is a flow chart on lines 316-324 in the manuscript. We think that moving that flow chart into Figure 1 would only complicate the figure and perhaps also the reading.

Response to comment 2

Subheadings have been introduced in Material and Methods.

BLAST is perhaps best known as a tool to search for homologies in sequence databases, but it can also be used to compare two sequences. If the web-based program at NCBI is used for the latter purpose, the output is aligned sequences and a dotplot. Dotplots created that way were saved and used in our paper. We think the procedure is appropriately described in the Material and Methods section, but we can also understand that it could lead to misunderstandings and confusion because of where it the text it was placed. It has been moved to a new location under the subheading ‘Study of the REST gene locus in rodents and lagomorphs’.

Response to comments 3-5

The biological function is, of course, an important aspect. Experiments showing that both Svp5 and SVP200 are excellent substrates of transglutaminase were made, but apart from that, function was not primarily addressed in this study. At the end of discussion (line 1068-1081) we address some functional issues, *e.g*. barrier function of SCPs.

Response to comment 6

We are presently not ready to sketch a hypothetical phylogenetic three, as there are too many options. Classic molecular phylogenetics is, as far as we understand, out of the question when it comes to comparing rodent suborder and lagomorpha, because of the vast sequence differences. It is not an easy task even within suborders, partly because of large sequence differences, but also because of non-random mutations, *e.g.* the homogenizing process affecting myomorph Svs3, Svs5, and Svs6.
